# Pharmacogenomic predictors of proton pump inhibitor response in pediatric eosinophilic esophagitis

**DOI:** 10.3389/fphar.2026.1913372

**Published:** 2026-10-30

**Authors:** Mariamena Arbitrio, Renato Tambucci, Valeria Tallarico, Ilaria Gnasso, Francesca Scionti, Giuseppe Agapito, Emanuela Altomare, Mario Cannataro, Graziella D'Arrigo, Giovanni Tripepi, Daniela Concolino, Fabiola De Masi, Maria Giovanna Puoti, Francesca Rea, Monica Malamisura, Alessandra Maria Ricci, Maria Cristina Artesani, Sara Urbani, Veronica Calandrelli, Alessandro Giovanni Fiocchi, Paola De Angelis, Licia Pensabene

**Affiliations:** 1 Department for the Promotion of Human Science and Quality of Life, San Raffaele Roma University, Rome, Italy; 2 Institute for Biomedical Research and Innovation, National Research Council (CNR), Catanzaro, Italy; 3 Gastroenterology and Nutrition Unit, Bambino Gesù Children’s Hospital, IRCS, Rome, Italy; 4 Pediatric Unit,“Renato Dulbecco” University Hospital, Magna Graecia University (UMG), Catanzaro, Italy; 5 Department of Experimental and Clinical Medicine, Magna Graecia University, Catanzaro, Italy; 6 Department of Legal, Economic and Social Sciences, Magna Graecia University, Catanzaro, Italy; 7 Department of Medical and Surgical Science, Magna Graecia University, Catanzaro, Italy; 8 Institute of Clinical Physiology (IFC-CNR), Section of Reggio Calabria, Reggio Calabria, Italy; 9 Department of Paediatric Gastroenterology and Hepatology, Santobono-Pausilipon Hospital, Naples, Italy; 10 Allergology Unit, Bambino Gesù Children’s Hospital, Rome, Italy

**Keywords:** ADME genes, drug response variability, eosinophilic esophagitis, machine learning, proton pump inhibitors

## Abstract

**Background:**

Genetic variability has been implicated in the heterogeneous response to proton pump inhibitors (PPIs) in eosinophilic esophagitis (EoE), with variants in *CYP2C19 and STAT6* previously associated with treatment outcome. However, the broader pharmacogenomic determinants underlying interindividual variability in PPI response remain incompletely understood.

**Methods:**

This study aimed to investigate whether genetic variability in Absorption, Distribution, Metabolism, and Excretion (ADME) genes contributes to PPI response in pediatric EoE. Fifty-eight patients (17 responders, PPI-RE, and 41 non-responders, PPI-NORE) were genotyped using the Drug Metabolizing Enzymes and Transporters (DMET) Plus microarray, covering 231 ADME-related genes. Genetic and clinical data were integrated using a machine learning–based approach to explore determinants of treatment response.

**Results:**

*CHST3* rs4148944 G/G genotype showed the strongest nominal association with PPI response (p < 0.0064). Integrative analysis revealed combinatorial patterns involving *CHST3, CHST11* gene (*rs7847*), *SULT1A2/A3* (*rs11859842*), *and NR1I3* (*rs55802895*), particularly in the presence of food allergy, which improved patient stratification. Although these additional variants were not individually significant, their combined occurrence—especially in association with food allergy—suggests a potential contribution of both metabolic and regulatory pathways to PPI response variability.

**Conclusion:**

Overall, this study provides exploratory, proof-of-concept evidence that the *CHST3* rs4148944 G/G genotype, together with other genetic variants and clinical features, may influence PPI response in EoE and contribute to patient stratification. These findings support the potential of integrative pharmacogenomic approaches, although validation in larger cohorts is required.

## Highlights

- Genetic variability in ADME-related pathways contributes to heterogeneous PPI response in pediatric EoE.
- *CHST3* rs4148944 is identified as a potential candidate pharmacogenomic marker associated with PPI response.
- Integrative analysis reveals combinatorial effects of *CHST3*, *SULT1A2/A3*, and *NR1I3* variants associated with food allergy.
- Machine learning supports patient stratification by integrating genetic and clinical features.
- Findings provide proof-of-concept for pharmacogenomic-driven precision medicine in EoE.

## 1 Introduction

Eosinophilic esophagitis (EoE) is a chronic or remitting immune-mediated disease, characterized by esophageal dysfunction and eosinophilic inflammation of the esophageal mucosa (Barni et al., 2021). Current guidelines define pediatric EoE as a clinicopathologic condition characterized by esophageal symptoms and ≥15 eosinophils per high-power field on biopsy, after exclusion of alternative causes (Amil Dias et al., 2024; Oliva et al., 2025), and emphasize a standardized diagnostic approach integrating clinical, endoscopic and histologic features, with specific considerations for pediatric patients (Dellon et al., 2026).

Over the past decades, the incidence and prevalence of EoE have increased (Barni et al., 2021), making it the leading cause of dysphagia in children and young adults.

EoE is a multifactorial disease involving genetic predisposition and environmental triggers, that contribute to epithelial barrier dysfunction and a type 2 helper T cell (Th2)-mediated immune response, ultimately leading to inflammation, remodeling, dysmotility, and fibrosis (Lyles and Rothenberg, 2019; Vinit et al., 2019).

Proton pump inhibitors (PPIs) are currently recognized as a treatment option in EoE rather than a diagnostic tool (Amil Dias et al., 2024), alongside topical corticosteroids and elimination diets (Barni et al., 2021; Amil Dias et al., 2024; Tamarit-Sebastian et al., 2022). Their widespread use as a potential first-line therapeutic option in EoE patients is supported by low cost, a favorable safety profile, ease of administration, and substantial rates of responsiveness (Dellon et al., 2018). Reported response rates in pediatric populations is ranged from 30% to 71%, with studies showing minimal clinical differences between PPI-responders (PPI-RE) and PPI-nonresponders (PPI-NORE) (Irastorza et al., 2022). On the other hand, long-term use of PPIs has been associated with certain adverse effects and interindividual variability in efficacy.

PPIs are primarily metabolized by cytochrome P450 enzymes, particularly CYP2C19 with additional contributions from CYP3A4/5 to their pharmacokinetic and pharmacodynamic profiles through hydroxylation and sulfoxidation processes. Genetic variability in *CYP2C19* significantly influences interindividual variability in PPI response. Loss-of-function alleles (*2, *3) reduce enzymatic activity and define poor or intermediate metabolizers, whereas the *17 allele increases activity and is associated with rapid or ultrarapid metabolism (Gawrońska-Szklarz et al., 2012; Rodríguez-Alcolado et al., 2024). Pharmacogenetic-guided dosing has been proposed for omeprazole, pantoprazole, and lansoprazole according to Clinical Pharmacogenetics Implementation Consortium guidelines (Lima et al., 2021).

Variants in the signal transducer and activator of transcription 6 (*STAT6*) have also been associated with reduced PPI efficacy in EoE, correlating with baseline eosinophil counts and endoscopic improvement, and disease activity and progression (Soria-Chacartegui et al., 2024).

Beyond these genes, additional Absorption, Distribution, Metabolism, and Excretion (ADME) genes, particularly *CYP3A5*, *ABCB1*, and a pharmacodynamic/drug-target gene, such as *ATP4A*, may contribute to interindividual variability in PPI response. Transcriptomic and biomarker studies have identified subtle differences between PPI-RE and PPI-NORE, but findings remain inconsistent and no validated predictors of responsiveness are currently available (Hamilton et al., 2022; Wen et al., 2015; Cañas et al., 2020).

Recent studies have highlighted a molecular heterogeneity among pediatric patients, suggesting that genetic and transcriptomic profiles may influence PPI responsiveness (Lucendo et al., 2025; Scodellaro et al., 2025; Bose et al., 2025).

As a result, identifying responders currently relies on an empirical trial of PPI followed by repeat endoscopy with biopsies to assess histological response (Muftah et al., 2024a). Therefore, a failed PPI trial may expose patients to unnecessary endoscopic procedures, prolonged symptoms, and increased risk of complications such as food impaction and fibrostenotic disease. Consequently, identifying reliable non-invasive or minimally invasive predictive markers of PPI response is essential for improving clinical outcomes and minimizing the burden of invasive procedures (Muftah et al., 2024a; Alexander et al., 2021; Muftah et al., 2024b).

Recent studies have explored blood-, saliva-, and stool-based biomarkers, as well as molecular signatures, with the potential to support diagnosis, monitor disease activity, and guide personalized treatment strategies (Marulo et al., 2025).

In this evolving landscape, pharmacogenomic approaches, integrating genetic and clinical data, represent a promising strategy to identify biomarkers of drug response. High-throughput platforms targeting ADME-related genes enable comprehensive evaluation of pharmacologically relevant variants (Arbitrio et al., 2016a), while artificial intelligence–based approaches are emerging as promising tools to integrate complex clinical, genetic, and molecular data supporting the identification of complex patterns underlying therapeutic variability.

In this way it will be possible to potentially improve disease phenotyping and treatment response prediction, although most applications still require validation before clinical implementation (Castagnaro et al., 2025).

The aim of this study was to investigate whether genetic variants in 231 ADME genes are associated with histologic and clinical response to PPI treatment in pediatric patients with EoE, and to identify potential pharmacogenomic biomarkers for patient stratification.

## 2 Materials and methods

This multicenter study included pediatric patients diagnosed with eosinophilic esophagitis at the Pediatrics Unit of Magna Graecia University (UMG), Catanzaro, and at the Gastroenterology and Nutrition, and Allergy Units of Bambino Gesù Children’s Hospital (OPBG), Rome. The study was conducted in accordance with the Declaration of Helsinki and approved by the local ethics committees (UMG: prot. n. 163/2019, 170/2021; OPBG: prot. n. 850/2020). Patients were eligible if they received PPI monotherapy, with no concomitant dietary elimination or topical corticosteroid therapy. Written informed consent was obtained from all participants and their legal guardians. Clinical data were retrospectively collected, including demographics, medical history, as well as allergic comorbidities. Given their potential influence on inflammatory phenotype and PPI responsiveness, these comorbidities were defined as follows.

Eczema was defined as a chronic inflammatory disease of the skin manifested by pruritus, lichenification, excoriation, and crusting, mainly on the flexural surfaces of the elbow and knee (Johansson et al., 2004).

Asthma was defined as an heterogeneous, chronic airway inflammatory disease defined by a history of variable respiratory symptoms—such as wheeze, shortness of breath, chest tightness, and cough—and variable expiratory airflow limitation. These symptoms usually fluctuate over time and intensity (Reddel et al., 2022).

Allergic rhinitis was defined as a symptomatic disorder of the nose induced by an IgE-mediated inflammation after allergen exposure of the membranes lining the nose (Bousquet et al., 2012).

The definition of IgE-mediated food allergy was a cutaneous, gastrointestinal (vomiting and/or diarrhea), respiratory, cardiovascular or generalized reaction occurring within 30 min of ingestion of foods for which there was a positive sensitization record (Arasi et al., 2023).

Following baseline endoscopy, all patients received twice-daily PPI therapy at a dose of 2 mg/kg/day for 8–12 weeks. In accordance with the institutional protocol, lansoprazole was the preferred first-line agent when not contraindicated. Upon completion of therapy, repeat upper gastrointestinal endoscopy with esophageal biopsies was performed to evaluate histological response.

Patients were classified as PPI-responders when both symptomatic improvement and histological remission were achieved. Clinical response was assessed using the Pediatric Eosinophilic Esophagitis Symptom Score (PEESS v2.0) (Aggoun Zioueche et al., 2026). A ≥30% reduction in the total PEESS score from baseline was considered a clinically relevant symptomatic response. Given the absence of a universally validated PEESS threshold for defining clinical remission, PEESS response was analyzed separately from histological response and was not used alone to classify patients as PPI responders. Histological disease activity was assessed by peak eosinophil count, and histological remission was defined as fewer than 15 eosinophils per high-power field. Both criteria had to be met concomitantly; patients failing either were classified as PPI-NORE.

### 2.1 Sample collection and genotyping

For each subject, genomic DNA was extracted from peripheral whole blood using QIAamp DNA Blood Mini Kit (Qiagen, Valencia, CA, United States), in accordance with the manufacturer’s instructions. DMET Plus assay (ThermoFisher Scientific, Waltham, MA, United States) was performed as previously described (Giannitrap et al., 2024; Scionti et al., 2017; Di Martino et al., 2016; Arbitrio et al., 2021; Scionti et al., 2022; Arbitrio et al., 2019). In this hypothesis-generating study we used DMET Plus microarray platform to genotype 58 EoE pediatric patients for 1931 SNPs and 5 copy number variations across 231 genes involved in drug ADME and pharmacokinetic variability with the aim of identifying novel potential predictive biomarkers of PPI response and clinical outcome (Arbitrio et al., 2016a; Giannitrap et al., 2024; Scionti et al., 2017; Di Martino et al., 2016; Arbitrio et al., 2021).

Genotyping and all genetic analyses were conducted at the Institute for Biomedical Research and Innovation (IRIB), National Research Council (CNR) in Catanzaro.

### 2.2 Statistical analysis

Data were analyzed, using appropriate statistical methods, at the Institute of Clinical Physiology (IFC-CNR) in Reggio Calabria. All statistical analyses were performed using Stata version 16 (StataCorp LLC, College Station, TX, United States). Continuous variables were expressed as mean and standard deviation (SD), while categorical variables were reported as absolute frequencies and percentages. The distribution of all continuous variables was assessed before performing the descriptive analyses. The distributions were found to be approximately normal and for this reason continuous variables were appropriately summarized as mean ± standard deviation (SD). Differences between PPI-RE and PPI-NORE groups were evaluated using logistic regression analysis. Both univariable and multivariable models were constructed to identify potential predictors of PPI response. Prior to multivariable logistic regression analysis, multicollinearity among the independent variables was assessed using the variance inflation factor (VIF). All variables exhibited VIF values below 5, indicating no significant multicollinearity and supporting the stability, robustness, and interpretability of the multivariable model. Variables that were significantly associated with response status in univariable analysis (p < 0.10), were included in the multivariable model**.** Genotype distributions between PPI RE and NO_RE were compared using two-sided Fisher’s exact tests for 2 × 2 tables and Fisher–Freeman–Halton exact tests for genotype distributions with more than two categories. Univariable and multivariable associations with PPI response were evaluated using Firth’s bias-reduced penalized logistic regression because of the limited sample size, sparse genotype categories, and complete separation observed for *CHST3* rs4148944. Multicollinearity among predictors included in the multivariable model was assessed using the VIF. Moreover, we applied classification tree to identify interactions among several predictive SNPs and genotypes. We associated the ADME related genes to laboratory and clinical parameters in order to explore potential associations with clinical conditions that may influence outcomes in terms of PPI response. The preliminary overall discriminatory power of identified SNPs and genotypes as predictive and prognostic factors was tested through the Receiver Operating Characteristic (ROC) curves and the confusion matrix to support the power of performance of the ML approach also in terms of a model’s correct and incorrect predictions for PPI RE/NORE class. In addition, to assess the global distribution of genotype-association p-values and identify potential systematic deviations from the expected null distribution, a quantile–quantile (QQ) plot of the observed versus expected p-values was generated. The QQ plot is reported in Supplementary Material S2 as Supplementary Figure S1.

### 2.3 Pharmacogenomic analysis

Allele frequency of all 1,931 SNP markers and the 5 copy-number variants (CNVs) were analyzed using DMET-Analyzer statistical software. Differences between groups were assessed using Fisher’s exact test with FDR correction applied for multiple comparisons, based on the number of alleles tested. Adjusted p-values <0.05 were considered statistically significant (Guzzi et al., 2012). ADME genotyping was performed using DMET Console v1.1 (Thermo Fisher Scientific). Variant calling and primary marker annotation were performed using DMET Console software and the corresponding DMET Plus annotation library. Probe-specific annotations, including gene assignment, dbSNP identifier, genomic position, nucleotide change and, where applicable, star-allele information, were derived directly from the DMET Console output. For variants retained in the final predictive model, these historical platform annotations were additionally cross-checked against current genomic reference databases (Supplementary Table S6). All variants were tested for Hardy–Weinberg equilibrium and all variants with a call rate ≤96%, high “possible rare allele”/“no call” rates, or <100% concordance were excluded from analysis. The analytical performance and concordance of the DMET platform was previously validated with alternative orthogonal methods, such as TaqMan assays and direct sequencing (Arbitrio et al., 2016a; Giannitrap et al., 2024; Scionti et al., 2017; Di Martino et al., 2016; Arbitrio et al., 2016b). Therefore, no additional technical validation was performed. After quality-control filtering, 1,585 of the 1,936 initially assessed variants were excluded because they were non-informative, showed complete genotype concordance across the study population, or had high rates of “possible rare allele,” “no call,” or “zero copy number” classifications. Hardy–Weinberg equilibrium was assessed for the 351 informative markers retained after initial quality-control filtering. Because HWE was assessed across multiple markers, FDR correction was applied, leading to the exclusion of three variants that remained significant after adjustment.

Therefore, 348 markers were included in the final association analysis. The final dataset therefore comprised 348 genetic markers distributed across 231 genes, which were included in the association analysis and used as the basis for the Bonferroni correction (threshold of *p* < 1.44 × 10^−4^) (Figure 1).

**FIGURE 1:**
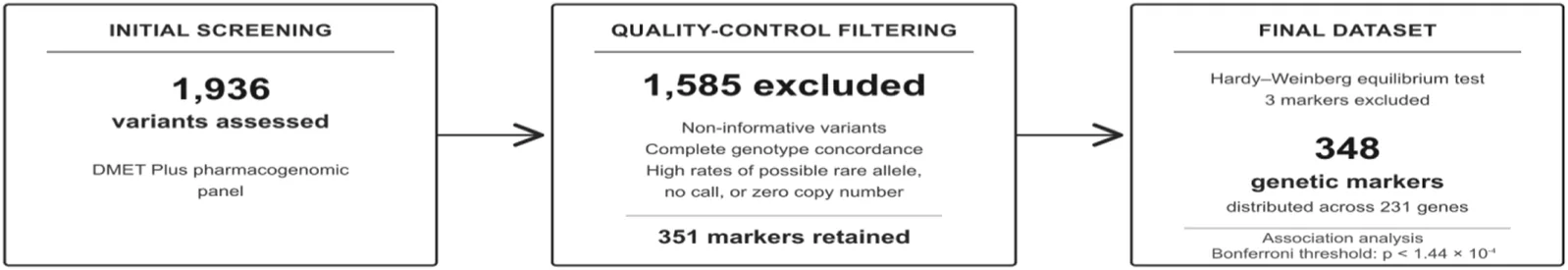
The overall flow diagram filtering process.

All the statistical test provided from DMET-Analyzer are available as Tables 1, 2 in the Supplementary File S2.

**TABLE 1:** Demographic, clinical, laboratory and allergic features of all enrolled patients.

​	​	PPI response		​
	Whole sample (n = 58)	No responders (n = 41)	Responders (n = 17)	P value
Demographic				
Age (years)	15.4 ± 3.9	15.5 ± 4.1	15.0 ± 3.4	0.60
Age at diagnosis (years)	11.9 ± 3.6	12.2 ± 3.3	11.2 ± 4.1	0.41
Female sex, n. (%)	6 (10.3)	4 (9.8)	2 (11.8)	1.00
Symptoms at diagnosis				
Regurgitation, n. (%)	6 (10.3)	2 (4.9)	4 (23.5)	0.06
Vomit, n. (%)	14 (24.1)	10 (24.4)	4 (23.5)	1.00
Dysphagia with food impaction, n. (%)	31 (53.4)	23 (56.1)	8 (47.1)	0.57
Dysphagia without food impaction, n. (%)	12 (20.7)	8 (19.5)	4 (23.5)	0.73
Chest pain, n. (%)	6 (10.3)	4 (9.8)	2 (11.8)	1.00
Heartburn, n. (%)	9 (15.5)	6 (14.6)	3 (17.6)	1.00
Epigastric pain, n. (%)	11 (19.0)	6 (14.6)	5 (29.4)	0.27
Allergic comorbidities				
Asthma, n. (%)	15 (25.9)	12 (29.3)	3 (17.6)	0.51
Rhinitis, n. (%)	45 (77.6)	32 (78.0)	13 (76.5)	1.00
Dermatitis, n. (%)	12 (20.7)	8 (19.5)	4 (23.5)	0.73
Allergy to foods, n. (%)	13 (22.4)	12 (29.3)	1 (5.9)	0.08
Number of comorbidities, n. (%) 0 1 2 3	8 (13.8) 28 (48.3) 21 (36.2) 1 (1.7)	4 (9.8) 20 (48.8) 16 (39.0) 1 (2.4)	4 (23.5) 8 (47.1) 5 (29.4) 0 (0.0)	0.49

Continuous variables were compared using the t-test, while categorical variables were compared using Fisher’s exact test. The distribution of the number of llergic comorbidities was compared using an exact test for contingency tables (Fisher-Freeman-Halton).

**TABLE 2:** Distribution of PPI treatment and total daily dose in our cohort.

PPI	Number of patients	Mean dose per administration*	Administration frequency	Mean total daily dose*
Lansoprazole	30	30 mg	Twice daily	60 mg/day
Lansoprazole	9•	1 mg/kg/dose	Twice daily	45/mg/day
Pantoprazole	10	40 mg	Twice daily	80 mg/day
Omeprazole	4	40 mg	Twice daily	80 mg/day
Omeprazole	1°	20 mg	Twice daily	40 mg/day
Esomeprazole	4	20 mg	Twice daily	40 mg/day

* All patients received double-dose PPI therapy, up to a maximum of 2 mg/kg/day according.

to protocol.

• Nine patients had a weight <30 Kg.

° One patient had a weight <40 Kg.

To identify potential associations between the presence or absence of specific genetic polymorphisms and clinical conditions that may influence EoE outcomes, we integrated DMET datasets with corresponding clinical information. Genomic and clinical datasets were merged using an inner join on a unique patient identifier. As a result, only patients with a matching identifier in both datasets were retained in the final cohort, and records present in only one dataset were excluded. This procedure ensured that each included patient had both genomic and clinical data and prevented the introduction of additional missing values from unmatched records.

### 2.4 Classification rules

EOE is a complex disease whose progression or regression results from interactions among multiple factors and genes. Because conventional statistical tests are unable to detect such multi-gene interactions, it is necessary to employ analytical methods that can evaluate multiple genes simultaneously to reveal hidden interactions. To overcome the limitations related to statistical tests, we developed a machine learning predictive model using Python (version 3.9) and the scikit-learn library (version 1.5.2). The Python code of the presented pipeline is reported in Supplementary 2 Code1. The workflow comprised the following steps:

1. Encoding of categorical variables: Categorical features, including genotype data and relevant clinical variables, were encoded using one-hot encoding, as genotype categories were treated as nominal rather than ordinal variables. This approach ensured compatibility with the machine learning algorithms while preserving the distinct categorical information without imposing an artificial ordering among categories.
2. Handling of missing values: Missing data were imputed using the most frequent value within each variable, implemented via scikit-learn’s Simple Imputer (strategy = “most frequent”). This approach was selected to minimize bias while maintaining the statistical reliability of the dataset.
3. Feature selection: Dimensionality reduction was performed to identify the most informative features, thereby enhancing model interpretability and mitigating the risk of overfitting. Feature selection was informed by domain expertise in conjunction with scikit-learn’s feature selection utilities.
4. Input dataset oversampling: The dataset consisted of 58 samples in total. It was first divided into a training set of 46 samples and an independent test set of 12 samples. Before oversampling, the training set contained 33 NORE cases and 13 RE cases, thus showing a clear class imbalance. To address this issue, oversampling was applied exclusively to the training partition using Random Over Sampler, which randomly replicates instances from the minority class until the classes are balanced. In this case, 20 additional minority-class samples were generated, increasing the size of the training set from 46 to 66 samples. The test set was intentionally left unchanged, remaining 12 samples both before and after oversampling, in order to avoid information leakage and preserve an unbiased estimate of model performance. A decision tree classifier was trained using scikit-learn’s Decision Tree Classifier, with the random state parameter set to 42 to ensure reproducibility. Although decision-tree algorithms can theoretically handle mixed data types, this scikit-learn implementation requires numerical input and does not natively support categorical variables. Therefore, to overcome these issues, all the relevant variables, were encoded using one-hot encoding. This approach ensured compatibility with the machine learning algorithms while preserving the distinct categorical information without imposing an artificial ordering among categories.
5. Model training: A decision tree classifier was trained using scikit-learn’s Decision Tree Classifier, with the random_state parameter set to 42 to ensure reproducibility and consistent model performance.

### 2.5 Graphical workflow

To provide a unified representation of the study design and analytical strategy, the complete clinical, pharmacogenomic, and machine-learning methodology is summarized in Figure 2.

**FIGURE 2:**
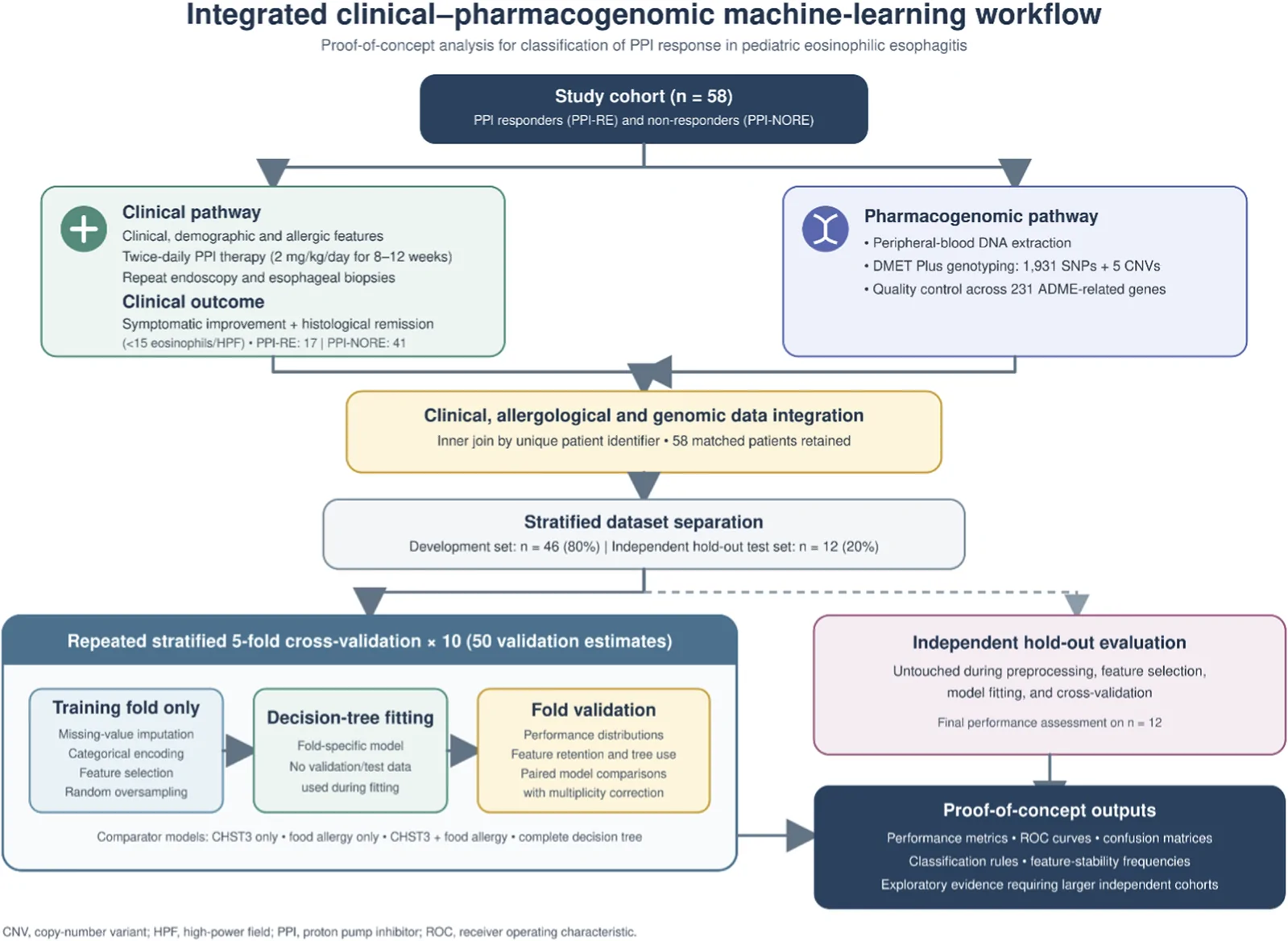
Overview of the Integrated pharmacogenomic and machine-learning workflow.

The workflow begins with the enrolment of 58 pediatric patients with eosinophilic esophagitis and follows two complementary pathways. The clinical pathway includes collecting demographic, clinical, and allergy-related variables, administering PPI therapy at 2 mg/kg/day for 8–12 weeks, and assessing response by repeat endoscopy and esophageal biopsies. In parallel, the pharmacogenomic pathway comprises peripheral-blood DNA extraction and DMET Plus microarray genotyping.

Following the DMET-Analyzer statistical analysis, the relevant 351 probes and clinical data were integrated through an inner join based on the unique patient identifier, retaining the 58 patients represented in both datasets. The dataset was stratified into a development set of 46 patients and an independent hold-out test set of 12 patients. Within the development set, model robustness was assessed using repeated stratified five-fold cross-validation with 10 repetitions. A decision-tree classifier was subsequently trained to distinguish PPI responders from non-responders. The final steps of the workflow comprise cross-validated performance assessment, feature-stability analysis, evaluation on the independent test set, and generation of classification rules, performance metrics, ROC curves, and confusion matrices.

## 3 Results

A total of 58 EoE pediatric patients (52 males; mean age 15.4 ± 3.9 years) were included in the study. Baseline demographic, clinical, and allergic comorbidities of the cohort, including PPI response subgroups, are summarized in Table 1. No significant differences were observed between PPI responders (PPI-RE, n = 17) and non-responders (PPI-NORE, n = 41) in terms of age, sex, symptoms, or comorbidities.

All 58 patients received double-dose PPI therapy, up to a maximum of 2 mg/kg/day or, according to the hospital protocol, up to 30 mg of Lansoprazole twice daily (n = 39). A minority of patients received alternative PPIs, including Pantoprazole up to 40 mg twice daily (n = 10), Omeprazole up to 40 mg twice daily (n = 5), or Esomeprazole up to 20 mg twice daily (n = 4). Distribution of PPI treatment and total daily dose in our cohort are reported in Table 2.

The median duration of therapy was 12 weeks (range: 8–12 weeks). The 29.3% PPI response rate observed in our cohort lies at the lower end of the 30%–71% range previously reported in pediatric EoE. Of the enrolled patients, 17 (29.3%) were PPI-RE and 41 (70.7%) were PPI-NORE. Of the 41 non-responders, 27 received lansoprazole, 6 pantoprazole, 3 esomeprazole, and 5 omeprazole, with no significant differences in PPI type distribution nor in treatment duration compared to PPI-RE patients.

The most frequent symptoms were dysphagia, with food impaction (53.4%) and without food impaction (20.7%), followed by vomiting (24.1%) and epigastric pain (19%).

Atopic comorbidities were common, including allergic rhinitis/rhinoconjunctivitis (77.6%), asthma (25.9%), eczema (20.7%), and IgE-mediated food allergy (22.4%).

However, no demographic or clinical variables—such as symptoms, presence of specific comorbidities—were significantly associated with PPI response status in either univariate or multivariate analyses.

Regarding EoE phenotypes, only 3/58 patients presented with strictures at endoscopy (all belonging to the PPI-NORE group), while 10/58 had esophageal rings; 3 out of these 10 patients were PPI responders. Therefore, among the 41 PPI-NORE, only 10 patients presented with a fibrostenotic phenotype. The overall distribution of genotype-association p-values was examined using a QQ plot, which is reported in Supplementary Material S2 (Supplementary Figure S1). This analysis provided a global assessment of the observed association p-value distribution. Among the genomic markers investigated in the 58-patient cohort, *CHST3* rs4148944 showed the strongest nominal association with PPI response. The G/G genotype was observed in all PPI responders (17/17, 100%) and in 28 of 41 non-responders (68.3%), whereas alternative genotypes were observed exclusively among non-responders (13/41, 31.7%). The verified genotype distribution was 45 G/G, 9 A/G, and 4 A/A. A two-sided Fisher’s exact test yielded an unadjusted p-value of 0.006433. rs4148944 showed a nominal deviation from HWE (exact p = 0.0122), mainly due to a deficit of heterozygotes; however, this deviation did not remain significant after FDR correction across the 351 markers tested for HWE. Similarly, the association with PPI response did not meet the pre-specified significance threshold after multiple-testing correction (FDR-adjusted q = 0.0643; Table 3), and therefore none of the genomic associations was considered statistically significant after correction. Nevertheless, rs4148944 remained the most stable and discriminative genomic feature in the predictive analysis: the corresponding probe was retained in all 50 repeated cross-validation fits and was selected as a splitting variable in 46/50 fitted decision trees (92%). Because of the limited cohort size and the absence of an external validation cohort, the reported cross-validated performance should not be interpreted as evidence of clinical predictive validity. Complete univariable and multivariable Firth penalized logistic regression results are reported in Supplementary Table S7, including the corrsponding odds ratios, 95% confidence intervals, and p-values for *CHST3* rs4148944, *CHST11* rs7847, *SULT1A2/A3* rs11859842, *NR1I3* rs55802895, and IgE-mediated food allergy. This consistency across repeated resampling supports *CHST3* rs4148944 as the strongest exploratory genomic signal identified in this cohort, while not establishing it as an FDR-confirmed or independently validated biomarker.

**TABLE 3:** CHST3 gene (rs4148944) genotype distribution.

Genotype	PPI-RE	PPI-NORE	Total
GG	17/17 (100%)	28/41 (68.3%)	45 (77.6%)
AG	0 (0%)	9/41 (21.9%)	9 (15.5%)
AA	0 (0%)	4/41 (9.8%)	4 (6.9%)

No *CYP2C19* variant was significantly associated with PPI response, as genotype distributions were comparable between responders and non-responders, with odds ratios close to 1 and confidence intervals including 1 (Supplementary Table S1). *CYP2C19* diplotypes were additionally translated into predicted metabolizer phenotypes according to current CPIC criteria (Supplementary Table S2). Among PPI responders, 5/17 (29.4%) were rapid metabolizers and none were ultrarapid metabolizers, whereas among non-responders 5/41 (12.2%) were rapid metabolizers and 1/41 (2.4%) was an ultrarapid metabolizer. No *CYP2C19* metabolizer phenotype was significantly associated with PPI response in two-sided Fisher’s exact analyses. In an exploratory secondary analysis combining rapid and ultrarapid metabolizers, RM + UM phenotypes were observed in 5/17 (29.4%) responders and 6/41 (14.6%) non-responders, with no significant difference between groups (two-sided Fisher’s exact p = 0.2704; Supplementary Table S3).

Variants interrogated within *CYP3A5, ABCB1* and *ATP4A* did not show significant associations with PPI response and none of these genes was retained as a splitting feature or included in the final decision-tree-derived association rules. Instead, *STAT6* is not represented on the DMET Plus platform and could therefore not be evaluated. None of the five CNVs showed a significant association with PPI response; therefore, they were not retained for subsequent association, predictive, or decision-tree analyses.

During the mining phase, the classification model applied to DMET dataset analyzed in correlation to clinical and laboratory features generated a decision tree presented in Figure 3.

**FIGURE 3:**
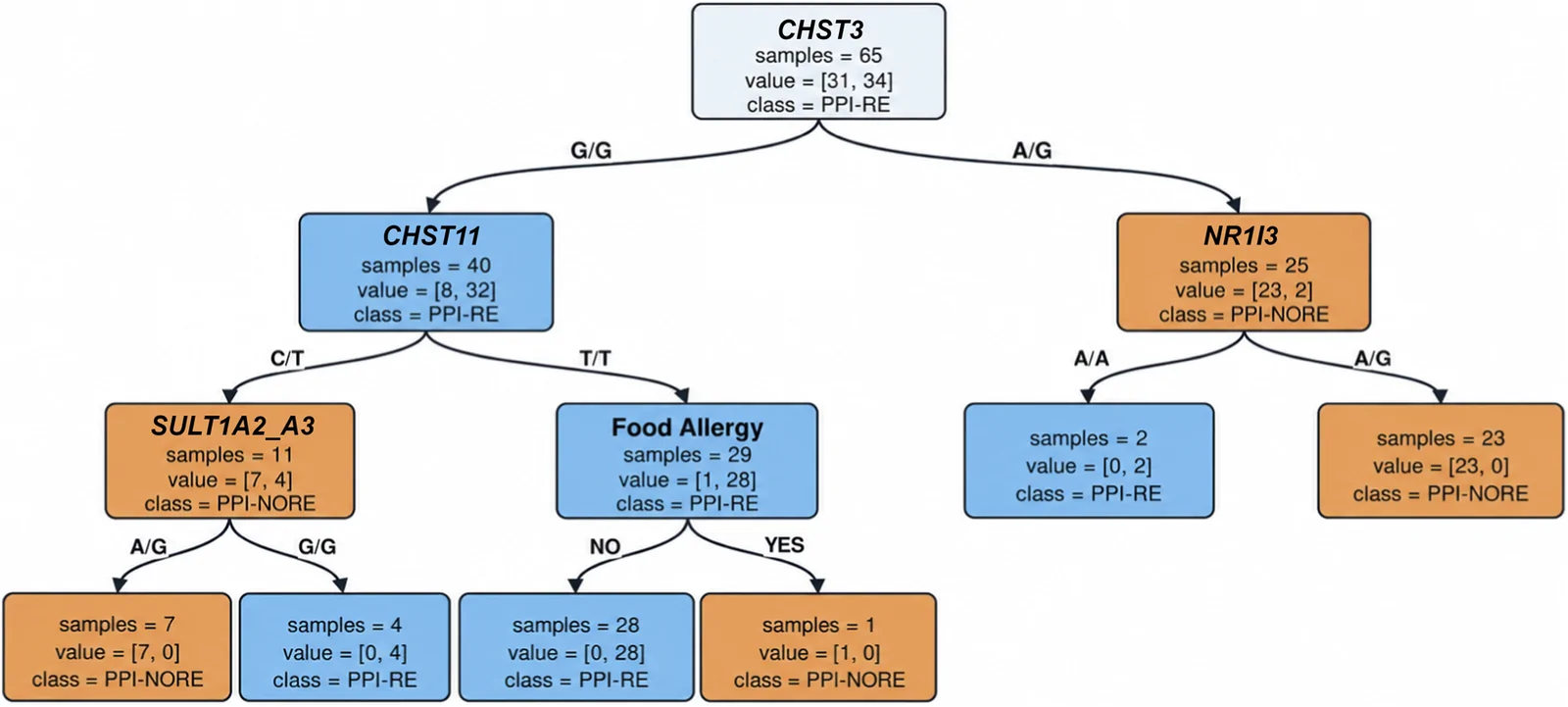
Decision tree generated for the EoE study after application of RandomOverSampler to the training data. The decision tree illustrates the classification process used to distinguish PPI responders (PPI-RE) from PPI non-responders (PPI-NORE) based on selected genetic polymorphisms and clinical characteristics. Each internal node represents a decision based on a selected feature, whereas each terminal node (leaf) indicates the predicted class. Each path from the root node to a terminal node defines a classification rule. The genetic variants included in the tree are located in or associated with *CHST3* (rs4148944), *CHST11* (rs7847), *SULT1A2/A3* (rs11859842), and *NR1I3* (rs55802895).

The resulting decision tree achieved an accuracy of 0.8333 on the independent test set, with precision, recall, and F1-score all equal to 0.7500. The results of these correlations highlight the role of the G/G genotype in *CHST3* gene (rs4148944) as significantly associated, together to the homozygous TT genotype (rs7847) in the *CHST11* gene of the same family, to food allergy whose positive correlation could allow to stratify no responder’s patients contrary to absence of food allergy which could characterize the class of responder’s patients. The other polymorphic variants identiwed in the association rules—rs11859842, *SULT1A2_A3* and the rs55802895 *NR1I3* were not statistically signiwcant after Fisher’s test but can contribute to patient stratiwcation when considered in combination with G/G genotype in *CHST3* gene (rs4148944), as reported in Supplementary Table S3. Updated genomic annotation of the variants retained in the final decision-tree/rule-based analysis is provided in Supplementary Table S6, together with the corresponding original DMET Console gene assignments.

The conversion of the decision tree in Figure 3 yielded six classification rules, outlined as follows:

IF *CHST3* = G/G AND *CHST11* = C/T AND *SULT1A2_A3* = A/G, then PPI-NORE.

IF *CHST3* = G/G AND *CHST11* = C/T AND *SULT1A2_A3* = G/G, then PPI-RE.

IF *CHST3* = G/G AND *CHST11* = T/T AND food allergy = No, then PPI-RE.

IF *CHST3* = G/G AND *CHST11* = T/T AND food allergy = Yes then PPI-NORE.

IF *CHST3* = G/G AND *NR1I3* = A/A then PPI-RE.

IF *CHST3* = A/G AND *NR1I3* = A/G then PPI-NORE.

The two complementary approaches used to further assess the decision tree’s performance were the ROC curve and the confusion matrix. The corresponding Area Under the Curve (AUC = 0.7812), shown in Figure 4, suggests that the classifier has promising but still potential ability to distinguish between PPI-RE and PPI-NORE. The confusion matrix further showed that 10 out of 12 subjects were correctly classified, with 7/8 NORE and 3/4 RE cases correctly identified.

**FIGURE 4:**
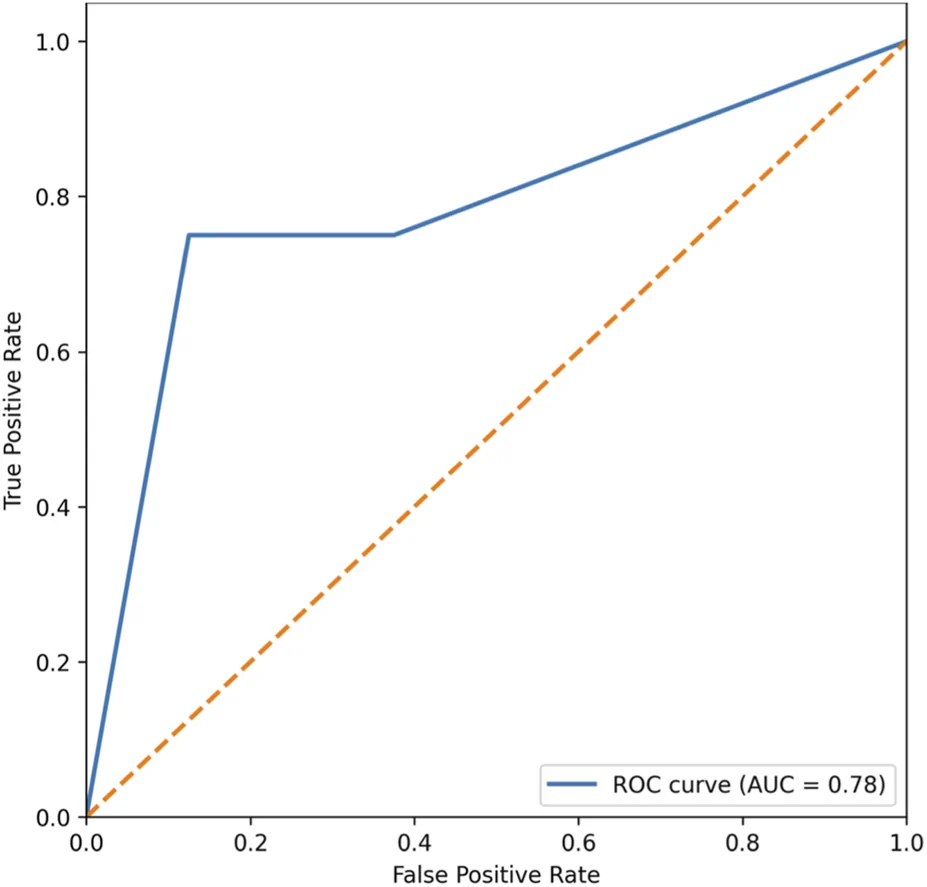
Receiver Operating Characteristic (ROC) curve for the decision tree classifier. The curve illustrates the model’s ability to discriminate between responders and non-responders, with an early elevation indicating high sensitivity at conservative thresholds.

The confusion matrix provides a detailed summary of the classification outcomes obtained on the independent test set, which comprised 12 samples, corresponding to 20.7% of the overall dataset (N = 58). The confusion matrix is shown in Figure 5.

**FIGURE 5:**
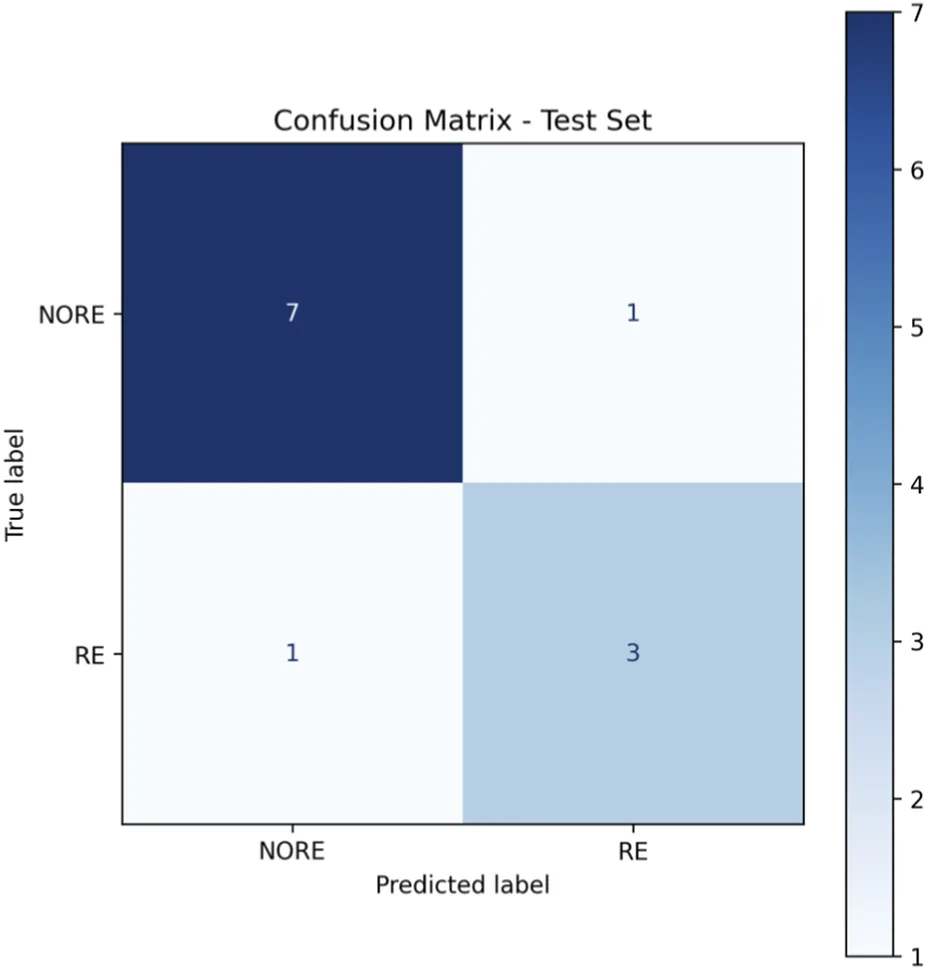
Confusion matrix of the decision tree classifier. The matrix shows the classification outcomes for 12 cases: 7 true negatives (non-responders correctly classified), 3 true positives (responders correctly classified), 1 false negative (a responder misclassified as a non-responder), and 1 false positive (a non-responders misclassified as responders). These results reflect an overall accuracy of 83%.

### 3.1 Performance metrics of the decision tree

To develop and evaluate the proposed ML approach, the dataset comprising 58 patients was stratified into a training set (n = 46; 80%) and an independent test set (n = 12; 20%). Classifier performance was assessed using accuracy, balanced accuracy, precision, recall, F1-score, and the area under the receiver operating characteristic curve (ROC-AUC).

To determine whether the complete model provided predictive information beyond *CHST3* rs4148944 and food allergy, four decision-tree models were compared: *CHST3* rs4148944 alone, food allergy alone, *CHST3* rs4148944 combined with food allergy, and the complete candidate-feature model. All models were evaluated using the same repeated stratified five-fold cross-validation partitions with 10 repetitions. Performance was assessed using accuracy, balanced accuracy, precision, recall, F1-score, and ROC-AUC. For each repetition, the fold-level scores were averaged, and paired differences between the complete and reduced models were evaluated using the Wilcoxon signed-rank test. Holm correction was applied separately to the three pairwise model comparisons for each performance metric. The models were also evaluated descriptively on the independent hold-out test set. Table 4 reports their performance in repeated stratified cross-validation.

**TABLE 4:** Performance of the complete and reduced decision-tree models evaluated using repeated stratified five-fold cross-validation with 10 repetitions.

Model	Metric	Mean	SD	ci95 l	ci95 u
*CHST3* only	Accuracy	0.802	0.06	0.78302633	0.82097367
*CHST3* only	Balanced accuracy	0.802	0.059	0.78334256	0.82065744
*CHST3* only	Precision	0.733	0.078	0.70833423	0.75766577
*CHST3* only	Recall	0.708	0.062	0.68839388	0.72760612
*CHST3* only	F1-score	0.674	0.052	0.65755616	0.69044384
CHST3 only	ROC-AUC	0.762	0.058	0.74365879	0.78034121
Food allergy only	Accuracy	0.546	0.088	0.51817196	0.57382804
Food allergy only	Balanced accuracy	0.546	0.086	0.51880441	0.57319559
Food allergy only	Precision	0.484	0.063	0.46407765	0.50392235
Food allergy only	Recall	0.685	0.086	0.65780441	0.71219559
Food allergy only	F1-score	0.501	0.064	0.48076142	0.52123858
Food allergy only	ROC-AUC	0.503	0.086	0.47580441	0.53019559
*CHST3* + food allergy	Accuracy	0.687	0.167	0.63418996	0.73981004
*CHST3* + food allergy	Balanced accuracy	0.687	0.166	0.63450619	0.73949381
*CHST3* + food allergy	Precision	0.629	0.154	0.58030092	0.67769908
*CHST3* + food allergy	Recall	0.677	0.078	0.65233423	0.70166577
*CHST3* + food allergy	F1-score	0.594	0.118	0.55668512	0.63131488
*CHST3* + food allergy	ROC-AUC	0.647	0.168	0.59387374	0.70012626
Full decision tree	Accuracy	0.824	0.079	0.79901801	0.84898199
Full decision tree	Balanced accuracy	0.824	0.079	0.79901801	0.84898199
Full decision tree	Precision	0.766	0.096	0.73564213	0.79635787
Full decision tree	Recall	0.712	0.089	0.68385573	0.74014427
Full decision tree	F1-score	0.693	0.075	0.66928292	0.71671708
Full decision tree	ROC-AUC	0.781	0.079	0.75601801	0.80598199

The complete decision-tree model achieved the highest mean performance across all the evaluated metrics, with an accuracy of 0.824 ± 0.079, balanced accuracy of 0.824 ± 0.079, precision of 0.766 ± 0.096, recall of 0.712 ± 0.089, F1-score of 0.693 ± 0.075, and ROC-AUC of 0.781 ± 0.079.

The *CHST3*-only model showed the closest performance to the complete model, achieving an accuracy of 0.802 ± 0.060, balanced accuracy of 0.802 ± 0.059, precision of 0.733 ± 0.078, recall of 0.708 ± 0.062, F1-score of 0.674 ± 0.052, and ROC-AUC of 0.762 ± 0.058. By comparison, the food-allergy-only model yielded an accuracy of 0.546 ± 0.088, balanced accuracy of 0.546 ± 0.086, precision of 0.484 ± 0.063, recall of 0.685 ± 0.086, F1-score of 0.501 ± 0.064, and ROC-AUC of 0.503 ± 0.086. The model combining *CHST3* and food allergy achieved an accuracy of 0.687 ± 0.167, balanced accuracy of 0.687 ± 0.166, precision of 0.629 ± 0.154, recall of 0.677 ± 0.078, F1-score of 0.594 ± 0.118, and ROC-AUC of 0.647 ± 0.168.

Compared with the *CHST3*-only model, the complete model showed modest improvements in ROC-AUC (mean paired difference: 0.019; 95% CI: 0.003–0.035), balanced accuracy (0.022; 95% CI: 0.007–0.037), and F1-score (0.019; 95% CI: 0.004–0.033). These differences remained statistically significant after Holm correction (adjusted p = 0.006 for each comparison). The complete model also showed higher mean performance in both the food-allergy-only model and the model combining *CHST3* and food allergy across all evaluated performance metrics. In particular, the marked reduction in ROC-AUC observed for the food-allergy-only model indicates that food allergy alone provided limited discriminatory information. Furthermore, adding food allergy to *CHST3* did not improve performance over the *CHST3*-only model. Overall, these findings indicate that CHST3 provided most of the predictive signal captured by the reduced models, whereas the additional candidate features included in the complete model produced a modest but consistent improvement in overall predictive performance. The distributions of the evaluation metrics across the 10 repetitions are presented in Figure 6.

**FIGURE 6:**
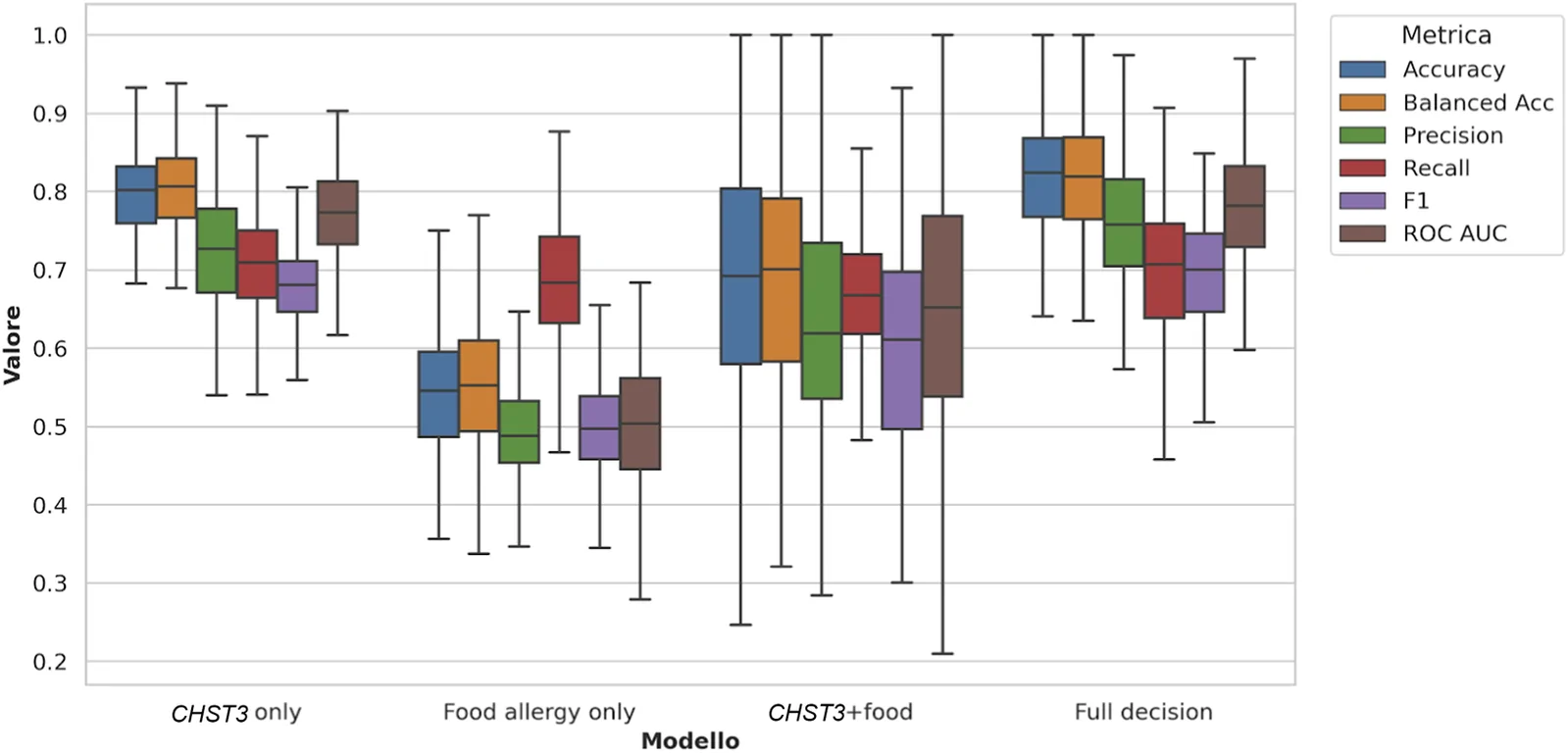
Box plot distribution of performance metrics across the four evaluated models (*CHST3* only, Food allergy only, *CHST3*+food allergy, and Full decision).

Given the limited cohort size, model robustness was assessed on the development set using repeated stratified five-fold cross-validation with 10 repetitions, yielding 50 validation-fold estimates. All data-dependent procedures were performed independently within each cross-validation iteration. Specifically, missing-value imputation, categorical-variable encoding, feature selection, and random oversampling were fitted exclusively using the training portion of each fold and subsequently applied to the corresponding validation portion. Random oversampling was restricted to the training folds. The independent test set was not used during preprocessing, feature selection, model fitting, or cross-validation.

Across the repeated cross-validation analysis, the complete classifier achieved a mean accuracy of 0.824 ± 0.079, balanced accuracy of 0.824 ± 0.079, precision of 0.766 ± 0.096, recall of 0.712 ± 0.089, F1-score of 0.693 ± 0.075, and ROC-AUC of 0.781 ± 0.079. The corresponding 95% CIs were 0.799–0.849 for accuracy, 0.799–0.849 for balanced accuracy, 0.736–0.796 for precision, 0.684–0.740 for recall, 0.669–0.717 for F1-score, and 0.756–0.806 for ROC-AUC. The variability observed across the performance estimates likely reflects the limited sample size and the small number of PPI responders included in the individual validation folds.

Following repeated cross-validation, the complete pipeline was fitted to the entire training set and evaluated once on the untouched independent test set comprising 12 patients. The classifier correctly classified 10 of the 12 patients, achieving an accuracy of 0.833, precision of 0.750, recall of 0.750, F1-score of 0.750, and ROC-AUC of 0.781. Given the limited size of the independent test set, these results should be interpreted as exploratory and not as definitive evidence of clinical generalizability. The repeated cross-validation and independent test-set results are summarized in Table 5.

**TABLE 5:** Predictive performance of the complete classifier in repeated stratified cross-validation and on the independent test set.

Metric	Repeated CV, mean ± SD	95% CI	Independent test
Accuracy	0.824 ± 0.079	0.799–0.849	0.833
Precision	0.766 ± 0.096	0.736–0.796	0.750
Recall	0.712 ± 0.089	0.684–0.740	0.750
F1-score	0.693 ± 0.075	0.669–0.717	0.750
ROC-AUC	0.781 ± 0.079	0.756–0.806	0.781

The repeated stratified cross-validation analysis also showed variability in predictive performance across the resampling iterations, as expected given the limited sample size. Accuracy and ROC-AUC exhibited comparatively narrower distributions, whereas precision, recall, and F1-score showed greater variability across the validation folds. The distributions of the performance metrics across the 50 validation folds are presented in Figure 7.

**FIGURE 7:**
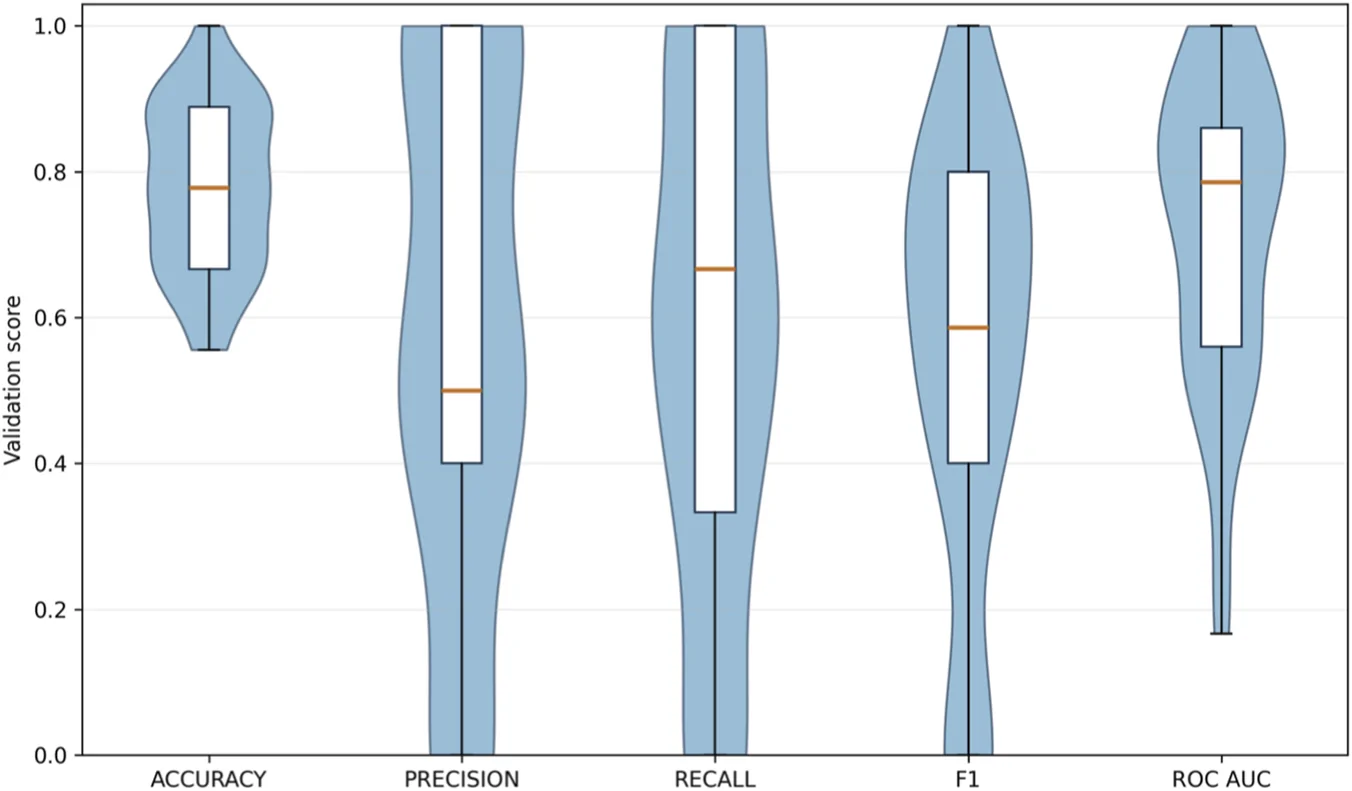
Distribution of classifier performance across the repeated cross-validation folds. Violin plots show the distributions of accuracy, precision, recall, F1-score, and ROC-AUC across the 50 validation folds generated using repeated stratified five-fold cross-validation with 10 repetitions.

Feature stability was assessed across the 50 repeated cross-validation fits by recording both the predictors retained by the fold-specific feature-selection procedure and those subsequently used as splitting variables by the fitted decision trees. The analysis included the *CHST3*-associated probes AM_10004 (rs4148944) and AM_10010 (rs4148949), the *CHST11*-associated probe AM_(rs7847)), the *NR1I3*-associated probes AM_11834 (rs55802895) and AM_11835 (rs72549319), and the *SULT1A2/ A3*-associated probes AM_11005 (rs9923231), AM_11022 (rs11859842), and AM_11023 (rs11150564), together with food allergy as the clinical predictor.

At the gene or clinical-variable level, *CHST3*, *CHST11, SULT1A2/A3*, and *NR1I3* were retained by the feature-selection procedure in all 50 wts (100%), whereas food allergy was retained in 36 wts (72%). *CHST3* showed the greatest structural stability: at least one of its associated probes, AM_10004 or AM_10010, was used as a splitting variable in all 50 wtted trees (100%). The *CHST11*- associated probe AM_10579 (rs7847) contributed to the tree structure in 32 wts (64%), while at least one of the *NR1I3*-associated probes, AM_11834 or AM_11835, was used in 30 wts (60%). The *SULT1A2/A3*-associated probes AM_11005, AM_11022, and AM_11023 were collectively used in four fits (8%), whereas food allergy was used as a splitting variable in five fits (10%). Thus, all five investigated predictors contributed to the structure of at least one fitted decision tree, although their frequencies of use differed substantially. Frequent retention by the feature-selection procedure did not necessarily translate into equally frequent structural use by the classifier. Overall, *CHST3* emerged as the most reproducible predictor across the resampling iterations, followed by *CHST11* and *NR1I3*, whereas *SULT1A2/A3* and food allergy provided less frequent structural contributions Figure 8.

**FIGURE 8:**
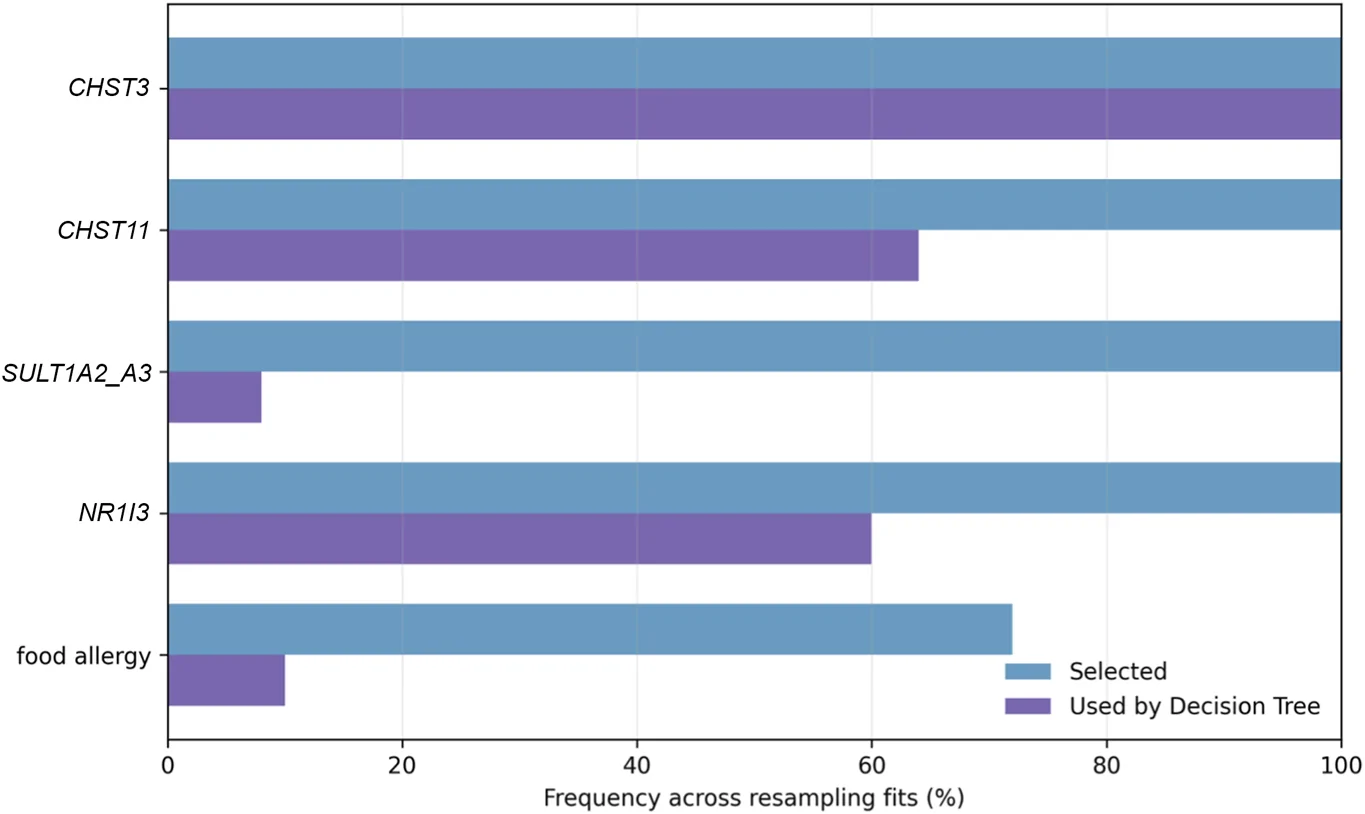
Feature-retention and decision-tree use frequencies for the investigated pharmacogenomic and clinical predictors across the 50 repeated cross-validation fits.

Overall, the complete decision tree showed the highest mean predictive performance, although its improvement over *CHST3* alone was modest. Feature-stability analysis further identiwed *CHST3* as the most reproducible predictor, whereas the other genomic and clinical variables contributed less consistently. Given the limited cohort and independent test-set sizes, these findings should be interpreted as proof-of-concept evidence supporting the potential usefulness of the proposed integrative methodology, rather than as validation of a definitive predictive model. Confirmation in larger, independent cohorts is required.

## 4 Discussion

This study provides exploratory evidence supporting the role of pharmacogenomic variability in modulating PPI response in pediatric EoE patients. In 58 patients (17 PPI-REs, 41 PPI-NORE), genotyping of ADME-related genes by DMET Plus, integrated with clinical parameters and variability in treatment response, as previously demonstrated [ 31, 32, 33, 38], we identified the G/G genotype in the *CHST3* gene (rs4148944) as potential novel candidate gene associated with PPI response, extending current pharmacogenomic frameworks beyond the well-established *CYP2C19* and *STAT6* pathways, not replicated in this study given the small sample size and potential variability in PPI type, dose, and treatment exposure. *STAT6*-related associations could not be evaluated because *STAT6* was not included among the genetic markers interrogated by the DMET Plus array. In addition, although *CYP3A5, ABCB1* and *ATP4A* have previously been discussed as potentially relevant to PPI pharmacology, they did not contribute to the genomic-clinical classification patterns identified in the present cohort. This discrepancy may reflect differences in cohort size, population characteristics, or genetic background, and further highlights the complexity of pharmacogenomic determinants in EoE.

In addition, the integrative ML analysis of the DMET dataset also revealed combinatorial patterns involving *CHST3, SULT1A2_A3*, and *NR1I3* variants, particularly in association with food allergy, which emerged as a relevant clinical feature contributing to patient stratification. Although some of these variants were not individually significant, their combined occurrence suggests that drug response in EoE may be influenced by interactions between metabolic, regulatory, and inflammatory pathways. Moreover, the decision tree algorithm identified “food allergy” as the variable that maximizes the separation between responders and non-responders while minimizing within-node class heterogeneity (Figure 3). These findings should be regarded as exploratory and require validation in larger, independent cohorts.

To our knowledge, no direct associations or specific studies for the SNPs identified in this study and PPI response in pediatric EoE have been reported; However, *CHST3* has emerged in a previous DMET-based pharmacogenomic study conducted in a different clinical setting, where variants in *CHST3* and *CHST13* were reported as candidate predictors of bosentan-induced liver toxicity in patients with pulmonary arterial hypertension (Yorifujia et al., 2018). Although that study involved a different drug, phenotype, disease context, and population, the recurrence of *CHST3* as a signal in relatively small array-based pharmacogenomic studies warrants a cautious interpretation. This observation may reflect either a broader involvement of sulfation-related pathways in interindividual variability in drug response or, alternatively, the increased likelihood of recurrent exploratory signals in studies characterized by limited sample size and multiple testing. Several lines of evidence nevertheless support a plausible biological role for these genes ientified in pathways relevant to the chronic inflammatory pathways underlying EoE. *CHST3* and *CHST11* encode carbohydrate sulfotransferase 3 and 11 respectively, involved in glycosaminoglycan (GAG) sulfation, a key process in extracellular matrix (ECM) organization (UniProt Consortium, 2026; OMIM, 2026; GeneCards, 2026). Carbohydrate sulfotransferases have been applied mainly in diagnostics of connective tissue disorders, cancer and inflammations (Begolli et al., 2023; Guan et al., 2021; Xiong et al., 2023; Zhang et al., 2020). Although *CHST3* and *CHST11* have a determinant role in cartilage and cornea formation, genetic polymorphisms in these genes could be involved in the modification of sulfation patterns which are involved in many biological functions. Alterations in sulfation patterns may affect cytokine binding, growth factor signaling, and mucosal barrier integrity, thereby influencing inflammatory responses and tissue remodeling. In this context, genetic variability in sulfation pathways may contribute to the establishment of a pro-inflammatory microenvironment, potentially affecting PPI responsiveness through changes in tissue remodeling and epithelial barrier function. Accordingly, *CHST3* rs4148944 should be regarded as a hypothesis-generating candidate associated with PPI response in pediatric EoE, with the novelty of this study residing in the specific clinical setting and the integrated pharmacogenomic-clinical decision-tree approach. Moreover, the involvement of the variants in *SULT1A2/A3* and *NR1I3* genes further supports the role of phase II metabolism and nuclear receptor–mediated regulation in drug response variability and may contribute to alterations in sulfation pathways influencing mucosal inflammation (Suiko et al., 2017; Hudson et al., 2017). The *SULT1A2* and *SULT1A3* genes encode phase II cytosolic sulfotransferase (SULT) enzymes that catalyze the conjugation of small molecules with sulfate groups, thereby enhancing their solubility and promoting excretion. These enzymes are involved in the biotransformation of endogenous and exogenous compounds, and their altered activity may impact both local mucosal homeostasis and systemic drug metabolism. Genetic polymorphisms within *SULT1A2/A3* may modify enzyme activity, leading to interindividual variability in drug metabolism, altered inflammatory responses, and disturbances in hormonal balance (Kurogi et al., 2021). The expression of these genes in response to chemical or hormonal stimuli is regulated by *NR1I3*, also known as the Constitutive Androstane Receptor (CAR**)**, a key nuclear receptor that modulates xenobiotic and hormone metabolism (Stern et al., 2022). Variations or altered activity of *NR1I3* may indirectly influence sulfation efficiency by modulating *SULT1A2/3* expression (Coughtrie, 2016). This regulatory cross-talk could play a significant role in inflammatory conditions, including EoE**.** Polymorphic variants in *CHST3*, *CHST11*, *SULT1A2/A3*, and *NR1I3* might alter enzyme activity or expression, leading to aberrant sulfation of glycosaminoglycans (GAGs) and disrupt cell signaling, inflammatory pathways, and tissue remodeling, thereby sustaining the chronic inflammation that underlies EoE pathogenesis. Furthermore, persistent ECM remodeling may diminish the bioavailability and pharmacological efficacy of PPIs. The mechanistic links between ECM sulfation pathways and PPI response remain speculative and require functional validation in dedicated experimental models before any clinical interpretation can be made. For *SULT1A2/A3* rs11859842 and *NR1I3* rs55802895, the historical DMET gene assignments were interpreted cautiously because current genomic annotation does not fully overlap with the original platform-based annotation.

The ML approach enabled the identification of interaction patterns that may not be readily detected using conventional univariate statistical methods. Nevertheless, the present analysis was designed primarily as a proof-of-concept to assess the potential usefulness of the proposed integrative methodology. It should not be interpreted as the development or validation of a definitive predictive model. Accordingly, the identified associations and decision rules should be regarded as exploratory and hypothesis-generating.

Several limitations should be acknowledged. The relatively small cohort and the high dimensionality of the genomic data increase the risk of overfitting and limit the generalizability of the findings. Although repeated stratified five-fold cross-validation was used to obtain more robust estimates of model performance, the resulting distributions still showed substantial variability. Moreover, the small independent test set limits the precision of the external performance estimates and does not permit a definitive assessment of clinical utility.

The findings from several performance evaluation metrics suggest that *CHST3* accounts for most of the predictive information captured by the complete model, whereas the additional genomic and clinical variables provide only limited incremental discrimination.

ROC curve analysis yielded an area under the curve (ROC-AUC) of 0.781 on the independent hold-out test set, indicating moderate discrimination between PPI-RE and PPI-NORE patients. Despite these encouraging results, the performance estimates should be interpreted with considerable caution.

The hold-out test set included only 12 of the 58 enrolled subjects (20.7%), whereas the remaining 46 subjects were assigned to the development set. Although the two subsets were strictly non-overlapping and the test set was not used during preprocessing, feature selection, model fitting, or cross-validation, its limited size substantially reduces the precision and stability of the performance estimates. In such a small test set, the classification of even a single additional patient could result in a marked change in the reported metrics. Therefore, the observed ROC-AUC should not be considered evidence of definitive model validation or clinical utility.

However, given the very limited number of test samples, these estimates are inherently imprecise and cannot establish the superiority or clinical applicability of the complete model. Overall, the results support the potential value of the proposed methodology for integrating pharmacogenomic and clinical variables and generating candidate predictive patterns. Further studies involving larger, independent, and preferably multicentre cohorts are required to evaluate the reproducibility of the identified features, refine the model, and determine whether the modest improvement beyond *CHST3* is clinically meaningful. A further limitation concerns the nominal deviation from HWE observed for rs4148944. Although this deviation did not remain significant after FDR correction and the variant therefore met the predefined retention criterion, the finding warrants caution because deviations from HWE may reflect sampling variability or population structure. Given the small cohort size and the central role of this variant in the present analysis, independent replication would strengthen the reliability of this association.

Although the DMET platform provides targeted coverage of pharmacogenomically relevant ADME genes, it does not capture the full spectrum of genomic variability, including variants identified in genome-wide association studies (GWAS), or genes involved in immune regulation and epithelial function (Sissung et al., 2010). Furthermore, gene–environment interactions and epigenetic factors were not assessed and may contribute to variability in treatment response. Variants in non-ADME genes involved in immune regulation, epithelial integrity, or eosinophilic trafficking may also act as modifiers of histologic outcome.

This work provides one of the few examples demonstrating that combining pharmacogenomic and clinical endotype data can help to identify EoE patient subgroups with distinct PPI responsiveness, paving the way toward more tailored strategies. Moreover, our findings support the relevance of precision medicine approaches in EoE, whereby genetic and immunologic profiling could assist clinicians in selecting the most appropriate therapy from the outset. While PPIs remain a convenient and cost-effective first-line option, identifying patients who are unlikely to respond could prevent delays in achieving remission and reduce the number of unnecessary procedures.

Lastly, it is worth noting that recent efforts in GWAS studies such as those by Shook et al. (2024), have expanded the understanding of common polymorphisms associated with EoE risk, identifying regulatory variants that affect transcriptional activity. These studies provide a broader framework in which our findings on *CHST3* can be contextualized, pointing toward new candidate pathways involved in PPI resistance.

In addition, given the retrospective assessment of the clinical data, a minority of patients received different PPIs or treatment durations despite the existence of a standard hospital protocol. However, all patients received at least the guideline-recommended minimum dose and duration for PPIs, and all PPIs belonged to the same drug class, which regulatory agencies and clinical practice guidelines generally consider interchangeable. No significant differences in PPI type distribution or treatment duration between PPI-RE and PPI-NORE patients were registered.

## 5 Conclusion

In conclusion, this study provides proof-of-concept evidence that pharmacogenomic variability in ADME-related and extracellular matrix–associated pathways may contribute to interindividual differences in PPI response in pediatric EoE. The identification of *CHST3* and related pathways suggests a potential link between sulfation processes, tissue microenvironment, and pharmacological response. We hypothesized that specific genetic patterns could contribute to the classification of EoE patients based on their response to PPIs, through the identification of SNPs and genotypes potentially associated with both drug response and food allergy phenotypes. The ML approach adopted in this study has allowed the integration of genetic and clinical data and may support the identification of biologically relevant patient subgroups, contributing to more personalized therapeutic strategies. However, these findings require validation in larger, independent cohorts before clinical application can be considered.

Overall, integrating pharmacogenomic data (e.g., *CHST3* polymorphisms) with clinical allergy profiling—food allergy—may represent a promising opportunity toward a more precise, personalized approach to pediatric EoE. Future studies integrating additional omics layers, including transcriptomic and proteomic data, will be essential to further elucidate the mechanisms underlying PPI responsiveness and will allow external validation required for a universal diagnostic platform.

## Data Availability

The datasets generated for this study contain individual clinical data from a small series of patients with a rare disease and cannot be made publicly available, because the risk of re-identification persists despite de-identification and the informed consent obtained does not cover public deposit. Anonymized minimal datasets underlying the findings are available on reasonable request to the corresponding author, subject to Ethics Committee approval, data use agreement and informed consent to public deposit.
