# Supplementary material for: Pharmacogenomic predictors of proton pump inhibitor response in pediatric eosinophilic esophagitis
Source: Front Pharmacol. 2026 Oct 30;17:1913372. doi: 10.3389/fphar.2026.1913372 (PMC13759821; doi:10.3389/fphar.2026.1913372)
Supplement: Supplementary file 1 [file DataSheet1.pdf]

Supplementary 1.

Supplementary Table 1. CYP2C19 variants in the analysis of PPI response.

Rank	Gene	Probe set ID / dbSNP ID	Responders, genotype counts	Non-responders, genotype counts	Uncorrected p-value	FDR-adjusted p-value (BH)
1	CYP2C19	AM_10075<br>rs3758581	A/G 6; G/G 10	A/G 7; G/G 34	0.1578	1.0000
2	CYP2C19	AM_10070<br>rs72547532	A/A 1; A/G 4; G/G 12	A/G 13; G/G 28	0.2994	1.0000
3	CYP2C19	AM_10064<br>rs17884712|rs28399507	G/G 16	A/G 4; G/G 33	0.3025	1.0000
4	CYP2C19	AM_10056<br>rs17882687	A/A 16	A/A 35; A/C 5	0.3068	1.0000
5	CYP2C19	AM_10053<br>rs12248560	C/C 10; C/T 7	C/C 29; C/T 11; T/T 1	0.3541	1.0000
6	CYP2C19	AM_10054<br>rs28399504	A/A 17	A/A 40	1.0000	1.0000
7	CYP2C19	AM_10055<br>rs55752064	T/T 17	T/T 39	1.0000	1.0000
8	CYP2C19	AM_10059<br>rs17878459	G/G 17	C/G 1; G/G 34	1.0000	1.0000
9	CYP2C19	AM_10060<br>rs41291556	T/T 17	T/T 41	1.0000	1.0000
10	CYP2C19	AM_10062<br>rs72558184	G/G 17	G/G 41	1.0000	1.0000
11	CYP2C19	AM_10068<br>rs4986893	G/G 17	G/G 39	1.0000	1.0000
12	CYP2C19	AM_10069<br>rs6413438	C/C 16	C/C 39	1.0000	1.0000
13	CYP2C19	AM_10071<br>rs72558185	-/- 17	-/- 36	1.0000	1.0000
14	CYP2C19	AM_10072<br>rs72558186	T/T 17	T/T 37	1.0000	1.0000
15	CYP2C19	AM_10077<br>rs17879685	C/C 17	C/C 41	1.0000	1.0000
16	CYP2C19	AM_10079<br>rs56337013	C/C 17	C/C 37	1.0000	1.0000
17	CYP2C19	AM_10080<br>rs5787121	G/G 17	G/G 38	1.0000	1.0000
18	CYP2C19	AM_10082<br>rs55640102	A/A 15	A/A 33	1.0000	1.0000

*The analyzed cohort comprised 17 responders and 41 non-responders. NoCall, PossibleRareAllele, blank, and missing calls were excluded on a variant-specific basis; therefore, Valid N may be lower than 17/41.

Supplementary Table 2. CYP2C19 diplotype-derived metabolizer phenotypes according to PPI response

CYP2C19 diplotype(s) identified in the cohort	CPIC-predicted metabolizer phenotype	PPI responders (n=17), n (%)	PPI non-responders (n=41), n (%)	Two-sided Fisher's exact p-value[#]
*17/*17	Ultrarapid metabolizer (UM)	0 (0.0%)	1 (2.4%)	1.0000
*1/*17; *15/*17	Rapid metabolizer (RM)	5 (29.4%)	5 (12.2%)	0.1388
*1/*1	Normal metabolizer (NM)	4 (23.5%)	17 (41.5%)	0.2411
*1/*2A; *1/*2B; *2A/*17; *2A/*15; *2B/*15	Intermediate metabolizer (IM)	5 (29.4%)	9 (22.0%)	0.7369
*9/*15	Likely intermediate metabolizer (likely IM)	0 (0.0%)	2 (4.9%)	1.0000
*2A/*2A	Poor metabolizer (PM)	1 (5.9%)	0 (0.0%)	0.2931
*2A/*9	Likely poor metabolizer (likely PM)	0 (0.0%)	2 (4.9%)	1.0000
Ambiguous DMET calls spanning more than one CPIC phenotype	Indeterminate	2 (11.8%)	5 (12.2%)	1.0000
Total		17 (100%)	41 (100%)	—

[#] Two-sided Fisher's exact test comparing each phenotype category with all remaining phenotype categories between PPI responders and non-responders. Phenotypes were assigned according to current CPIC CYP2C19 diplotype-to-phenotype definitions. Rapid metabolizers and ultrarapid metabolizers were retained as separate categories, as recommended by current CPIC nomenclature. DMET calls compatible with more than one CPIC phenotype were conservatively classified as indeterminate rather than forcing a phenotype assignment. "Likely" intermediate and poor metabolizer categories reflect diplotypes containing decreased-function alleles for which CPIC assigns a likely phenotype.

Supplementary Table 3. Exploratory comparison of CYP2C19 rapid/ultrarapid metabolizer phenotypes according to PPI response

Exploratory comparison	PPI responders (n=17)	PPI non-responders (n=41)	Two-sided Fisher's exact p-value
RM + UM	5 (29.4%)	6 (14.6%)	0.2704
Other phenotypes	12 (70.6%)	35 (85.4%)	

Rapid (RM) and ultrarapid metabolizers (UM) were combined in an exploratory analysis and compared with all other evaluable CYP2C19 metabolizer phenotypes. Values are reported as n (%).

Supplementary Table 4. Top-ranked SNPs in the analysis of PPI response.

Rank	Gene	Probe set ID / dbSNP ID	Responders, genotype counts	Non-responders, genotype counts	Uncorrected p-value	FDR-adjusted p-value (BH)
1	CHST3	AM_10004 rs4148944	A/A 0; A/G 0; G/G 17	A/A 4; A/G 9; G/G 28	0.0064	0.0643
2	CHST3	AM_10003 rs4148943	C/C 5; C/T 7; T/T 4	C/C 10; C/T 25; T/T 4	0.2604	1.0000
3	CHST3	AM_10005 rs4148945	C/C 6; C/T 7; T/T 4	C/C 14; C/T 24; T/T 4	0.3140	1.0000
4	CHST3	AM_10011 rs4148950	A/A 4; A/G 7; G/G 6	A/A 5; A/G 24; G/G 11	0.3543	1.0000
5	CHST3	AM_10014 rs731027	C/C 3; C/T 7; T/T 6	C/C 6; C/T 23; T/T 12	0.6628	1.0000
6	CHST3	AM_10013 rs1871450	A/A 4; A/G 7; G/G 6	A/A 8; A/G 23; G/G 12	0.6963	1.0000
7	CHST3	AM_10016 rs730720	C/C 5; C/T 7; T/T 3	C/C 12; C/T 24; T/T 5	0.7072	1.0000
8	CHST3	AM_10010 rs4148949	C/C 5; C/T 8; T/T 4	C/C 10; C/T 22; T/T 9	0.9316	1.0000
9	CHST3	AM_10012 rs4148951	G/G 16	A/G 2; G/G 37	1.0000	1.0000
10	CHST3	AM_10001 rs4148942	C/C 16	C/C 41; C/G 1	1.0000	1.0000
11	CHST3	AM_10006 rs4148946	C/C 3; C/T 8; T/T 4	C/C 10; C/T 20; T/T 12	1.0000	1.0000
12	CHST3	AM_10008 rs7072219	A/A 17	A/A 39; A/C 2	1.0000	1.0000
13	CHST3	AM_10017 rs4148953	G/G 17	A/G 1; G/G 41	1.0000	1.0000

Supplementary Table 5. Individual association results for CHST11 rs7847, SULT1A2/A3 rs11859842, and NR1I3 rs55802895.

Gene/SNP	Responder: genotype, n (%)	Non-responder: genotype, n (%)	OR (95% CI)	p-value	FDR-corrected p-value
CHST11 rs7847	T/T: 13 (76.5%); C/T: 4 (23.5%); C/C: 0	T/T: 21 (51.2%); C/T: 16 (39.0%); C/C: 4 (9.8%)	0.32 (0.09–1.16)	0.0885	0.2081
SULT1A2/A3 rs11859842	G/G: 4 (23.5%); A/G: 8 (47.1%); A/A: 5 (29.4%)	G/G: 2 (4.9%); A/G: 22 (53.7%); A/A: 17 (41.5%)	1.70 (0.50–5.73)	0.5536	0.5536
NR1I3 rs55802895	A/A: 3 (17.6%); A/G: 2 (11.8%); G/G: 12 (70.6%)	A/A: 0; A/G: 5 (12.2%); G/G: 36 (87.8%)	3.00 (0.74–12.18)	0.1388	0.2081

Genotype distributions are reported as n (%). Odds ratios (ORs) with 95% confidence intervals (CIs), uncorrected p-values, and FDR-adjusted p-values are reported. None of the associations remained statistically significant after multiple-testing correction.

Supplementary Table 6. Updated genomic annotation of variants retained in the final decision-tree/rule-based analysis

Probe set ID	dbSNP ID	Original DMET gene assignment	Genome build	Genomic coordinate	Reference transcript	Consequence / genomic context	Current annotation / comment
AM_10004	rs4148944	CHST3	GRCh38	chr10:72,009,785	NM_004273.5	_*3′ UTR variant, c.1314G>A_	DMET and current annotation concordant: CHST3
AM_10579	rs7847	CHST11	GRCh38	chr12:104,761,827	NM_018413.6	_*3′ UTR variant, c.4024C>T_	DMET and current annotation concordant: CHST11
AM_11005*	rs11859842	SULT1A2_A3	GRCh38	chr16:29,557,397	N/A – intergenic	Intergenic variant	Historical DMET assignment; current annotation does not support an unequivocal intragenic SULT1A2/A3 assignment. Other DMET-based datasets have assigned the same rsID to CA5P.
AM_11834	rs55802895	NR1I3	GRCh38	chr1:161,242,953	N/A – upstream/5′ flanking	5′ flanking/regulatory variant	Located in the upstream regulatory region of NR1I3

Original DMET Console gene assignments are shown together with updated GRCh38 genomic coordinates, reference transcripts, and molecular consequences. Discrepancies between historical DMET annotation and current genomic context are explicitly reported.

Original gene assignments correspond to the DMET Console annotation library used for variant calling. For variants retained in the final predictive model, these platform-based annotations were cross-checked against current genomic reference resources. Where historical DMET gene assignments were not fully concordant with current genomic annotation, both the original probe assignment and the current genomic context are reported.

*

```
475     AM_11021        78.6    11.9    0       2       0.106   0.894   0.106   NaN     NaN     NaN     NaN     SULT1A2_A3      16
        SULT1A2_A3_>(rs1364182) rs1364182       29563594        -1      G // T  A // B  N/A     N/A     G // T  N/A     snp     Y
                N/A             N       CEU|59|87.3|12.7|0.0|CHB|90|46.1|53.9|0.0|CHD|87|49.4|50.6|0.0|JPT|91|28.6|71.4|
0.0|LWK|89|64.0|36.0|0.0|MEX|60|57.5|42.5|0.0|YRI|119|56.3|43.7|0.0
476     AM_11022        83.3    52.4    0       2       0.371   0.629   0.371   NaN     NaN     NaN     NaN     SULT1A2_A3      16
        SULT1A2_A3_>(rs11859842)        rs11859842      29568718        -1      A // G  A // B  N/A     N/A     A // G  N/A     snp
        Y                       N/A             N       CEU|59|50.8|49.2|0.0|CHB|90|97.2|2.8|0.0|CHD|87|97.7|2.3|0.0|JPT|91|98.4|
1.6|0.0|LWK|89|90.4|9.6|0.0|MEX|60|75.8|24.2|0.0|YRI|119|87.0|13.0|0.0
477     AM_11023        85.7    38.1    0       2       0.444   0.444   0.556   NaN     NaN     NaN     NaN     SULT1A2_A3      16
        SULT1A2_A3_>(rs11150564)        rs11150564      29574237        -1      C // T  A // B  N/A     N/A     C // T  N/A     snp
        Y                       N/A             N       CEU|59|55.9|44.1|0.0|CHB|90|65.0|35.0|0.0|CHD|87|66.7|33.3|0.0|JPT|91|76.9|
```

Supplementary Table 7. Univariable and multivariable Firth-penalized logistic regression analyses of PPI response.

Predictor	Comparison	Univariable OR	95% profile penalized-likelihood CI	p-value	Multivariable OR	95% profile penalized-likelihood CI	p-value
CHST3 rs4148944	G/G vs non-G/G	16.58	1.97–2170.19	0.0050	13.65	1.36–1874.67	0.0221
CHST11 rs7847	T/T vs non-T/T	2.86	0.88–10.74	0.0803	2.28	0.56–10.96	0.2540
SULT1A2/A3 rs11859842	A/A vs non-A/A	0.62	0.18–1.94	0.4129	1.01	0.22–4.34	0.9875
NR1I3 rs55802895	A/A vs non-A/A	20.03	1.79–2757.03	0.0125	17.30	1.40–2586.46	0.0236
IgE-mediated food allergy	Present vs absent	0.43	0.08–1.70	0.2405	0.23	0.02–1.13	0.0720

ORs and 95% CIs were estimated using Firth's bias-reduced penalized logistic regression. Confidence intervals were obtained using profile penalized likelihood. PPI response was coded as 1 and non-response as 0; food allergy was coded as present versus absent.
