# Supplementary material for: Pharmacogenomic predictors of proton pump inhibitor response in pediatric eosinophilic esophagitis
Source: Front Pharmacol. 2026 Oct 30;17:1913372. doi: 10.3389/fphar.2026.1913372 (PMC13759821; doi:10.3389/fphar.2026.1913372)
Supplement: Supplementary file 2 [file DataSheet2.pdf]

Result no.	Probe Set ID	Category 1	ClassA (n), category 1	ClassB (n), category 1	Category 2	ClassA (n), category 2	ClassB (n), category 2	Fisher exact test p-value	FDR correction
1	AM_10004	NO_G/G	0	13	G/G	17	28	7.000000E-03	6.433000E-03

Table 1. The full DMETAnalyzer results after applying the FDR correction

Table 2. The full DMETAnalyzer results without any statistical correction.

Result no.	Probe Set ID	Category 1	ClassA (n), category 1	ClassB (n), category 1	Category 2	ClassA (n), category 2	ClassB (n), category 2	Fisher exact test p-value	Multiple-testing correction
1	AM_10004	NO_G/G	0	13	G/G	17	28	7.00E-03	Without correction
2	AM_11834	C/C	14	16	NO_C/C	3	25	3.64E-03	Without correction
3	AM_14166	A/A	8	5	A/G	5	26	8.64E-03	Without correction
4	AM_11264	NO_T/T	15	21	T/T	2	20	8.76E-03	Without correction
5	AM_11834	C/C	14	16	C/T	3	21	8.86E-03	Without correction
6	AM_10659	G/G	14	18	NO_G/G	3	23	9.37E-03	Without correction
7	AM_13871	NO_A/G	10	37	A/G	7	4	9.88E-03	Without correction
8	AM_14166	A/A	8	5	NO_A/A	9	36	1.22E-02	Without correction
9	AM_13871	G/G	10	35	A/G	7	4	1.24E-02	Without correction
10	AM_11318	NO_G/G	10	10	G/G	7	31	1.69E-02	Without correction
11	AM_10659	G/G	14	18	A/G	3	22	1.81E-02	Without correction
12	AM_10659	NO_A/G	14	19	A/G	3	22	1.87E-02	Without correction
13	AM_11500	A/A	4	1	A/G	6	23	1.90E-02	Without correction
14	AM_10401	C/C	5	27	NO_C/C	12	14	1.92E-02	Without correction
15	AM_12535	A/C	4	20	A/A	7	5	1.99E-02	Without correction
16	AM_13464	A/T	14	20	NO_A/T	3	21	2.16E-02	Without correction
17	AM_11834	NO_C/T	14	20	C/T	3	21	2.16E-02	Without correction
18	AM_11500	A/A	4	1	NO_A/A	13	40	2.26E-02	Without correction
19	AM_11264	G/T	11	17	T/T	2	20	2.27E-02	Without correction
20	AM_14401	NO_G/G	16	26	G/G	1	15	2.28E-02	Without correction
21	AM_14166	NO_A/G	12	15	A/G	5	26	2.31E-02	Without correction
22	AM_13439	NO_G/G	17	30	G/G	0	11	2.38E-02	Without correction
23	AM_10004	G/G	17	28	A/G	0	9	4.41E-02	Without correction
24	AM_10390	NO_C/C	9	34	C/C	8	7	2.47E-02	Without correction
25	AM_10401	C/C	5	27	T/T	5	4	2.55E-02	Without correction
26	AM_15210	G/T	9	13	T/T	1	14	2.79E-02	Without correction
27	AM_12535	NO_A/A	10	36	A/A	7	5	2.83E-02	Without correction
28	AM_14705	G/G	6	28	A/G	9	10	2.91E-02	Without correction
29	AM_11264	G/G	4	4	T/T	2	20	2.94E-02	Without correction
30	AM_11318	A/G	9	10	G/G	7	31	3.07E-02	Without correction
31	AM_13153	A/A	14	22	A/G	2	17	3.27E-02	Without correction
32	AM_13153	NO_A/G	15	24	A/G	2	17	3.42E-02	Without correction
33	AM_10390	C/C	8	7	C/T	5	22	3.52E-02	Without correction
34	AM_14401	G/T	12	20	G/G	1	15	3.63E-02	Without correction
35	AM_10603	A/G	7	25	G/G	5	3	3.86E-02	Without correction
36	AM_14705	G/G	6	28	NO_G/G	11	13	3.86E-02	Without correction
37	AM_13871	G/G	10	35	NO_G/G	7	6	3.99E-02	Without correction
38	AM_10714	NO_G/G	12	38	G/G	5	3	4.01E-02	Without correction
39	AM_10603	NO_G/G	12	38	G/G	5	3	4.01E-02	Without correction
40	AM_12126	A/C	6	26	A/A	3	1	4.06E-02	Without correction
41	AM_13439	G/T	11	20	G/G	0	11	4.07E-02	Without correction
42	AM_12092	C/T	10	12	NO_C/T	7	29	4.30E-02	Without correction

Result no.	Probe Set ID	Category 1	ClassA (n), category 1	ClassB (n), category 1	Category 2	ClassA (n), category 2	ClassB (n), category 2	Fisher exact test p-value	Multiple-testing correction
43	AM_15086	T/T	12	16	NO_T/T	5	25	4.32E-02	Without correction
44	AM_13775	NO_A/G	12	16	A/G	5	25	4.32E-02	Without correction
45	AM_10440	A/A	6	27	NO_A/A	11	14	4.37E-02	Without correction
46	AM_15210	NO_T/T	16	27	T/T	1	14	4.48E-02	Without correction
47	AM_14759	NO_A/G	16	27	A/G	1	14	4.48E-02	Without correction
48	AM_14759	G/G	16	27	NO_G/G	1	14	4.48E-02	Without correction
49	AM_14759	G/G	16	27	A/G	1	14	4.48E-02	Without correction
50	AM_10634	NO_G/T	16	27	G/T	1	14	4.48E-02	Without correction
51	AM_10634	G/G	16	27	NO_G/G	1	14	4.48E-02	Without correction
52	AM_10634	G/G	16	27	G/T	1	14	4.48E-02	Without correction
53	AM_12114	NO_C/G	16	28	C/G	1	13	4.58E-02	Without correction
54	AM_12114	G/G	16	28	NO_G/G	1	13	4.58E-02	Without correction
55	AM_12114	G/G	16	28	C/G	1	13	4.58E-02	Without correction
56	AM_12093	G/G	16	28	NO_G/G	1	13	4.58E-02	Without correction
57	AM_12461	C/G	6	21	G/G	6	4	4.86E-02	Without correction
58	AM_12461	NO_G/G	11	37	G/G	6	4	4.99E-02	Without correction
59	AM_13635	NO_C/C	13	39	C/C	4	2	5.48E-02	Without correction
60	AM_11416	G/G	4	2	NO_G/G	13	39	5.48E-02	Without correction
61	AM_11022	NO_G/G	13	39	G/G	4	2	5.48E-02	Without correction
62	AM_14705	NO_A/G	8	31	A/G	9	10	6.30E-02	Without correction
63	AM_11318	A/G	9	10	NO_A/G	8	31	6.30E-02	Without correction
64	AM_11023	C/C	6	5	NO_C/C	11	36	6.45E-02	Without correction
65	AM_10361	NO_T/T	8	9	T/T	9	32	6.70E-02	Without correction
66	AM_10361	A/T	8	9	NO_A/T	9	32	6.70E-02	Without correction
67	AM_13979	NO_C/C	14	40	C/C	3	1	7.13E-02	Without correction
68	AM_13337	NO_A/A	14	40	A/A	3	1	7.13E-02	Without correction
69	AM_12126	NO_A/A	14	40	A/A	3	1	7.13E-02	Without correction
70	AM_13153	A/A	14	22	NO_A/A	3	19	7.26E-02	Without correction
71	AM_13437	A/C	10	13	NO_A/C	7	28	7.81E-02	Without correction
72	AM_10592	NO_A/G	10	13	A/G	7	28	7.81E-02	Without correction
73	AM_13256	NO_C/T	13	20	C/T	4	21	8.04E-02	Without correction
74	AM_12126	A/C	6	26	NO_A/C	11	15	8.09E-02	Without correction
75	AM_14347	T/T	12	17	NO_T/T	5	24	8.19E-02	Without correction
76	AM_13256	C/C	12	17	NO_C/C	5	24	8.19E-02	Without correction
77	AM_12140	T/T	2	0	NO_T/T	15	41	8.23E-02	Without correction
78	AM_11835	A/A	2	0	NO_A/A	15	41	8.23E-02	Without correction
79	AM_11554	T/T	2	0	NO_T/T	15	41	8.23E-02	Without correction
80	AM_10048	T/T	2	0	NO_T/T	15	41	8.23E-02	Without correction
81	AM_13365	NO_A/A	16	29	A/A	1	12	8.25E-02	Without correction
82	AM_12093	NO_A/G	16	29	A/G	1	12	8.25E-02	Without correction
83	AM_10090	NO_A/C	16	30	A/C	1	11	8.85E-02	Without correction
84	AM_10090	A/A	16	30	NO_A/A	1	11	8.85E-02	Without correction
85	AM_12535	A/C	4	20	NO_A/C	13	21	8.85E-02	Without correction
86	AM_10579	T/T	13	21	NO_T/T	4	20	8.85E-02	Without correction
87	AM_11325	NO_G/G	7	7	G/G	10	34	8.89E-02	Without correction
88	AM_11325	A/G	7	7	NO_A/G	10	34	8.89E-02	Without correction
89	AM_11024	G/G	17	33	NO_G/G	0	8	9.00E-02	Without correction
90	AM_13365	NO_A/G	6	25	A/G	11	16	9.01E-02	Without correction
91	AM_11296	NO_T/T	11	16	T/T	6	25	9.01E-02	Without correction
92	AM_11024	NO_A/G	17	34	A/G	0	7	9.34E-02	Without correction
93	AM_10730	NO_C/C	17	34	C/C	0	7	9.34E-02	Without correction
94	AM_10401	NO_T/T	12	37	T/T	5	4	1.06E-01	Without correction

Result no.	Probe Set ID	Category 1	ClassA (n), category 1	ClassB (n), category 1	Category 2	ClassA (n), category 2	ClassB (n), category 2	Fisher exact test p-value	Multiple-testing correction
95	AM_12285	NO_C/C	7	8	C/C	10	33	1.07E-01	Without correction
96	AM_12285	C/T	7	8	NO_C/T	10	33	1.07E-01	Without correction
97	AM_14146	NO_A/G	15	26	A/G	2	15	1.11E-01	Without correction
98	AM_13253	NO_G/G	15	26	G/G	2	15	1.11E-01	Without correction
99	AM_14814	NO_A/G	15	27	A/G	2	14	1.12E-01	Without correction
100	AM_13647	NO_G/G	9	31	G/G	8	10	1.22E-01	Without correction
101	AM_12523	NO_G/G	9	31	G/G	8	10	1.22E-01	Without correction
102	AM_11253	NO_C/T	14	24	C/T	3	17	1.29E-01	Without correction
103	AM_10611	A/A	14	24	NO_A/A	3	17	1.29E-01	Without correction
104	AM_14363	A/G	9	12	NO_A/G	8	29	1.33E-01	Without correction
105	AM_12647	NO_G/G	9	12	G/G	8	29	1.33E-01	Without correction
106	AM_12647	A/G	9	12	NO_A/G	8	29	1.33E-01	Without correction
107	AM_10243	NO_T/T	9	12	T/T	8	29	1.33E-01	Without correction
108	AM_11669	C/T	3	16	NO_C/T	14	25	1.37E-01	Without correction
109	AM_10675	NO_T/T	12	36	T/T	5	5	1.39E-01	Without correction
110	AM_11456	NO_A/G	7	27	A/G	10	14	1.42E-01	Without correction
111	AM_15086	NO_C/T	13	22	C/T	4	19	1.44E-01	Without correction
112	AM_13207	NO_T/T	14	39	T/T	3	2	1.44E-01	Without correction
113	AM_15000	C/C	12	19	NO_C/C	5	22	1.48E-01	Without correction
114	AM_11520	NO_C/T	12	19	C/T	5	22	1.48E-01	Without correction
115	AM_11520	C/C	12	19	NO_C/C	5	22	1.48E-01	Without correction
116	AM_10597	NO_A/C	12	19	A/C	5	22	1.48E-01	Without correction
117	AM_10390	NO_C/T	12	19	C/T	5	22	1.48E-01	Without correction
118	AM_14444	NO_G/G	5	22	G/G	12	19	1.48E-01	Without correction
119	AM_13600	A/G	5	22	NO_A/G	12	19	1.48E-01	Without correction
120	AM_12206	NO_C/G	5	22	C/G	12	19	1.48E-01	Without correction
121	AM_13441	NO_A/T	16	31	A/T	1	10	1.48E-01	Without correction
122	AM_13441	A/A	16	31	NO_A/A	1	10	1.48E-01	Without correction
124	AM_13019	NO_A/A	16	31	A/A	1	10	1.48E-01	Without correction
125	AM_10691	T/T	16	31	NO_T/T	1	10	1.48E-01	Without correction
126	AM_15210	G/T	9	13	NO_G/T	8	28	1.49E-01	Without correction
127	AM_13148	C/C	6	24	NO_C/C	11	17	1.51E-01	Without correction
128	AM_11456	G/G	6	24	NO_G/G	11	17	1.51E-01	Without correction
129	AM_11264	NO_G/T	6	24	G/T	11	17	1.51E-01	Without correction
130	AM_14226	C/T	11	17	NO_C/T	6	24	1.51E-01	Without correction
131	AM_15285	NO_T/T	11	35	T/T	6	6	1.51E-01	Without correction
132	AM_10673	NO_G/T	11	35	G/T	6	6	1.51E-01	Without correction
133	AM_12181	G/G	10	15	NO_G/G	7	26	1.51E-01	Without correction
134	AM_10597	NO_A/A	7	26	A/A	10	15	1.51E-01	Without correction
135	AM_10270	NO_T/T	12	20	T/T	5	21	1.56E-01	Without correction
136	AM_15200	NO_C/T	5	21	C/T	12	20	1.56E-01	Without correction
137	AM_14401	NO_G/T	5	21	G/T	12	20	1.56E-01	Without correction
138	AM_11255	NO_C/G	11	34	C/G	6	7	1.71E-01	Without correction
139	AM_11255	G/G	11	34	NO_G/G	6	7	1.71E-01	Without correction
140	AM_10633	NO_A/A	11	34	A/A	6	7	1.71E-01	Without correction
141	AM_13727	T/T	4	3	NO_T/T	13	38	1.78E-01	Without correction
142	AM_15307	NO_G/G	13	38	G/G	4	3	1.78E-01	Without correction
143	AM_12204	NO_G/G	15	28	G/G	2	13	1.88E-01	Without correction
144	AM_14306	NO_A/G	15	29	A/G	2	12	1.95E-01	Without correction
145	AM_14306	A/A	15	29	NO_A/A	2	12	1.95E-01	Without correction
146	AM_13464	NO_T/T	15	29	T/T	2	12	1.95E-01	Without correction
147	AM_11334	NO_A/G	15	29	A/G	2	12	1.95E-01	Without correction

Result no.	Probe Set ID	Category 1	ClassA (n), category 1	ClassB (n), category 1	Category 2	ClassA (n), category 2	ClassB (n), category 2	Fisher exact test p-value	Multiple-testing correction
148	AM_11334	G/G	15	29	NO_G/G	2	12	1.95E-01	Without correction
149	AM_10440	NO_A/G	10	32	A/G	7	9	1.97E-01	Without correction
150	AM_13632	NO_A/A	7	9	A/A	10	32	1.97E-01	Without correction
151	AM_13632	A/G	7	9	NO_A/G	10	32	1.97E-01	Without correction
152	AM_11554	NO_C/C	7	9	C/C	10	32	1.97E-01	Without correction
153	AM_10143	C/T	7	9	NO_C/T	10	32	1.97E-01	Without correction
154	AM_14146	NO_G/G	15	40	G/G	2	1	2.03E-01	Without correction
155	AM_10183	NO_T/T	15	40	T/T	2	1	2.03E-01	Without correction
156	AM_11264	G/G	4	4	NO_G/G	13	37	2.16E-01	Without correction
157	AM_11253	C/C	4	4	NO_C/C	13	37	2.16E-01	Without correction
158	AM_10592	G/G	4	4	NO_G/G	13	37	2.16E-01	Without correction
159	AM_10031	NO_T/T	13	37	T/T	4	4	2.16E-01	Without correction
160	AM_14929	NO_A/G	14	26	A/G	3	15	2.17E-01	Without correction
161	AM_14929	G/G	14	26	NO_G/G	3	15	2.17E-01	Without correction
162	AM_14913	NO_C/C	14	26	C/C	3	15	2.17E-01	Without correction
163	AM_10611	NO_A/G	14	26	A/G	3	15	2.17E-01	Without correction
164	AM_10498	NO_A/C	14	26	A/C	3	15	2.17E-01	Without correction
165	AM_12523	A/G	3	15	NO_A/G	14	26	2.17E-01	Without correction
166	AM_14227	C/T	9	30	NO_C/T	8	11	2.18E-01	Without correction
167	AM_12140	NO_C/C	8	11	C/C	9	30	2.18E-01	Without correction
168	AM_10243	A/T	8	11	NO_A/T	9	30	2.18E-01	Without correction
169	AM_10401	NO_C/T	10	31	C/T	7	10	2.21E-01	Without correction
170	AM_10143	NO_C/C	7	10	C/C	10	31	2.21E-01	Without correction
171	AM_11804	NO_G/G	9	29	G/G	8	12	2.33E-01	Without correction
172	AM_14661	T/T	8	12	NO_T/T	9	29	2.33E-01	Without correction
174	AM_11875	A/G	8	12	NO_A/G	9	29	2.33E-01	Without correction
175	AM_11482	NO_C/C	8	12	C/C	9	29	2.33E-01	Without correction
176	AM_11047	C/T	8	12	NO_C/T	9	29	2.33E-01	Without correction
177	AM_12160	NO_C/T	13	23	C/T	4	18	2.34E-01	Without correction
178	AM_10030	C/C	13	23	NO_C/C	4	18	2.34E-01	Without correction
179	AM_11669	NO_T/T	4	18	T/T	13	23	2.34E-01	Without correction
180	AM_11066	NO_A/G	13	24	A/G	4	17	2.41E-01	Without correction
181	AM_15380	T	4	17	NO_T	13	24	2.41E-01	Without correction
182	AM_13253	A/A	9	14	NO_A/A	8	27	2.42E-01	Without correction
183	AM_13019	A/G	9	14	NO_A/G	8	27	2.42E-01	Without correction
184	AM_11968	C/T	9	14	NO_C/T	8	27	2.42E-01	Without correction
185	AM_11296	C/T	9	14	NO_C/T	8	27	2.42E-01	Without correction
186	AM_14871	C/C	12	21	NO_C/C	5	20	2.47E-01	Without correction
187	AM_14347	NO_C/T	12	21	C/T	5	20	2.47E-01	Without correction
188	AM_13400	A/G	12	21	NO_A/G	5	20	2.47E-01	Without correction
189	AM_12181	NO_A/G	12	21	A/G	5	20	2.47E-01	Without correction
190	AM_10581	A/A	12	21	NO_A/A	5	20	2.47E-01	Without correction
191	AM_14444	A/G	5	20	NO_A/G	12	21	2.47E-01	Without correction
192	AM_12778	NO_C/T	7	25	C/T	10	16	2.47E-01	Without correction
193	AM_10603	A/G	7	25	NO_A/G	10	16	2.47E-01	Without correction
194	AM_10035	G/T	7	25	NO_G/T	10	16	2.47E-01	Without correction
195	AM_13393	C/T	10	16	NO_C/T	7	25	2.47E-01	Without correction
196	AM_12092	NO_T/T	10	16	T/T	7	25	2.47E-01	Without correction
197	AM_12031	C/T	11	18	NO_C/T	6	23	2.48E-01	Without correction
198	AM_11500	NO_A/G	11	18	A/G	6	23	2.48E-01	Without correction
199	AM_15200	NO_T/T	16	32	T/T	1	9	2.53E-01	Without correction
200	AM_13464	NO_A/A	16	32	A/A	1	9	2.53E-01	Without correction

Result no.	Probe Set ID	Category 1	ClassA (n), category 1	ClassB (n), category 1	Category 2	ClassA (n), category 2	ClassB (n), category 2	Fisher exact test p-value	Multiple-testing correction
201	AM_12767	NO_C/T	16	32	C/T	1	9	2.53E-01	Without correction
202	AM_10691	NO_C/T	16	32	C/T	1	9	2.53E-01	Without correction
203	AM_13634	G/G	11	19	NO_G/G	6	22	2.55E-01	Without correction
204	AM_12778	NO_C/C	11	19	C/C	6	22	2.55E-01	Without correction
205	AM_11317	NO_C/T	6	22	C/T	11	19	2.55E-01	Without correction
206	AM_10633	A/G	6	22	NO_A/G	11	19	2.55E-01	Without correction
207	AM_10023	NO_A/T	6	22	A/T	11	19	2.55E-01	Without correction
208	AM_14348	C/C	16	33	NO_C/C	1	8	2.58E-01	Without correction
209	AM_12261	NO_T/T	16	33	T/T	1	8	2.58E-01	Without correction
210	AM_13437	NO_C/C	12	22	C/C	5	19	2.60E-01	Without correction
211	AM_12058	NO_A/A	12	22	A/A	5	19	2.60E-01	Without correction
212	AM_12261	C/C	10	17	NO_C/C	7	24	2.60E-01	Without correction
213	AM_10221	C/C	10	17	NO_C/C	7	24	2.60E-01	Without correction
214	AM_13600	NO_G/G	7	24	G/G	10	17	2.60E-01	Without correction
215	AM_11611	NO_A/C	7	24	A/C	10	17	2.60E-01	Without correction
216	AM_10730	NO_A/C	7	24	A/C	10	17	2.60E-01	Without correction
217	AM_14662	NO_C/G	12	35	C/G	5	6	2.70E-01	Without correction
218	AM_14662	C/C	12	35	NO_C/C	5	6	2.70E-01	Without correction
219	AM_11835	G/G	12	35	NO_G/G	5	6	2.70E-01	Without correction
220	AM_14151	NO_G/G	5	6	G/G	12	35	2.70E-01	Without correction
221	AM_14814	A/A	1	0	NO_A/A	16	41	2.93E-01	Without correction
222	AM_14151	A/A	1	0	NO_A/A	16	41	2.93E-01	Without correction
223	AM_14111	C/C	1	0	NO_C/C	16	41	2.93E-01	Without correction
224	AM_13509	C/C	1	0	NO_C/C	16	41	2.93E-01	Without correction
225	AM_11564	G/G	1	0	NO_G/G	16	41	2.93E-01	Without correction
226	AM_11482	T/T	1	0	NO_T/T	16	41	2.93E-01	Without correction
227	AM_11318	A/A	1	0	NO_A/A	16	41	2.93E-01	Without correction
228	AM_11034	A/A	1	0	NO_A/A	16	41	2.93E-01	Without correction
229	AM_10726	T/T	1	0	NO_T/T	16	41	2.93E-01	Without correction
230	AM_10047	T/T	1	0	NO_T/T	16	41	2.93E-01	Without correction
231	AM_12316	NO_G/G	15	30	G/G	2	11	3.07E-01	Without correction
232	AM_14227	NO_T/T	12	34	T/T	5	7	3.07E-01	Without correction
233	AM_10035	NO_G/G	12	34	G/G	5	7	3.07E-01	Without correction
234	AM_15202	NO_T/T	17	36	T/T	0	5	3.08E-01	Without correction
235	AM_10540	NO_A/A	17	36	A/A	0	5	3.08E-01	Without correction
236	AM_15000	NO_T/T	17	37	T/T	0	4	3.10E-01	Without correction
237	AM_14871	NO_T/T	17	37	T/T	0	4	3.10E-01	Without correction
238	AM_14624	NO_T/T	17	37	T/T	0	4	3.10E-01	Without correction
239	AM_14347	NO_C/C	17	37	C/C	0	4	3.10E-01	Without correction
240	AM_12370	NO_T/T	17	37	T/T	0	4	3.10E-01	Without correction
241	AM_12092	NO_C/C	17	37	C/C	0	4	3.10E-01	Without correction
242	AM_12031	NO_T/T	17	37	T/T	0	4	3.10E-01	Without correction
243	AM_11834	NO_T/T	17	37	T/T	0	4	3.10E-01	Without correction
244	AM_11611	NO_C/C	17	37	C/C	0	4	3.10E-01	Without correction
245	AM_11047	NO_T/T	17	37	T/T	0	4	3.10E-01	Without correction
246	AM_10579	NO_C/C	17	37	C/C	0	4	3.10E-01	Without correction
247	AM_14856	NO_T/T	11	33	T/T	6	8	3.11E-01	Without correction
248	AM_13775	NO_A/A	11	33	A/A	6	8	3.11E-01	Without correction
249	AM_10913	NO_A/A	11	33	A/A	6	8	3.11E-01	Without correction
250	AM_13775	G/G	6	8	NO_G/G	11	33	3.11E-01	Without correction
251	AM_10020	C/T	6	8	NO_C/T	11	33	3.11E-01	Without correction
252	AM_10592	NO_A/A	11	32	A/A	6	9	3.34E-01	Without correction

Result no.	Probe Set ID	Category 1	ClassA (n), category 1	ClassB (n), category 1	Category 2	ClassA (n), category 2	ClassB (n), category 2	Fisher exact test p-value	Multiple-testing correction
253	AM_14528	C/T	6	9	NO_C/T	11	32	3.34E-01	Without correction
254	AM_14490	C/C	6	9	NO_C/C	11	32	3.34E-01	Without correction
255	AM_12108	NO_C/C	6	9	C/C	11	32	3.34E-01	Without correction
256	AM_12108	C/T	6	9	NO_C/T	11	32	3.34E-01	Without correction
257	AM_10020	NO_C/C	6	9	C/C	11	32	3.34E-01	Without correction
258	AM_14814	G/G	14	27	NO_G/G	3	14	3.42E-01	Without correction
259	AM_13741	NO_A/G	14	27	A/G	3	14	3.42E-01	Without correction
260	AM_13741	A/A	14	27	NO_A/A	3	14	3.42E-01	Without correction
261	AM_13364	A/A	14	27	NO_A/A	3	14	3.42E-01	Without correction
262	AM_12205	G/G	14	27	NO_G/G	3	14	3.42E-01	Without correction
263	AM_11376	NO_C/C	14	27	C/C	3	14	3.42E-01	Without correction
264	AM_13896	NO_C/C	14	38	C/C	3	3	3.45E-01	Without correction
265	AM_12385	NO_T/T	14	38	T/T	3	3	3.45E-01	Without correction
266	AM_12137	NO_C/C	14	38	C/C	3	3	3.45E-01	Without correction
267	AM_10270	NO_G/G	14	38	G/G	3	3	3.45E-01	Without correction
268	AM_13959	C/C	14	28	NO_C/C	3	13	3.47E-01	Without correction
269	AM_11734	NO_-/AGG	14	28	-/AGG	3	13	3.47E-01	Without correction
270	AM_10709	NO_C/T	14	28	C/T	3	13	3.47E-01	Without correction
271	AM_11254	NO_G/G	3	13	G/G	14	28	3.47E-01	Without correction
272	AM_11254	A/G	3	13	NO_A/G	14	28	3.47E-01	Without correction
273	AM_11938	A/G	7	11	NO_A/G	10	30	3.54E-01	Without correction
274	AM_10053	C/T	7	11	NO_C/T	10	30	3.54E-01	Without correction
275	AM_14146	A/A	13	25	NO_A/A	4	16	3.66E-01	Without correction
276	AM_11279	G/G	13	25	NO_G/G	4	16	3.66E-01	Without correction
277	AM_10579	NO_C/T	13	25	C/T	4	16	3.66E-01	Without correction
278	AM_10498	C/C	13	25	NO_C/C	4	16	3.66E-01	Without correction
279	AM_12206	G/G	4	16	NO_G/G	13	25	3.66E-01	Without correction
280	AM_13148	NO_C/T	9	28	C/T	8	13	3.69E-01	Without correction
281	AM_11135	C/G	8	13	NO_C/G	9	28	3.69E-01	Without correction
282	AM_10724	A/G	8	13	NO_A/G	9	28	3.69E-01	Without correction
283	AM_14287	NO_G/G	4	15	G/G	13	26	3.77E-01	Without correction
284	AM_11734	AGG/AGG	13	26	NO_AGG/ AGG	4	15	3.77E-01	Without correction
285	AM_11084	NO_C/T	13	26	C/T	4	15	3.77E-01	Without correction
286	AM_14953	NO_A/G	8	26	A/G	9	15	3.80E-01	Without correction
287	AM_11299	NO_G/G	8	26	G/G	9	15	3.80E-01	Without correction
288	AM_11407	C/C	9	15	NO_C/C	8	26	3.80E-01	Without correction
289	AM_14386	NO_A/A	5	18	A/A	12	23	3.84E-01	Without correction
290	AM_13207	C/C	5	18	NO_C/C	12	23	3.84E-01	Without correction
291	AM_10152	NO_G/G	5	18	G/G	12	23	3.84E-01	Without correction
292	AM_15307	NO_A/A	12	23	A/A	5	18	3.84E-01	Without correction
293	AM_15000	NO_C/T	12	23	C/T	5	18	3.84E-01	Without correction
294	AM_14226	NO_T/T	12	23	T/T	5	18	3.84E-01	Without correction
295	AM_14118	NO_C/T	12	23	C/T	5	18	3.84E-01	Without correction
296	AM_14118	C/C	12	23	NO_C/C	5	18	3.84E-01	Without correction
297	AM_13817	T/T	12	23	NO_T/T	5	18	3.84E-01	Without correction
298	AM_13307	NO_A/G	12	23	A/G	5	18	3.84E-01	Without correction
299	AM_13298	NO_A/G	12	23	A/G	5	18	3.84E-01	Without correction
300	AM_11066	A/A	12	23	NO_A/A	5	18	3.84E-01	Without correction
301	AM_11022	NO_A/A	12	23	A/A	5	18	3.84E-01	Without correction
302	AM_10581	NO_A/G	12	23	A/G	5	18	3.84E-01	Without correction
303	AM_12857	C/T	8	14	NO_C/T	9	27	3.87E-01	Without correction

Result no.	Probe Set ID	Category 1	ClassA (n), category 1	ClassB (n), category 1	Category 2	ClassA (n), category 2	ClassB (n), category 2	Fisher exact test p-value	Multiple-testing correction
304	AM_12126	NO_C/C	9	27	C/C	8	14	3.87E-01	Without correction
305	AM_11609	NO_C/T	9	27	C/T	8	14	3.87E-01	Without correction
306	AM_15380	NO_C	6	21	C	11	20	3.87E-01	Without correction
307	AM_11299	A/G	6	21	NO_A/G	11	20	3.87E-01	Without correction
308	AM_14624	G/G	11	20	NO_G/G	6	21	3.87E-01	Without correction
309	AM_14363	NO_G/G	11	20	G/G	6	21	3.87E-01	Without correction
310	AM_13634	NO_A/G	11	20	A/G	6	21	3.87E-01	Without correction
311	AM_13439	G/T	11	20	NO_G/T	6	21	3.87E-01	Without correction
312	AM_12461	NO_C/G	11	20	C/G	6	21	3.87E-01	Without correction
313	AM_14953	G/G	8	25	NO_G/G	9	16	3.90E-01	Without correction
314	AM_13647	A/G	8	25	NO_A/G	9	16	3.90E-01	Without correction
315	AM_13635	C/G	8	25	NO_C/G	9	16	3.90E-01	Without correction
316	AM_13534	NO_A/A	8	25	A/A	9	16	3.90E-01	Without correction
317	AM_11376	NO_C/T	8	25	C/T	9	16	3.90E-01	Without correction
318	AM_10742	NO_G/G	8	25	G/G	9	16	3.90E-01	Without correction
319	AM_15285	NO_C/T	9	16	C/T	8	25	3.90E-01	Without correction
320	AM_14661	NO_A/T	9	16	A/T	8	25	3.90E-01	Without correction
321	AM_13903	NO_C/T	9	16	C/T	8	25	3.90E-01	Without correction
322	AM_13896	NO_C/G	9	16	C/G	8	25	3.90E-01	Without correction
323	AM_12247	C/G	9	16	NO_C/G	8	25	3.90E-01	Without correction
324	AM_11358	NO_G/G	9	16	G/G	8	25	3.90E-01	Without correction
325	AM_11358	A/G	9	16	NO_A/G	8	25	3.90E-01	Without correction
326	AM_11300	C/C	9	16	NO_C/C	8	25	3.90E-01	Without correction
327	AM_10724	NO_G/G	9	16	G/G	8	25	3.90E-01	Without correction
328	AM_12204	NO_A/G	7	23	A/G	10	18	3.90E-01	Without correction
329	AM_15010	NO_A/G	10	18	A/G	7	23	3.90E-01	Without correction
330	AM_11407	NO_C/G	10	18	C/G	7	23	3.90E-01	Without correction
331	AM_11023	NO_C/T	10	18	C/T	7	23	3.90E-01	Without correction
332	AM_10675	NO_C/T	10	18	C/T	7	23	3.90E-01	Without correction
333	AM_13727	NO_C/C	11	21	C/C	6	20	3.97E-01	Without correction
334	AM_12385	NO_C/T	11	21	C/T	6	20	3.97E-01	Without correction
335	AM_12160	T/T	11	21	NO_T/T	6	20	3.97E-01	Without correction
336	AM_11719	NO_A/A	11	21	A/A	6	20	3.97E-01	Without correction
337	AM_11719	A/G	11	21	NO_A/G	6	20	3.97E-01	Without correction
338	AM_11415	A/A	11	21	NO_A/A	6	20	3.97E-01	Without correction
339	AM_10714	NO_T/T	11	21	T/T	6	20	3.97E-01	Without correction
340	AM_13534	A/C	6	20	NO_A/C	11	21	3.97E-01	Without correction
341	AM_12327	T/T	6	20	NO_T/T	11	21	3.97E-01	Without correction
342	AM_14862	NO_C/T	14	37	C/T	3	4	4.07E-01	Without correction
343	AM_14862	C/C	14	37	NO_C/C	3	4	4.07E-01	Without correction
344	AM_14227	NO_C/C	14	37	C/C	3	4	4.07E-01	Without correction
345	AM_14132	NO_C/C	14	37	C/C	3	4	4.07E-01	Without correction
346	AM_13346	NO_T/T	14	37	T/T	3	4	4.07E-01	Without correction
347	AM_13148	NO_T/T	14	37	T/T	3	4	4.07E-01	Without correction
348	AM_12170	T/T	3	4	NO_T/T	14	37	4.07E-01	Without correction
349	AM_12058	NO_G/G	14	37	G/G	3	4	4.07E-01	Without correction
350	AM_14519	NO_A/G	16	34	A/G	1	7	4.15E-01	Without correction
351	AM_14519	G/G	16	34	NO_G/G	1	7	4.15E-01	Without correction
352	AM_14287	G/T	1	7	NO_G/T	16	34	4.15E-01	Without correction
353	AM_12947	NO_T/T	16	34	T/T	1	7	4.15E-01	Without correction
354	AM_12850	NO_A/G	16	34	A/G	1	7	4.15E-01	Without correction
355	AM_12850	G/G	16	34	NO_G/G	1	7	4.15E-01	Without correction

Result no.	Probe Set ID	Category 1	ClassA (n), category 1	ClassB (n), category 1	Category 2	ClassA (n), category 2	ClassB (n), category 2	Fisher exact test p-value	Multiple-testing correction
356	AM_12322	NO_T/T	16	34	T/T	1	7	4.15E-01	Without correction
357	AM_12229	NO_A/A	16	34	A/A	1	7	4.15E-01	Without correction
358	AM_12172	NO_G/G	16	34	G/G	1	7	4.15E-01	Without correction
359	AM_11688	NO_C/T	16	34	C/T	1	7	4.15E-01	Without correction
360	AM_11688	C/C	16	34	NO_C/C	1	7	4.15E-01	Without correction
361	AM_14592	NO_T/T	13	36	T/T	4	5	4.26E-01	Without correction
362	AM_13323	NO_A/A	13	36	A/A	4	5	4.26E-01	Without correction
363	AM_10440	NO_G/G	13	36	G/G	4	5	4.26E-01	Without correction
364	AM_10135	NO_A/G	13	36	A/G	4	5	4.26E-01	Without correction
365	AM_15047	NO_C/C	13	35	C/C	4	6	4.58E-01	Without correction
366	AM_11525	NO_A/C	13	35	A/C	4	6	4.58E-01	Without correction
367	AM_10135	G/G	13	35	NO_G/G	4	6	4.58E-01	Without correction
368	AM_14401	T/T	4	6	NO_T/T	13	35	4.58E-01	Without correction
369	AM_14151	A/G	4	6	NO_A/G	13	35	4.58E-01	Without correction
370	AM_11925	NO_A/A	4	6	A/A	13	35	4.58E-01	Without correction
371	AM_11925	A/G	4	6	NO_A/G	13	35	4.58E-01	Without correction
372	AM_14592	NO_G/G	15	31	G/G	2	10	4.78E-01	Without correction
373	AM_12767	T/T	15	31	NO_T/T	2	10	4.78E-01	Without correction
374	AM_11205	NO_T/T	15	31	T/T	2	10	4.78E-01	Without correction
375	AM_10913	G/G	2	10	NO_G/G	15	31	4.78E-01	Without correction
376	AM_10503	NO_T/T	15	31	T/T	2	10	4.78E-01	Without correction
377	AM_13437	NO_A/A	15	32	A/A	2	9	4.80E-01	Without correction
378	AM_13400	NO_G/G	15	32	G/G	2	9	4.80E-01	Without correction
379	AM_12974	NO_G/G	15	32	G/G	2	9	4.80E-01	Without correction
380	AM_10333	NO_C/C	15	32	C/C	2	9	4.80E-01	Without correction
381	AM_11744	T/T	2	9	NO_T/T	15	32	4.80E-01	Without correction
382	AM_10807	C/C	2	9	NO_C/C	15	32	4.80E-01	Without correction
383	AM_11724	NO_C/T	12	33	C/T	5	8	4.94E-01	Without correction
384	AM_11724	T/T	12	33	NO_T/T	5	8	4.94E-01	Without correction
385	AM_15010	G/G	5	8	NO_G/G	12	33	4.94E-01	Without correction
386	AM_15390	NO_A/A	16	40	A/A	1	1	5.04E-01	Without correction
387	AM_14568	NO_T/T	16	40	T/T	1	1	5.04E-01	Without correction
388	AM_14553	NO_T/T	16	40	T/T	1	1	5.04E-01	Without correction
389	AM_13760	T/T	1	1	NO_T/T	16	40	5.04E-01	Without correction
390	AM_13324	NO_C/C	16	40	C/C	1	1	5.04E-01	Without correction
391	AM_12767	NO_C/C	16	40	C/C	1	1	5.04E-01	Without correction
392	AM_11809	NO_C/C	16	40	C/C	1	1	5.04E-01	Without correction
393	AM_11084	NO_T/T	16	40	T/T	1	1	5.04E-01	Without correction
394	AM_11066	NO_G/G	16	40	G/G	1	1	5.04E-01	Without correction
395	AM_10709	NO_C/C	16	40	C/C	1	1	5.04E-01	Without correction
396	AM_10666	G/G	1	1	NO_G/G	16	40	5.04E-01	Without correction
397	AM_10498	NO_A/A	16	40	A/A	1	1	5.04E-01	Without correction
398	AM_10243	NO_A/A	16	40	A/A	1	1	5.04E-01	Without correction
399	AM_10033	NO_A/A	16	40	A/A	1	1	5.04E-01	Without correction
400	AM_13364	NO_A/C	14	29	A/C	3	12	5.14E-01	Without correction
401	AM_12247	NO_C/C	14	29	C/C	3	12	5.14E-01	Without correction
402	AM_12205	NO_A/G	14	29	A/G	3	12	5.14E-01	Without correction
403	AM_11564	NO_A/G	14	29	A/G	3	12	5.14E-01	Without correction
404	AM_11317	NO_C/C	14	29	C/C	3	12	5.14E-01	Without correction
405	AM_11279	NO_A/G	14	29	A/G	3	12	5.14E-01	Without correction
406	AM_10030	NO_C/G	14	29	C/G	3	12	5.14E-01	Without correction
407	AM_13841	NO_A/G	11	31	A/G	6	10	5.20E-01	Without correction

Result no.	Probe Set ID	Category 1	ClassA (n), category 1	ClassB (n), category 1	Category 2	ClassA (n), category 2	ClassB (n), category 2	Fisher exact test p-value	Multiple-testing correction
408	AM_13439	NO_T/T	11	31	T/T	6	10	5.20E-01	Without correction
409	AM_13307	NO_G/G	11	31	G/G	6	10	5.20E-01	Without correction
410	AM_13298	NO_G/G	11	31	G/G	6	10	5.20E-01	Without correction
411	AM_10673	G/G	11	31	NO_G/G	6	10	5.20E-01	Without correction
412	AM_10333	NO_G/G	11	31	G/G	6	10	5.20E-01	Without correction
413	AM_14681	A/T	6	10	NO_A/T	11	31	5.20E-01	Without correction
414	AM_13959	NO_C/T	14	30	C/T	3	11	5.23E-01	Without correction
415	AM_13400	NO_A/A	14	30	A/A	3	11	5.23E-01	Without correction
416	AM_12174	NO_G/G	14	30	G/G	3	11	5.23E-01	Without correction
417	AM_10454	NO_A/G	14	30	A/G	3	11	5.23E-01	Without correction
418	AM_10454	G/G	14	30	NO_G/G	3	11	5.23E-01	Without correction
419	AM_10221	NO_A/A	14	30	A/A	3	11	5.23E-01	Without correction
420	AM_12454	NO_A/G	10	29	A/G	7	12	5.40E-01	Without correction
421	AM_12454	A/A	10	29	NO_A/A	7	12	5.40E-01	Without correction
422	AM_11482	C/T	7	12	NO_C/T	10	29	5.40E-01	Without correction
423	AM_11168	G/G	7	12	NO_G/G	10	29	5.40E-01	Without correction
424	AM_10053	NO_C/C	7	12	C/C	10	29	5.40E-01	Without correction
425	AM_13903	NO_C/C	11	30	C/C	6	11	5.40E-01	Without correction
426	AM_13841	G/G	11	30	NO_G/G	6	11	5.40E-01	Without correction
427	AM_13800	NO_A/G	11	30	A/G	6	11	5.40E-01	Without correction
428	AM_13377	NO_C/T	11	30	C/T	6	11	5.40E-01	Without correction
429	AM_12316	NO_C/C	11	30	C/C	6	11	5.40E-01	Without correction
430	AM_14368	NO_GAT/GAT	6	11	GAT/GAT	11	30	5.40E-01	Without correction
431	AM_14368	-/GAT	6	11	NO_-/GAT	11	30	5.40E-01	Without correction
432	AM_12140	C/T	6	11	NO_C/T	11	30	5.40E-01	Without correction
433	AM_10709	T/T	13	27	NO_T/T	4	14	5.40E-01	Without correction
434	AM_10490	A/A	13	27	NO_A/A	4	14	5.40E-01	Without correction
435	AM_14528	NO_C/C	17	38	C/C	0	3	5.48E-01	Without correction
436	AM_14386	NO_G/G	17	38	G/G	0	3	5.48E-01	Without correction
437	AM_13894	NO_G/G	17	38	G/G	0	3	5.48E-01	Without correction
438	AM_13759	NO_C/C	17	38	C/C	0	3	5.48E-01	Without correction
439	AM_13463	NO_G/G	17	38	G/G	0	3	5.48E-01	Without correction
440	AM_13140	NO_G/G	17	38	G/G	0	3	5.48E-01	Without correction
441	AM_11609	NO_T/T	17	38	T/T	0	3	5.48E-01	Without correction
442	AM_11364	NO_A/A	17	38	A/A	0	3	5.48E-01	Without correction
443	AM_11131	NO_C/C	17	38	C/C	0	3	5.48E-01	Without correction
444	AM_11078	NO_T/T	17	38	T/T	0	3	5.48E-01	Without correction
445	AM_10903	NO_G/G	17	38	G/G	0	3	5.48E-01	Without correction
446	AM_10673	NO_T/T	17	38	T/T	0	3	5.48E-01	Without correction
447	AM_12589	NO_G/G	10	28	G/G	7	13	5.51E-01	Without correction
448	AM_10807	NO_T/T	10	28	T/T	7	13	5.51E-01	Without correction
449	AM_11730	A/G	7	13	NO_A/G	10	28	5.51E-01	Without correction
450	AM_11005	A/G	7	13	NO_A/G	10	28	5.51E-01	Without correction
451	AM_12370	C/C	12	24	NO_C/C	5	17	5.54E-01	Without correction
452	AM_10389	NO_G/G	12	24	G/G	5	17	5.54E-01	Without correction
453	AM_10183	NO_C/C	12	24	C/C	5	17	5.54E-01	Without correction
454	AM_10023	A/A	5	17	NO_A/A	12	24	5.54E-01	Without correction
455	AM_13175	T/T	8	15	NO_T/T	9	26	5.59E-01	Without correction
456	AM_11135	NO_C/C	8	15	C/C	9	26	5.59E-01	Without correction
458	AM_14871	NO_C/T	12	25	C/T	5	16	5.60E-01	Without correction
459	AM_13817	NO_C/T	12	25	C/T	5	16	5.60E-01	Without correction
460	AM_11416	NO_A/A	12	25	A/A	5	16	5.60E-01	Without correction

Result no.	Probe Set ID	Category 1	ClassA (n), category 1	ClassB (n), category 1	Category 2	ClassA (n), category 2	ClassB (n), category 2	Fisher exact test p-value	Multiple-testing correction
461	AM_11084	C/C	12	25	NO_C/C	5	16	5.60E-01	Without correction
462	AM_13463	NO_A/A	5	16	A/A	12	25	5.60E-01	Without correction
463	AM_12461	C/C	5	16	NO_C/C	12	25	5.60E-01	Without correction
464	AM_10473	NO_T/T	5	16	T/T	12	25	5.60E-01	Without correction
465	AM_10152	A/G	5	16	NO_A/G	12	25	5.60E-01	Without correction
466	AM_15047	A/C	8	24	NO_A/C	9	17	5.63E-01	Without correction
467	AM_14913	NO_A/C	8	24	A/C	9	17	5.63E-01	Without correction
468	AM_14163	C/T	8	24	NO_C/T	9	17	5.63E-01	Without correction
469	AM_13337	A/G	8	24	NO_A/G	9	17	5.63E-01	Without correction
470	AM_12327	NO_C/T	8	24	C/T	9	17	5.63E-01	Without correction
471	AM_11744	NO_A/A	8	24	A/A	9	17	5.63E-01	Without correction
472	AM_10540	NO_T/T	8	24	T/T	9	17	5.63E-01	Without correction
473	AM_10031	G/T	8	24	NO_G/T	9	17	5.63E-01	Without correction
474	AM_15050	C/T	9	17	NO_C/T	8	24	5.63E-01	Without correction
475	AM_11809	NO_G/G	9	17	G/G	8	24	5.63E-01	Without correction
476	AM_10270	G/T	9	17	NO_G/T	8	24	5.63E-01	Without correction
477	AM_10206	T/T	9	17	NO_T/T	8	24	5.63E-01	Without correction
478	AM_15349	NO_A/G	11	22	A/G	6	19	5.64E-01	Without correction
479	AM_14896	A/A	11	22	NO_A/A	6	19	5.64E-01	Without correction
480	AM_13393	NO_C/C	11	22	C/C	6	19	5.64E-01	Without correction
481	AM_12031	NO_C/C	11	22	C/C	6	19	5.64E-01	Without correction
482	AM_11364	A/G	11	22	NO_A/G	6	19	5.64E-01	Without correction
483	AM_10742	NO_G/T	11	22	G/T	6	19	5.64E-01	Without correction
484	AM_10389	A/G	11	22	NO_A/G	6	19	5.64E-01	Without correction
485	AM_13323	A/T	6	19	NO_A/T	11	22	5.64E-01	Without correction
486	AM_11804	C/G	6	19	NO_C/G	11	22	5.64E-01	Without correction
487	AM_11131	NO_C/T	6	19	C/T	11	22	5.64E-01	Without correction
488	AM_10726	C/T	6	19	NO_C/T	11	22	5.64E-01	Without correction
489	AM_14196	NO_T/T	10	19	T/T	7	22	5.65E-01	Without correction
490	AM_13894	NO_C/T	7	22	C/T	10	19	5.65E-01	Without correction
491	AM_11300	NO_C/T	10	19	C/T	7	22	5.65E-01	Without correction
492	AM_11128	NO_A/G	7	22	A/G	10	19	5.65E-01	Without correction
493	AM_11128	A/A	7	22	NO_A/A	10	19	5.65E-01	Without correction
494	AM_15202	G/G	10	20	NO_G/G	7	21	5.70E-01	Without correction
495	AM_12137	NO_T/T	10	20	T/T	7	21	5.70E-01	Without correction
496	AM_11968	NO_C/C	10	20	C/C	7	21	5.70E-01	Without correction
497	AM_11938	NO_A/A	10	20	A/A	7	21	5.70E-01	Without correction
498	AM_11875	NO_G/G	10	20	G/G	7	21	5.70E-01	Without correction
499	AM_11034	NO_G/G	10	20	G/G	7	21	5.70E-01	Without correction
500	AM_13166	NO_G/G	7	21	G/G	10	20	5.70E-01	Without correction
501	AM_11310	G/T	7	21	NO_G/T	10	20	5.70E-01	Without correction
502	AM_11253	NO_T/T	7	21	T/T	10	20	5.70E-01	Without correction
503	AM_10697	NO_C/C	7	21	C/C	10	20	5.70E-01	Without correction
504	AM_10503	NO_C/T	7	21	C/T	10	20	5.70E-01	Without correction
505	AM_14196	NO_A/A	15	39	A/A	2	2	5.73E-01	Without correction
506	AM_14163	NO_T/T	15	39	T/T	2	2	5.73E-01	Without correction
507	AM_13600	NO_A/A	15	39	A/A	2	2	5.73E-01	Without correction
508	AM_12590	NO_G/G	15	39	G/G	2	2	5.73E-01	Without correction
509	AM_12160	NO_C/C	15	39	C/C	2	2	5.73E-01	Without correction
510	AM_11296	NO_C/C	15	39	C/C	2	2	5.73E-01	Without correction
511	AM_10698	NO_C/C	15	39	C/C	2	2	5.73E-01	Without correction
512	AM_10671	NO_T/T	15	39	T/T	2	2	5.73E-01	Without correction

Result no.	Probe Set ID	Category 1	ClassA (n), category 1	ClassB (n), category 1	Category 2	ClassA (n), category 2	ClassB (n), category 2	Fisher exact test p-value	Multiple-testing correction
513	AM_14856	C/T	8	23	NO_C/T	9	18	5.74E-01	Without correction
514	AM_13979	T/T	8	23	NO_T/T	9	18	5.74E-01	Without correction
515	AM_13324	A/C	8	23	NO_A/C	9	18	5.74E-01	Without correction
516	AM_13175	NO_C/T	9	18	C/T	8	23	5.74E-01	Without correction
517	AM_12058	A/G	9	18	NO_A/G	8	23	5.74E-01	Without correction
518	AM_11730	NO_G/G	9	18	G/G	8	23	5.74E-01	Without correction
519	AM_11416	NO_A/G	9	18	A/G	8	23	5.74E-01	Without correction
520	AM_12857	NO_T/T	11	23	T/T	6	18	5.75E-01	Without correction
521	AM_12590	NO_C/G	11	23	C/G	6	18	5.75E-01	Without correction
522	AM_13346	C/T	6	18	NO_C/T	11	23	5.75E-01	Without correction
523	AM_13166	A/G	6	18	NO_A/G	11	23	5.75E-01	Without correction
524	AM_13140	NO_C/G	6	18	C/G	11	23	5.75E-01	Without correction
525	AM_10714	G/T	6	18	NO_G/T	11	23	5.75E-01	Without correction
526	AM_14705	NO_A/A	15	38	A/A	2	3	6.24E-01	Without correction
527	AM_14336	NO_G/G	15	38	G/G	2	3	6.24E-01	Without correction
528	AM_12974	NO_A/G	15	38	A/G	2	3	6.24E-01	Without correction
529	AM_12165	NO_T/T	15	38	T/T	2	3	6.24E-01	Without correction
530	AM_14216	NO_A/A	16	36	A/A	1	5	6.60E-01	Without correction
531	AM_11415	NO_G/G	16	36	G/G	1	5	6.60E-01	Without correction
532	AM_10023	NO_T/T	16	36	T/T	1	5	6.60E-01	Without correction
533	AM_15086	NO_C/C	16	35	C/C	1	6	6.61E-01	Without correction
534	AM_14348	NO_C/T	16	35	C/T	1	6	6.61E-01	Without correction
535	AM_14226	NO_C/C	16	35	C/C	1	6	6.61E-01	Without correction
536	AM_13647	NO_A/A	16	35	A/A	1	6	6.61E-01	Without correction
537	AM_13393	NO_T/T	16	35	T/T	1	6	6.61E-01	Without correction
538	AM_12206	NO_C/C	16	35	C/C	1	6	6.61E-01	Without correction
539	AM_11968	NO_T/T	16	35	T/T	1	6	6.61E-01	Without correction
540	AM_11710	NO_T/T	16	35	T/T	1	6	6.61E-01	Without correction
541	AM_10030	NO_G/C	16	35	G/C	1	6	6.61E-01	Without correction
542	AM_15306	NO_A/G	14	36	A/G	3	5	6.81E-01	Without correction
543	AM_15306	A/A	14	36	NO_A/A	3	5	6.81E-01	Without correction
544	AM_13903	T/T	3	5	NO_T/T	14	36	6.81E-01	Without correction
545	AM_13163	NO_T/T	14	36	T/T	3	5	6.81E-01	Without correction
546	AM_10920	NO_C/C	14	36	C/C	3	5	6.81E-01	Without correction
547	AM_10899	NO_A/A	14	36	A/A	3	5	6.81E-01	Without correction
548	AM_15050	NO_C/C	15	33	C/C	2	8	7.07E-01	Without correction
549	AM_14490	NO_T/T	15	33	T/T	2	8	7.07E-01	Without correction
550	AM_14363	NO_A/A	15	33	A/A	2	8	7.07E-01	Without correction
551	AM_12656	NO_G/G	15	33	G/G	2	8	7.07E-01	Without correction
552	AM_11875	NO_A/A	15	33	A/A	2	8	7.07E-01	Without correction
553	AM_11499	NO_C/C	15	33	C/C	2	8	7.07E-01	Without correction
554	AM_11130	NO_C/T	15	33	C/T	2	8	7.07E-01	Without correction
555	AM_11130	C/C	15	33	NO_C/C	2	8	7.07E-01	Without correction
556	AM_10782	NO_T/T	2	8	T/T	15	33	7.07E-01	Without correction
557	AM_10782	-/T	2	8	NO_-/T	15	33	7.07E-01	Without correction
558	AM_15349	NO_G/G	13	34	G/G	4	7	7.15E-01	Without correction
559	AM_15226	NO_C/T	13	34	C/T	4	7	7.15E-01	Without correction
560	AM_14609	NO_T/T	13	34	T/T	4	7	7.15E-01	Without correction
561	AM_13603	NO_T/T	13	34	T/T	4	7	7.15E-01	Without correction
562	AM_11525	C/C	13	34	NO_C/C	4	7	7.15E-01	Without correction
563	AM_10890	NO_C/G	13	34	C/G	4	7	7.15E-01	Without correction
564	AM_10890	C/C	13	34	NO_C/C	4	7	7.15E-01	Without correction

Result no.	Probe Set ID	Category 1	ClassA (n), category 1	ClassB (n), category 1	Category 2	ClassA (n), category 2	ClassB (n), category 2	Fisher exact test p-value	Multiple-testing correction
565	AM_11078	C/T	4	8	NO_C/T	13	33	7.33E-01	Without correction
566	AM_15226	T/T	13	33	NO_T/T	4	8	7.33E-01	Without correction
567	AM_12982	NO_A/C	13	33	A/C	4	8	7.33E-01	Without correction
568	AM_12982	C/C	13	33	NO_C/C	4	8	7.33E-01	Without correction
569	AM_12782	NO_A/G	13	33	A/G	4	8	7.33E-01	Without correction
570	AM_12782	G/G	13	33	NO_G/G	4	8	7.33E-01	Without correction
571	AM_10947	NO_C/T	13	33	C/T	4	8	7.33E-01	Without correction
572	AM_10839	NO_G/T	13	33	G/T	4	8	7.33E-01	Without correction
573	AM_10839	G/G	13	33	NO_G/G	4	8	7.33E-01	Without correction
574	AM_10711	NO_C/T	13	33	C/T	4	8	7.33E-01	Without correction
575	AM_14856	NO_C/C	14	31	C/C	3	10	7.36E-01	Without correction
576	AM_14471	G/G	14	31	NO_G/G	3	10	7.36E-01	Without correction
577	AM_11804	NO_C/C	14	31	C/C	3	10	7.36E-01	Without correction
578	AM_10977	NO_A/A	14	31	A/A	3	10	7.36E-01	Without correction
579	AM_15285	C/C	3	10	NO_C/C	14	31	7.36E-01	Without correction
580	AM_12217	NO_A/A	3	10	A/A	14	31	7.36E-01	Without correction
581	AM_12217	A/G	3	10	NO_A/G	14	31	7.36E-01	Without correction
582	AM_11317	T/T	3	10	NO_T/T	14	31	7.36E-01	Without correction
583	AM_10473	C/T	3	10	NO_C/T	14	31	7.36E-01	Without correction
584	AM_14913	A/A	5	9	NO_A/A	12	32	7.37E-01	Without correction
585	AM_12216	G/G	5	9	NO_G/G	12	32	7.37E-01	Without correction
586	AM_12208	A/A	5	9	NO_A/A	12	32	7.37E-01	Without correction
587	AM_12147	NO_T/T	12	32	T/T	5	9	7.37E-01	Without correction
588	AM_11913	NO_A/G	12	32	A/G	5	9	7.37E-01	Without correction
589	AM_11913	G/G	12	32	NO_G/G	5	9	7.37E-01	Without correction
590	AM_11554	C/T	5	9	NO_C/T	12	32	7.37E-01	Without correction
591	AM_11205	G/G	5	9	NO_G/G	12	32	7.37E-01	Without correction
592	AM_11088	NO_A/G	12	32	A/G	5	9	7.37E-01	Without correction
593	AM_10698	T/T	12	32	NO_T/T	5	9	7.37E-01	Without correction
594	AM_10035	NO_T/T	12	32	T/T	5	9	7.37E-01	Without correction
595	AM_11088	G/G	12	31	NO_G/G	5	10	7.47E-01	Without correction
596	AM_10010	NO_C/C	12	31	C/C	5	10	7.47E-01	Without correction
597	AM_15010	NO_A/A	12	31	A/A	5	10	7.47E-01	Without correction
598	AM_13759	NO_C/T	12	31	C/T	5	10	7.47E-01	Without correction
599	AM_12204	A/A	5	10	NO_A/A	12	31	7.47E-01	Without correction
600	AM_11054	G/G	5	10	NO_G/G	12	31	7.47E-01	Without correction
601	AM_11023	NO_T/T	13	28	T/T	4	13	7.53E-01	Without correction
602	AM_10490	NO_A/G	13	28	A/G	4	13	7.53E-01	Without correction
603	AM_10221	NO_A/C	13	28	A/C	4	13	7.53E-01	Without correction
604	AM_15085	T/T	13	28	NO_T/T	4	13	7.53E-01	Without correction
605	AM_13011	NO_A/A	13	28	A/A	4	13	7.53E-01	Without correction
606	AM_12974	A/A	13	28	NO_A/A	4	13	7.53E-01	Without correction
607	AM_12214	NO_C/C	13	28	C/C	4	13	7.53E-01	Without correction
608	AM_12214	C/T	13	28	NO_C/T	4	13	7.53E-01	Without correction
609	AM_14322	NO_G/G	4	13	G/G	13	28	7.53E-01	Without correction
610	AM_12257	NO_A/G	13	29	A/G	4	12	7.55E-01	Without correction
611	AM_12257	G/G	13	29	NO_G/G	4	12	7.55E-01	Without correction
612	AM_12207	NO_C/G	13	29	C/G	4	12	7.55E-01	Without correction
613	AM_12207	C/C	13	29	NO_C/C	4	12	7.55E-01	Without correction
614	AM_11564	A/A	13	29	NO_A/A	4	12	7.55E-01	Without correction
615	AM_10671	NO_C/T	13	29	C/T	4	12	7.55E-01	Without correction
616	AM_10047	NO_C/T	13	29	C/T	4	12	7.55E-01	Without correction

Result no.	Probe Set ID	Category 1	ClassA (n), category 1	ClassB (n), category 1	Category 2	ClassA (n), category 2	ClassB (n), category 2	Fisher exact test p-value	Multiple-testing correction
617	AM_10390	T/T	4	12	NO_T/T	13	29	7.55E-01	Without correction
618	AM_15200	C/C	4	12	NO_C/C	13	29	7.55E-01	Without correction
619	AM_14322	A/G	4	12	NO_A/G	13	29	7.55E-01	Without correction
620	AM_14296	NO_C/C	11	29	C/C	6	12	7.58E-01	Without correction
621	AM_13800	G/G	11	29	NO_G/G	6	12	7.58E-01	Without correction
622	AM_13253	NO_A/G	11	29	A/G	6	12	7.58E-01	Without correction
623	AM_15407	G	6	12	NO_G	11	29	7.58E-01	Without correction
624	AM_14528	NO_T/T	6	12	T/T	11	29	7.58E-01	Without correction
625	AM_12229	G/G	6	12	NO_G/G	11	29	7.58E-01	Without correction
626	AM_14896	NO_A/G	12	26	A/G	5	15	7.64E-01	Without correction
627	AM_13326	NO_G/G	12	26	G/G	5	15	7.64E-01	Without correction
628	AM_11710	C/T	12	26	NO_C/T	5	15	7.64E-01	Without correction
629	AM_11415	NO_A/G	12	26	A/G	5	15	7.64E-01	Without correction
630	AM_11203	NO_T/T	12	26	T/T	5	15	7.64E-01	Without correction
631	AM_11026	NO_G/T	12	26	G/T	5	15	7.64E-01	Without correction
632	AM_11026	T/T	12	26	NO_T/T	5	15	7.64E-01	Without correction
633	AM_10920	NO_T/T	12	26	T/T	5	15	7.64E-01	Without correction
634	AM_14386	A/G	5	15	NO_A/G	12	26	7.64E-01	Without correction
635	AM_13894	C/C	5	15	NO_C/C	12	26	7.64E-01	Without correction
636	AM_13536	A/G	5	15	NO_A/G	12	26	7.64E-01	Without correction
637	AM_15210	NO_G/G	10	27	G/G	7	14	7.65E-01	Without correction
638	AM_14609	C/T	10	27	NO_C/T	7	14	7.65E-01	Without correction
639	AM_14321	NO_G/G	10	27	G/G	7	14	7.65E-01	Without correction
640	AM_12589	A/G	10	27	NO_A/G	7	14	7.65E-01	Without correction
641	AM_12322	NO_C/C	10	27	C/C	7	14	7.65E-01	Without correction
642	AM_13192	C/C	7	14	NO_C/C	10	27	7.65E-01	Without correction
643	AM_12656	A/A	7	14	NO_A/A	10	27	7.65E-01	Without correction
644	AM_14955	NO_C/C	9	25	C/C	8	16	7.70E-01	Without correction
645	AM_11310	NO_T/T	9	25	T/T	8	16	7.70E-01	Without correction
646	AM_10048	C/C	9	25	NO_C/C	8	16	7.70E-01	Without correction
647	AM_10033	G/G	9	25	NO_G/G	8	16	7.70E-01	Without correction
648	AM_15095	A/C	8	16	NO_A/C	9	25	7.70E-01	Without correction
649	AM_13011	A/G	8	16	NO_A/G	9	25	7.70E-01	Without correction
650	AM_11809	C/G	8	16	NO_C/G	9	25	7.70E-01	Without correction
651	AM_11168	NO_A/G	8	16	A/G	9	25	7.70E-01	Without correction
652	AM_11047	NO_C/C	8	16	C/C	9	25	7.70E-01	Without correction
653	AM_13536	NO_G/G	6	17	G/G	11	24	7.72E-01	Without correction
654	AM_12174	NO_A/G	6	17	A/G	11	24	7.72E-01	Without correction
655	AM_10697	C/T	6	17	NO_C/T	11	24	7.72E-01	Without correction
656	AM_14624	NO_G/T	11	24	G/T	6	17	7.72E-01	Without correction
657	AM_13979	NO_C/T	11	24	C/T	6	17	7.72E-01	Without correction
658	AM_12726	NO_C/C	11	24	C/C	6	17	7.72E-01	Without correction
659	AM_12170	NO_C/T	11	24	C/T	6	17	7.72E-01	Without correction
660	AM_12169	NO_C/C	11	24	C/C	6	17	7.72E-01	Without correction
661	AM_11499	A/C	11	24	NO_A/C	6	17	7.72E-01	Without correction
662	AM_11487	C/C	11	24	NO_C/C	6	17	7.72E-01	Without correction
663	AM_11203	A/T	11	24	NO_A/T	6	17	7.72E-01	Without correction
664	AM_10206	NO_C/T	11	24	C/T	6	17	7.72E-01	Without correction
665	AM_14518	NO_A/G	10	26	A/G	7	15	7.73E-01	Without correction
666	AM_14163	NO_C/C	10	26	C/C	7	15	7.73E-01	Without correction
667	AM_11474	NO_C/G	10	26	C/G	7	15	7.73E-01	Without correction
668	AM_11204	NO_A/G	10	26	A/G	7	15	7.73E-01	Without correction

Result no.	Probe Set ID	Category 1	ClassA (n), category 1	ClassB (n), category 1	Category 2	ClassA (n), category 2	ClassB (n), category 2	Fisher exact test p-value	Multiple-testing correction
669	AM_11204	G/G	10	26	NO_G/G	7	15	7.73E-01	Without correction
670	AM_10977	NO_A/C	10	26	A/C	7	15	7.73E-01	Without correction
671	AM_10033	NO_A/G	10	26	A/G	7	15	7.73E-01	Without correction
672	AM_15349	A/A	7	15	NO_A/A	10	26	7.73E-01	Without correction
673	AM_14111	NO_T/T	7	15	T/T	10	26	7.73E-01	Without correction
674	AM_12172	C/C	7	15	NO_C/C	10	26	7.73E-01	Without correction
675	AM_11912	C/T	7	15	NO_C/T	10	26	7.73E-01	Without correction
676	AM_10666	NO_A/A	7	15	A/A	10	26	7.73E-01	Without correction
677	AM_10113	NO_A/A	7	15	A/A	10	26	7.73E-01	Without correction
678	AM_10113	A/C	7	15	NO_A/C	10	26	7.73E-01	Without correction
679	AM_14336	NO_A/A	10	21	A/A	7	20	7.73E-01	Without correction
680	AM_13326	C/G	10	21	NO_C/G	7	20	7.73E-01	Without correction
681	AM_11611	A/A	7	20	NO_A/A	10	21	7.73E-01	Without correction
682	AM_13324	NO_A/A	9	24	A/A	8	17	7.75E-01	Without correction
683	AM_11609	C/C	9	24	NO_C/C	8	17	7.75E-01	Without correction
684	AM_15407	NO_A	8	17	A	9	24	7.75E-01	Without correction
685	AM_14681	NO_T/T	8	17	T/T	9	24	7.75E-01	Without correction
686	AM_14490	NO_C/T	8	17	C/T	9	24	7.75E-01	Without correction
687	AM_14196	A/T	8	17	NO_A/T	9	24	7.75E-01	Without correction
688	AM_11005	NO_G/G	8	17	G/G	9	24	7.75E-01	Without correction
689	AM_14955	NO_C/T	9	19	C/T	8	22	7.75E-01	Without correction
690	AM_12316	C/G	9	19	NO_C/G	8	22	7.75E-01	Without correction
691	AM_15094	NO_G/G	8	22	G/G	9	19	7.75E-01	Without correction
692	AM_12172	NO_C/G	8	22	C/G	9	19	7.75E-01	Without correction
693	AM_12169	NO_C/G	8	22	C/G	9	19	7.75E-01	Without correction
694	AM_10010	C/T	8	22	NO_C/T	9	19	7.75E-01	Without correction
695	AM_14592	G/T	10	22	NO_G/T	7	19	7.78E-01	Without correction
696	AM_14556	C/T	10	22	NO_C/T	7	19	7.78E-01	Without correction
697	AM_14216	G/G	10	22	NO_G/G	7	19	7.78E-01	Without correction
698	AM_14321	A/G	7	19	NO_A/G	10	22	7.78E-01	Without correction
699	AM_12229	NO_A/G	7	19	A/G	10	22	7.78E-01	Without correction
700	AM_11205	NO_G/T	7	19	G/T	10	22	7.78E-01	Without correction
701	AM_10899	A/G	7	19	NO_A/G	10	22	7.78E-01	Without correction
702	AM_10726	NO_C/C	7	19	C/C	10	22	7.78E-01	Without correction
703	AM_14556	NO_T/T	14	33	T/T	3	8	1.00E+00	Without correction
704	AM_14321	NO_A/A	14	33	A/A	3	8	1.00E+00	Without correction
705	AM_14287	NO_T/T	14	33	T/T	3	8	1.00E+00	Without correction
706	AM_12215	NO_C/G	14	33	C/G	3	8	1.00E+00	Without correction
707	AM_12215	C/C	14	33	NO_C/C	3	8	1.00E+00	Without correction
708	AM_11466	NO_A/G	14	33	A/G	3	8	1.00E+00	Without correction
709	AM_11466	G/G	14	33	NO_G/G	3	8	1.00E+00	Without correction
710	AM_11054	NO_A/A	14	33	A/A	3	8	1.00E+00	Without correction
711	AM_11490	C/C	3	8	NO_C/C	14	33	1.00E+00	Without correction
712	AM_14568	NO_C/C	3	8	C/C	14	33	1.00E+00	Without correction
713	AM_15390	NO_G/G	17	39	G/G	0	2	1.00E+00	Without correction
714	AM_15095	NO_C/C	17	39	C/C	0	2	1.00E+00	Without correction
715	AM_14592	NO_A/T	17	39	A/T	0	2	1.00E+00	Without correction
716	AM_14444	NO_A/A	17	39	A/A	0	2	1.00E+00	Without correction
717	AM_14348	NO_T/T	17	39	T/T	0	2	1.00E+00	Without correction
718	AM_13959	NO_T/T	17	39	T/T	0	2	1.00E+00	Without correction
719	AM_13871	NO_A/A	17	39	A/A	0	2	1.00E+00	Without correction
720	AM_13817	NO_C/C	17	39	C/C	0	2	1.00E+00	Without correction

Result no.	Probe Set ID	Category 1	ClassA (n), category 1	ClassB (n), category 1	Category 2	ClassA (n), category 2	ClassB (n), category 2	Fisher exact test p-value	Multiple-testing correction
721	AM_13377	NO_T/T	17	39	T/T	0	2	1.00E+00	Without correction
722	AM_13364	NO_C/C	17	39	C/C	0	2	1.00E+00	Without correction
723	AM_13070	NO_G/G	17	39	G/G	0	2	1.00E+00	Without correction
724	AM_12205	NO_A/A	17	39	A/A	0	2	1.00E+00	Without correction
725	AM_10985	NO_G/G	17	39	G/G	0	2	1.00E+00	Without correction
726	AM_10947	NO_C/C	17	39	C/C	0	2	1.00E+00	Without correction
727	AM_10611	NO_G/G	17	39	G/G	0	2	1.00E+00	Without correction
728	AM_10581	NO_G/G	17	39	G/G	0	2	1.00E+00	Without correction
729	AM_10152	NO_A/A	17	39	A/A	0	2	1.00E+00	Without correction
730	AM_15407	NO_A/G	16	38	A/G	1	3	1.00E+00	Without correction
731	AM_14993	NO_T/T	16	38	T/T	1	3	1.00E+00	Without correction
732	AM_14955	T/T	1	3	NO_T/T	16	38	1.00E+00	Without correction
733	AM_14704	NO_G/G	16	38	G/G	1	3	1.00E+00	Without correction
734	AM_13256	NO_T/T	16	38	T/T	1	3	1.00E+00	Without correction
735	AM_13175	NO_C/C	16	38	C/C	1	3	1.00E+00	Without correction
736	AM_13166	NO_A/A	16	38	A/A	1	3	1.00E+00	Without correction
737	AM_12778	T/T	1	3	NO_T/T	16	38	1.00E+00	Without correction
738	AM_11456	NO_A/A	16	38	A/A	1	3	1.00E+00	Without correction
739	AM_11407	NO_G/G	16	38	G/G	1	3	1.00E+00	Without correction
740	AM_11300	NO_T/T	16	38	T/T	1	3	1.00E+00	Without correction
741	AM_11269	NO_C/C	16	38	C/C	1	3	1.00E+00	Without correction
742	AM_10825	NO_A/A	16	38	A/A	1	3	1.00E+00	Without correction
743	AM_10816	NO_A/A	16	38	A/A	1	3	1.00E+00	Without correction
744	AM_10724	NO_A/A	16	38	A/A	1	3	1.00E+00	Without correction
745	AM_14553	NO_A/T	12	28	A/T	5	13	1.00E+00	Without correction
746	AM_13759	T/T	12	28	NO_T/T	5	13	1.00E+00	Without correction
747	AM_13603	NO_C/C	12	28	C/C	5	13	1.00E+00	Without correction
748	AM_13182	NO_A/G	12	28	A/G	5	13	1.00E+00	Without correction
749	AM_13182	G/G	12	28	NO_G/G	5	13	1.00E+00	Without correction
750	AM_12370	NO_C/T	12	28	C/T	5	13	1.00E+00	Without correction
751	AM_12247	NO_G/G	12	28	G/G	5	13	1.00E+00	Without correction
752	AM_11487	NO_C/T	12	28	C/T	5	13	1.00E+00	Without correction
753	AM_10816	NO_A/G	12	28	A/G	5	13	1.00E+00	Without correction
754	AM_10603	NO_A/A	12	28	A/A	5	13	1.00E+00	Without correction
755	AM_10031	NO_G/G	12	28	G/G	5	13	1.00E+00	Without correction
756	AM_10025	G/G	12	28	NO_G/G	5	13	1.00E+00	Without correction
757	AM_14556	NO_C/C	13	30	C/C	4	11	1.00E+00	Without correction
758	AM_14296	NO_T/T	13	30	T/T	4	11	1.00E+00	Without correction
759	AM_12304	NO_C/T	13	30	C/T	4	11	1.00E+00	Without correction
760	AM_12304	T/T	13	30	NO_T/T	4	11	1.00E+00	Without correction
761	AM_11912	NO_T/T	13	30	T/T	4	11	1.00E+00	Without correction
762	AM_11078	NO_C/C	4	11	C/C	13	30	1.00E+00	Without correction
763	AM_10025	NO_A/G	13	30	A/G	4	11	1.00E+00	Without correction
764	AM_13463	A/G	5	13	NO_A/G	12	28	1.00E+00	Without correction
765	AM_13365	G/G	5	13	NO_G/G	12	28	1.00E+00	Without correction
766	AM_12165	C/T	5	13	NO_C/T	12	28	1.00E+00	Without correction
767	AM_10675	C/C	5	13	NO_C/C	12	28	1.00E+00	Without correction
768	AM_15047	NO_A/A	12	30	A/A	5	11	1.00E+00	Without correction
769	AM_10959	NO_A/G	12	30	A/G	5	11	1.00E+00	Without correction
770	AM_10959	G/G	12	30	NO_G/G	5	11	1.00E+00	Without correction
771	AM_14934	NO_A/G	14	35	A/G	3	6	1.00E+00	Without correction
772	AM_14681	NO_A/A	15	34	A/A	2	7	1.00E+00	Without correction

Result no.	Probe Set ID	Category 1	ClassA (n), category 1	ClassB (n), category 1	Category 2	ClassA (n), category 2	ClassB (n), category 2	Fisher exact test p-value	Multiple-testing correction
773	AM_14568	C/T	2	7	NO_C/T	15	34	1.00E+00	Without correction
774	AM_13801	NO_A/G	15	34	A/G	2	7	1.00E+00	Without correction
775	AM_13801	A/A	15	34	NO_A/A	2	7	1.00E+00	Without correction
776	AM_13478	NO_C/T	14	35	C/T	3	6	1.00E+00	Without correction
777	AM_13192	NO_A/A	15	34	A/A	2	7	1.00E+00	Without correction
778	AM_12174	A/A	3	6	NO_A/A	14	35	1.00E+00	Without correction
779	AM_12147	NO_C/C	15	34	C/C	2	7	1.00E+00	Without correction
780	AM_11835	NO_A/G	14	35	A/G	3	6	1.00E+00	Without correction
781	AM_11280	NO_A/A	14	35	A/A	3	6	1.00E+00	Without correction
782	AM_10769	NO_A/C	15	34	A/C	2	7	1.00E+00	Without correction
783	AM_10769	C/C	15	34	NO_C/C	2	7	1.00E+00	Without correction
784	AM_10692	NO_T/T	14	35	T/T	3	6	1.00E+00	Without correction
785	AM_10206	NO_C/C	15	34	C/C	2	7	1.00E+00	Without correction
786	AM_15390	NO_G	11	25	G	6	16	1.00E+00	Without correction
787	AM_15050	NO_T/T	11	25	T/T	6	16	1.00E+00	Without correction
788	AM_13337	NO_G/G	11	25	G/G	6	16	1.00E+00	Without correction
789	AM_12535	NO_C/C	11	25	C/C	6	16	1.00E+00	Without correction
790	AM_12523	NO_A/A	11	25	A/A	6	16	1.00E+00	Without correction
791	AM_12261	NO_C/T	11	25	C/T	6	16	1.00E+00	Without correction
792	AM_11364	NO_G/G	11	25	G/G	6	16	1.00E+00	Without correction
793	AM_10816	G/G	11	25	NO_G/G	6	16	1.00E+00	Without correction
794	AM_10692	NO_C/C	11	25	C/C	6	16	1.00E+00	Without correction
795	AM_10048	NO_C/T	11	25	C/T	6	16	1.00E+00	Without correction
796	AM_11131	T/T	6	16	NO_T/T	11	25	1.00E+00	Without correction
797	AM_10748	A/A	6	16	NO_A/A	11	25	1.00E+00	Without correction
798	AM_11376	T/T	5	11	NO_T/T	12	30	1.00E+00	Without correction
799	AM_10503	C/C	5	11	NO_C/C	12	30	1.00E+00	Without correction
800	AM_14553	A/A	11	27	NO_A/A	6	14	1.00E+00	Without correction
801	AM_14216	NO_A/G	11	27	A/G	6	14	1.00E+00	Without correction
802	AM_10671	C/C	11	27	NO_C/C	6	14	1.00E+00	Without correction
803	AM_10666	NO_A/G	11	27	A/G	6	14	1.00E+00	Without correction
804	AM_14993	NO_G/T	11	26	G/T	6	15	1.00E+00	Without correction
805	AM_11912	NO_C/C	11	26	C/C	6	15	1.00E+00	Without correction
806	AM_11744	NO_A/T	11	26	A/T	6	15	1.00E+00	Without correction
807	AM_11426	C/C	11	26	NO_C/C	6	15	1.00E+00	Without correction
808	AM_12165	NO_C/C	7	16	C/C	10	25	1.00E+00	Without correction
809	AM_10977	C/C	7	16	NO_C/C	10	25	1.00E+00	Without correction
810	AM_10501	C/T	7	16	NO_C/T	10	25	1.00E+00	Without correction
811	AM_14704	NO_C/C	6	15	C/C	11	26	1.00E+00	Without correction
812	AM_14111	C/T	6	15	NO_C/T	11	26	1.00E+00	Without correction
813	AM_13140	C/C	6	15	NO_C/C	11	26	1.00E+00	Without correction
814	AM_11269	C/T	6	15	NO_C/T	11	26	1.00E+00	Without correction
815	AM_10985	NO_A/A	6	15	A/A	11	26	1.00E+00	Without correction
816	AM_13894	NO_T/T	15	37	T/T	2	4	1.00E+00	Without correction
817	AM_12726	NO_T/T	15	37	T/T	2	4	1.00E+00	Without correction
818	AM_12327	NO_C/C	15	37	C/C	2	4	1.00E+00	Without correction
819	AM_11310	NO_G/G	15	37	G/G	2	4	1.00E+00	Without correction
820	AM_10597	C/C	2	4	NO_C/C	15	37	1.00E+00	Without correction
821	AM_10595	NO_G/C	15	37	G/C	2	4	1.00E+00	Without correction
822	AM_13760	NO_G/G	8	19	G/G	9	22	1.00E+00	Without correction
823	AM_13163	NO_C/T	8	19	C/T	9	22	1.00E+00	Without correction
824	AM_12947	-/T	8	19	NO_-/T	9	22	1.00E+00	Without correction

Result no.	Probe Set ID	Category 1	ClassA (n), category 1	ClassB (n), category 1	Category 2	ClassA (n), category 2	ClassB (n), category 2	Fisher exact test p-value	Multiple-testing correction
825	AM_11147	NO_A/A	8	19	A/A	9	22	1.00E+00	Without correction
826	AM_10692	C/T	8	19	NO_C/T	9	22	1.00E+00	Without correction
827	AM_10540	A/T	8	19	NO_A/T	9	22	1.00E+00	Without correction
828	AM_13346	NO_C/C	9	22	C/C	8	19	1.00E+00	Without correction
829	AM_13070	NO_C/C	9	22	C/C	8	19	1.00E+00	Without correction
831	AM_12852	C/T	9	22	NO_C/T	8	19	1.00E+00	Without correction
832	AM_12656	NO_A/G	9	22	A/G	8	19	1.00E+00	Without correction
833	AM_10807	NO_C/T	9	22	C/T	8	19	1.00E+00	Without correction
834	AM_10333	C/G	9	22	NO_C/G	8	19	1.00E+00	Without correction
835	AM_15094	NO_A/A	16	37	A/A	1	4	1.00E+00	Without correction
836	AM_15085	NO_A/A	16	37	A/A	1	4	1.00E+00	Without correction
837	AM_14896	NO_G/G	16	37	G/G	1	4	1.00E+00	Without correction
838	AM_14661	NO_A/A	16	37	A/A	1	4	1.00E+00	Without correction
839	AM_12216	NO_A/A	16	37	A/A	1	4	1.00E+00	Without correction
840	AM_11487	NO_T/T	16	37	T/T	1	4	1.00E+00	Without correction
841	AM_11279	NO_A/A	16	37	A/A	1	4	1.00E+00	Without correction
842	AM_11168	NO_A/A	16	37	A/A	1	4	1.00E+00	Without correction
843	AM_11005	NO_A/A	16	37	A/A	1	4	1.00E+00	Without correction
844	AM_10711	NO_C/C	16	37	C/C	1	4	1.00E+00	Without correction
845	AM_10697	NO_T/T	16	37	T/T	1	4	1.00E+00	Without correction
846	AM_15202	NO_G/T	10	25	G/T	7	16	1.00E+00	Without correction
847	AM_14518	G/G	10	25	NO_G/G	7	16	1.00E+00	Without correction
848	AM_13507	NO_A/A	10	25	A/A	7	16	1.00E+00	Without correction
849	AM_12147	C/T	10	25	NO_C/T	7	16	1.00E+00	Without correction
850	AM_11219	NO_A/G	10	25	A/G	7	16	1.00E+00	Without correction
851	AM_10944	T/T	6	14	NO_T/T	11	27	1.00E+00	Without correction
852	AM_10825	G/G	6	14	NO_G/G	11	27	1.00E+00	Without correction
853	AM_14166	NO_G/G	13	31	G/G	4	10	1.00E+00	Without correction
854	AM_12852	NO_T/T	13	31	T/T	4	10	1.00E+00	Without correction
855	AM_12057	NO_C/T	13	31	C/T	4	10	1.00E+00	Without correction
856	AM_12057	C/C	13	31	NO_C/C	4	10	1.00E+00	Without correction
857	AM_12028	NO_C/T	13	31	C/T	4	10	1.00E+00	Without correction
858	AM_12028	C/C	13	31	NO_C/C	4	10	1.00E+00	Without correction
859	AM_10947	T/T	13	31	NO_T/T	4	10	1.00E+00	Without correction
860	AM_12852	NO_C/C	13	32	C/C	4	9	1.00E+00	Without correction
861	AM_11710	NO_C/C	13	32	C/C	4	9	1.00E+00	Without correction
862	AM_11499	NO_A/A	13	32	A/A	4	9	1.00E+00	Without correction
863	AM_11032	NO_A/G	13	32	A/G	4	9	1.00E+00	Without correction
864	AM_11032	A/A	13	32	NO_A/A	4	9	1.00E+00	Without correction
865	AM_10900	NO_A/A	13	32	A/A	4	9	1.00E+00	Without correction
866	AM_10010	NO_T/T	13	32	T/T	4	9	1.00E+00	Without correction
867	AM_14934	G/G	14	34	NO_G/G	3	7	1.00E+00	Without correction
868	AM_14609	NO_C/C	14	34	C/C	3	7	1.00E+00	Without correction
869	AM_13478	T/T	14	34	NO_T/T	3	7	1.00E+00	Without correction
870	AM_10748	NO_G/G	14	34	G/G	3	7	1.00E+00	Without correction
871	AM_10698	NO_C/T	14	34	C/T	3	7	1.00E+00	Without correction
872	AM_10595	NO_C/C	14	34	C/C	3	7	1.00E+00	Without correction
873	AM_14993	G/G	10	23	NO_G/G	7	18	1.00E+00	Without correction
874	AM_13760	NO_G/T	10	23	G/T	7	18	1.00E+00	Without correction
875	AM_13727	NO_C/T	10	23	C/T	7	18	1.00E+00	Without correction
876	AM_10183	C/T	10	23	NO_C/T	7	18	1.00E+00	Without correction
877	AM_10595	G/G	3	9	NO_G/G	14	32	1.00E+00	Without correction

Result no.	Probe Set ID	Category 1	ClassA (n), category 1	ClassB (n), category 1	Category 2	ClassA (n), category 2	ClassB (n), category 2	Fisher exact test p-value	Multiple-testing correction
878	AM_15085	NO_A/T	14	32	A/T	3	9	1.00E+00	Without correction
879	AM_14471	NO_A/G	14	32	A/G	3	9	1.00E+00	Without correction
880	AM_12857	NO_C/C	14	32	C/C	3	9	1.00E+00	Without correction
881	AM_12208	NO_G/G	14	32	G/G	3	9	1.00E+00	Without correction
882	AM_11948	NO_G/T	14	32	G/T	3	9	1.00E+00	Without correction
883	AM_11948	T/T	14	32	NO_T/T	3	9	1.00E+00	Without correction
884	AM_11938	NO_G/G	14	32	G/G	3	9	1.00E+00	Without correction
885	AM_11235	NO_A/G	14	32	A/G	3	9	1.00E+00	Without correction
886	AM_11235	G/G	14	32	NO_G/G	3	9	1.00E+00	Without correction
887	AM_10712	NO_C/T	14	32	C/T	3	9	1.00E+00	Without correction
888	AM_10712	T/T	14	32	NO_T/T	3	9	1.00E+00	Without correction
889	AM_10686	NO_A/G	14	32	A/G	3	9	1.00E+00	Without correction
890	AM_10686	A/A	14	32	NO_A/A	3	9	1.00E+00	Without correction
891	AM_13011	NO_G/G	12	29	G/G	5	12	1.00E+00	Without correction
892	AM_11243	NO_A/G	12	29	A/G	5	12	1.00E+00	Without correction
893	AM_11243	A/A	12	29	NO_A/A	5	12	1.00E+00	Without correction
894	AM_10711	T/T	12	29	NO_T/T	5	12	1.00E+00	Without correction
895	AM_10633	NO_G/G	12	29	G/G	5	12	1.00E+00	Without correction
896	AM_10047	C/C	12	29	NO_C/C	5	12	1.00E+00	Without correction
897	AM_12181	NO_A/A	15	35	A/A	2	6	1.00E+00	Without correction
898	AM_11474	NO_G/G	15	35	G/G	2	6	1.00E+00	Without correction
899	AM_10944	NO_A/A	15	35	A/A	2	6	1.00E+00	Without correction
900	AM_10473	NO_C/C	15	35	C/C	2	6	1.00E+00	Without correction
901	AM_12225	NO_G/G	3	7	G/G	14	34	1.00E+00	Without correction
902	AM_12225	A/G	3	7	NO_A/G	14	34	1.00E+00	Without correction
903	AM_13534	NO_C/C	15	36	C/C	2	5	1.00E+00	Without correction
904	AM_13326	NO_C/C	15	36	C/C	2	5	1.00E+00	Without correction
905	AM_12230	NO_A/G	15	36	A/G	2	5	1.00E+00	Without correction
906	AM_12230	G/G	15	36	NO_G/G	2	5	1.00E+00	Without correction
907	AM_12169	G/G	2	5	NO_G/G	15	36	1.00E+00	Without correction
908	AM_11730	NO_A/A	15	36	A/A	2	5	1.00E+00	Without correction
909	AM_11299	NO_A/A	15	36	A/A	2	5	1.00E+00	Without correction
910	AM_10501	NO_T/T	15	36	T/T	2	5	1.00E+00	Without correction
911	AM_12145	NO_G/G	4	9	G/G	13	32	1.00E+00	Without correction
912	AM_12145	A/G	4	9	NO_A/G	13	32	1.00E+00	Without correction
913	AM_14704	C/G	5	12	NO_C/G	12	29	1.00E+00	Without correction
914	AM_13507	A/G	9	23	NO_A/G	8	18	1.00E+00	Without correction
915	AM_11219	G/G	9	23	NO_G/G	8	18	1.00E+00	Without correction
916	AM_10903	NO_A/G	9	23	A/G	8	18	1.00E+00	Without correction
917	AM_10748	NO_A/G	9	23	A/G	8	18	1.00E+00	Without correction
918	AM_11474	C/C	8	20	NO_C/C	9	21	1.00E+00	Without correction
919	AM_10944	NO_A/T	8	20	A/T	9	21	1.00E+00	Without correction
920	AM_10595	NO_C/G	8	20	C/G	9	21	1.00E+00	Without correction
921	AM_15307	A/G	8	20	NO_A/G	9	21	1.00E+00	Without correction
922	AM_13207	NO_C/T	8	20	C/T	9	21	1.00E+00	Without correction
923	AM_15095	NO_A/A	8	18	A/A	9	23	1.00E+00	Without correction
924	AM_14336	A/G	8	18	NO_A/G	9	23	1.00E+00	Without correction
925	AM_14132	NO_T/T	8	18	T/T	9	23	1.00E+00	Without correction
926	AM_12385	C/C	8	18	NO_C/C	9	23	1.00E+00	Without correction
927	AM_12208	NO_A/G	8	18	A/G	9	23	1.00E+00	Without correction
928	AM_11147	A/G	8	18	NO_A/G	9	23	1.00E+00	Without correction
929	AM_11054	NO_A/G	8	18	A/G	9	23	1.00E+00	Without correction

Result no.	Probe Set ID	Category 1	ClassA (n), category 1	ClassB (n), category 1	Category 2	ClassA (n), category 2	ClassB (n), category 2	Fisher exact test p-value	Multiple-testing correction
930	AM_10913	NO_A/G	8	18	A/G	9	23	1.00E+00	Without correction
931	AM_10900	G/G	8	18	NO_G/G	9	23	1.00E+00	Without correction
932	AM_11269	NO_T/T	7	18	T/T	10	23	1.00E+00	Without correction
933	AM_15094	A/G	7	18	NO_A/G	10	23	1.00E+00	Without correction
934	AM_14296	C/T	7	18	NO_C/T	10	23	1.00E+00	Without correction
935	AM_15390	A	9	20	NO_A	8	21	1.00E+00	Without correction
936	AM_13603	C/T	8	21	NO_C/T	9	20	1.00E+00	Without correction
937	AM_13509	T/T	8	21	NO_T/T	9	20	1.00E+00	Without correction
938	AM_13070	C/G	9	20	NO_C/G	8	21	1.00E+00	Without correction
939	AM_12726	C/T	9	20	NO_C/T	8	21	1.00E+00	Without correction
940	AM_12322	C/T	9	20	NO_C/T	8	21	1.00E+00	Without correction
941	AM_11034	A/G	9	20	NO_A/G	8	21	1.00E+00	Without correction
942	AM_11022	A/G	8	21	NO_A/G	9	20	1.00E+00	Without correction
943	AM_10903	A/A	9	20	NO_A/A	8	21	1.00E+00	Without correction
944	AM_10742	T/T	2	6	NO_T/T	15	35	1.00E+00	Without correction
945	AM_12170	NO_C/C	9	21	C/C	8	20	1.00E+00	Without correction
946	AM_11490	NO_C/T	9	21	C/T	8	20	1.00E+00	Without correction
947	AM_11490	NO_T/T	11	28	T/T	6	13	1.00E+00	Without correction
948	AM_11280	NO_G/G	12	27	G/G	5	14	1.00E+00	Without correction
949	AM_11280	A/G	9	21	NO_A/G	8	20	1.00E+00	Without correction
950	AM_11426	NO_C/T	11	28	C/T	6	13	1.00E+00	Without correction
951	AM_10920	C/T	9	21	NO_C/T	8	20	1.00E+00	Without correction
952	AM_10900	NO_A/G	12	27	A/G	5	14	1.00E+00	Without correction
953	AM_10501	NO_C/C	9	21	C/C	8	20	1.00E+00	Without correction
954	AM_13635	NO_G/G	12	27	G/G	5	14	1.00E+00	Without correction
955	AM_13509	NO_C/T	9	21	C/T	8	20	1.00E+00	Without correction
956	AM_13377	C/C	11	28	NO_C/C	6	13	1.00E+00	Without correction
957	AM_13192	NO_A/C	9	21	A/C	8	20	1.00E+00	Without correction
958	AM_12590	C/C	9	21	NO_C/C	8	20	1.00E+00	Without correction
959	AM_14132	C/T	5	14	NO_C/T	12	27	1.00E+00	Without correction
960	AM_13896	G/G	6	13	NO_G/G	11	28	1.00E+00	Without correction
961	AM_13558	NO_A/A	6	13	A/A	11	28	1.00E+00	Without correction
962	AM_13558	A/G	6	13	NO_A/G	11	28	1.00E+00	Without correction
963	AM_13307	A/A	6	13	NO_A/A	11	28	1.00E+00	Without correction
964	AM_13298	A/A	6	13	NO_A/A	11	28	1.00E+00	Without correction
965	AM_13163	C/C	5	14	NO_C/C	12	27	1.00E+00	Without correction
966	AM_12216	NO_A/G	6	13	A/G	11	28	1.00E+00	Without correction
967	AM_10985	A/G	6	13	NO_A/G	11	28	1.00E+00	Without correction
969	AM_11669	NO_C/C	16	39	C/C	1	2	1.00E+00	Without correction
970	AM_11219	NO_A/A	16	39	A/A	1	2	1.00E+00	Without correction
971	AM_11203	NO_A/A	16	39	A/A	1	2	1.00E+00	Without correction
972	AM_10389	NO_A/A	16	39	A/A	1	2	1.00E+00	Without correction
973	AM_10025	NO_A/A	16	39	A/A	1	2	1.00E+00	Without correction
974	AM_15407	NO_A/A	16	39	A/A	1	2	1.00E+00	Without correction
975	AM_15390	NO_A/G	16	39	A/G	1	2	1.00E+00	Without correction
976	AM_15380	NO_C/C	16	39	C/C	1	2	1.00E+00	Without correction
977	AM_15380	NO_C/T	16	39	C/T	1	2	1.00E+00	Without correction
978	AM_14592	NO_A/G	16	39	A/G	1	2	1.00E+00	Without correction
979	AM_13536	NO_A/A	16	39	A/A	1	2	1.00E+00	Without correction
980	AM_13507	NO_G/G	16	39	G/G	1	2	1.00E+00	Without correction
981	AM_13153	NO_G/G	16	39	G/G	1	2	1.00E+00	Without correction
982	AM_13323	NO_T/T	10	24	T/T	7	17	1.00E+00	Without correction

Result no.	Probe Set ID	Category 1	ClassA (n), category 1	ClassB (n), category 1	Category 2	ClassA (n), category 2	ClassB (n), category 2	Fisher exact test p-value	Multiple-testing correction
983	**AM_13019**	**NO_G/G**	**10**	**24**	**G/G**	**7**	**17**	**1.00E+00**	**Without correction**
984	**AM_11500**	**NO_G/G**	**10**	**24**	**G/G**	**7**	**17**	**1.00E+00**	**Without correction**
985	**AM_10899**	**NO_G/G**	**10**	**24**	**G/G**	**7**	**17**	**1.00E+00**	**Without correction**
986	**AM_12137**	**C/T**	**7**	**17**	**NO_C/T**	**10**	**24**	**1.00E+00**	**Without correction**
987	**AM_10825**	**NO_A/G**	**7**	**17**	**A/G**	**10**	**24**	**1.00E+00**	**Without correction**
988	**AM_10730**	**A/A**	**7**	**17**	**NO_A/A**	**10**	**24**	**1.00E+00**	**Without correction**
989	**AM_10020**	**NO_T/T**	**17**	**40**	**T/T**	**0**	**1**	**1.00E+00**	**Without correction**
990	**AM_10020**	**NO_T/T**	**17**	**40**	**T/T**	**0**	**1**	**1.00E+00**	**Without correction**
991	**AM_10135**	**NO_A/A**	**17**	**40**	**A/A**	**0**	**1**	**1.00E+00**	**Without correction**
992	**AM_10053**	**NO_T/T**	**17**	**40**	**T/T**	**0**	**1**	**1.00E+00**	**Without correction**
993	**AM_10490**	**NO_G/G**	**17**	**40**	**G/G**	**0**	**1**	**1.00E+00**	**Without correction**
994	**AM_10691**	**NO_C/C**	**17**	**40**	**C/C**	**0**	**1**	**1.00E+00**	**Without correction**
995	**AM_10673**	**NO_C/T**	**17**	**40**	**C/T**	**0**	**1**	**1.00E+00**	**Without correction**
996	**AM_10659**	**NO_A/A**	**17**	**40**	**A/A**	**0**	**1**	**1.00E+00**	**Without correction**
997	**AM_11024**	**NO_A/A**	**17**	**40**	**A/A**	**0**	**1**	**1.00E+00**	**Without correction**
998	AM_11135	NO_C/T	17	40	C/T	0	1	1.00E+00	Without correction

Code 1. Pipeline analysis configuration parameters


```json
{
  "input": "./opbg.csv",
  "output_dir": "output_tree_model_23Sept2026",
  "target": "class",
  "positive_class": "RE",
  "test_size": 0.2,
  "seed": 42,
  "folds": 5,
  "repeats": 10,
  "bootstrap": 200,
  "max_depth": 4,
  "min_samples_split": 10,
  "min_samples_leaf": 5
}
```


Code 1. Report the parameters used to configure the analysis pipeline in Json format.

Code 2. Analysis Pipeline code Python

```
from __future__ import annotations

import argparse
import json
import os
import re
from pathlib import Path
from typing import Iterable

import matplotlib
matplotlib.use("Agg")
import matplotlib.pyplot as plt
import numpy as np
import pandas as pd
```

```
from imblearn.over_sampling import RandomOverSampler
from imblearn.pipeline import Pipeline as ImbPipeline
from sklearn.base import clone
from sklearn.compose import ColumnTransformer
from sklearn.feature_selection import SelectFromModel
from sklearn.impute import SimpleImputer
from sklearn.metrics import (
    ConfusionMatrixDisplay,
    accuracy_score,
    classification_report,
    confusion_matrix,
    f1_score,
    precision_score,
    recall_score,
    roc_auc_score,
    roc_curve,
)
from sklearn.model_selection import RepeatedStratifiedKFold, train_test_split
from sklearn.pipeline import Pipeline as SkPipeline
```

```
from sklearn.preprocessing import OneHotEncoder
from sklearn.svm import LinearSVC
from sklearn.tree import DecisionTreeClassifier, export_graphviz, export_text, plot_tree


METRICS = ("accuracy", "precision", "recall", "f1", "roc_auc")
REVIEWER_FEATURES = (
    "CHST3", "CHST11", "SULT1A2/A3", "NR1I3", "food allergy"
)


def parse_args() -> argparse.Namespace:
    parser = argparse.ArgumentParser(description=__doc__)
    parser.add_argument("--input", required=True, help="Input CSV file")
    parser.add_argument("--output-dir", default="output_tree_model_repeated_cv")
    parser.add_argument("--target", default="class")
    parser.add_argument("--positive-class", default="RE")
    parser.add_argument("--test-size", type=float, default=0.20)
    parser.add_argument("--seed", type=int, default=42)
```

```
    parser.add_argument("--folds", type=int, default=5)
    parser.add_argument("--repeats", type=int, default=10)
    parser.add_argument("--bootstrap", type=int, default=200)
    parser.add_argument("--max-depth", type=int, default=4)
    parser.add_argument("--min-samples-split", type=int, default=10)
    parser.add_argument("--min-samples-leaf", type=int, default=5)
    return parser.parse_args()


def load_csv_robustly(path: str) -> pd.DataFrame:
    last_error = None
    for encoding in ("utf-8", "latin1", "cp1252", "iso-8859-1"):
        for separator in (",", ";", "\t"):
            try:
                frame = pd.read_csv(path, encoding=encoding, sep=separator)
                if frame.shape[1] > 1:
                    print(f"CSV loaded with encoding={encoding}, sep={separator!r}")
                    return frame
            except Exception as exc:
```

```
            last_error = exc
    raise RuntimeError(f"Unable to load CSV. Last error: {last_error}")


def clean_feature_name(name: str) -> str:
    """Remove ColumnTransformer prefixes while retaining the original column name."""
    return re.sub(r"^(num|cat)__", "", str(name))


def normalized_name(name: str) -> str:
    return re.sub(r"[^a-z0-9]", "", clean_feature_name(name).lower())


def reviewer_group(feature_name: str) -> str | None:
    clean_name = clean_feature_name(feature_name).strip()
    value = normalized_name(feature_name)
    aliases = {
        "CHST3": ("chst3",),
        "CHST11": ("chst11",),
```

```
        "SULT1A2/A3": ("sult1a2a3", "sult1a2_a3"),
        "NR1I3": ("nr1i3",),
        "food allergy": ("foodallergy", "allergia alimentare", "allergiaalimentare"),
    }
    for label, tokens in aliases.items():
        if any(normalized_name(token) in value for token in tokens):
            return label
    return None


def build_pipeline(X: pd.DataFrame, args: argparse.Namespace) -> ImbPipeline:
    probe_columns = [column for column in X.columns if str(column).upper().startswith("AM_")]
    categorical = list(dict.fromkeys(
        X.select_dtypes(include=["object", "category", "bool"]).columns.tolist()
        + probe_columns
    ))
    numeric = [column for column in X.columns if column not in categorical]

    numeric_transformer = SkPipeline([
```

```
    ("imputer", SimpleImputer(strategy="most_frequent")),
])
categorical_transformer = SkPipeline([
    ("imputer", SimpleImputer(strategy="most_frequent")),
    ("encoder", OneHotEncoder(handle_unknown="ignore", sparse_output=False)),
])
preprocessor = ColumnTransformer([
    ("num", numeric_transformer, numeric),
    ("cat", categorical_transformer, categorical),
], remainder="drop", verbose_feature_names_out=True)

return ImbPipeline([
    ("preprocess", preprocessor),
    ("feature_selection", SelectFromModel(
        LinearSVC(
            C=10,
            penalty="l2",
            dual=False,
            max_iter=5000,
```

```
            random_state=args.seed,
            class_weight="balanced",
        ),
        threshold="mean",
    )),
    ("oversample", RandomOverSampler(random_state=args.seed)),
    ("classifier", DecisionTreeClassifier(
        random_state=args.seed,
        max_depth=args.max_depth,
        min_samples_split=args.min_samples_split,
        min_samples_leaf=args.min_samples_leaf,
    )),
])


def positive_scores(model: ImbPipeline, X: pd.DataFrame, positive_class: str) -> np.ndarray:
    classes = list(model.classes_)
    if positive_class not in classes:
        raise ValueError(f"Positive class {positive_class!r} not found in {classes}")
```

```python
    if hasattr(model, "predict_proba"):
        return model.predict_proba(X)[:, classes.index(positive_class)]
    if hasattr(model, "decision_function"):
        raw = np.asarray(model.decision_function(X))
        if raw.ndim == 1:
            return raw if classes[1] == positive_class else -raw
        return raw[:, classes.index(positive_class)]
    raise AttributeError("The fitted model provides neither probabilities nor decision scores")


def calculate_metrics(y_true: pd.Series, y_pred: np.ndarray, y_score: np.ndarray,
                      positive_class: str) -> dict[str, float]:
    binary = (np.asarray(y_true) == positive_class).astype(int)
    return {
        "accuracy": accuracy_score(y_true, y_pred),
        "precision": precision_score(y_true, y_pred, pos_label=positive_class, zero_division=0),
        "recall": recall_score(y_true, y_pred, pos_label=positive_class, zero_division=0),
        "f1": f1_score(y_true, y_pred, pos_label=positive_class, zero_division=0),
        "roc_auc": roc_auc_score(binary, y_score) if np.unique(binary).size == 2 else np.nan,
```

```
    }


def fitted_feature_information(model: ImbPipeline) -> tuple[list[str], list[str]]:
    preprocessed = model.named_steps["preprocess"].get_feature_names_out()
    selected_mask = model.named_steps["feature_selection"].get_support()
    selected = [clean_feature_name(x) for x in preprocessed[selected_mask]]
    tree = model.named_steps["classifier"].tree_
    used_indices = sorted(set(int(i) for i in tree.feature if i >= 0))
    used = [selected[i] for i in used_indices]
    return selected, used


def repeated_cross_validation(pipeline: ImbPipeline, X: pd.DataFrame, y: pd.Series,
                              args: argparse.Namespace) -> tuple[pd.DataFrame, pd.DataFrame]:
    splitter = RepeatedStratifiedKFold(
        n_splits=args.folds, n_repeats=args.repeats, random_state=args.seed
    )
    metric_rows, feature_rows = [], []
```

```
for split_number, (fit_idx, validation_idx) in enumerate(splitter.split(X, y), start=1):
    repetition = (split_number - 1) // args.folds + 1
    fold = (split_number - 1) % args.folds + 1
    model = clone(pipeline)
    X_fit, X_validation = X.iloc[fit_idx], X.iloc[validation_idx]
    y_fit, y_validation = y.iloc[fit_idx], y.iloc[validation_idx]
    model.fit(X_fit, y_fit)
    prediction = model.predict(X_validation)
    score = positive_scores(model, X_validation, args.positive_class)
    row = {
        "split": split_number,
        "repetition": repetition,
        "fold": fold,
        "n_fit": len(fit_idx),
        "n_validation": len(validation_idx),
        **calculate_metrics(y_validation, prediction, score, args.positive_class),
    }
    metric_rows.append(row)
```

```
        selected, used = fitted_feature_information(model)
        selected_set, used_set = set(selected), set(used)
        for feature in sorted(selected_set | used_set):
            feature_rows.append({
                "split": split_number,
                "repetition": repetition,
                "fold": fold,
                "feature": feature,
                "reviewer_feature_group": reviewer_group(feature),
                "selected": int(feature in selected_set),
                "used_by_tree": int(feature in used_set),
            })
    return pd.DataFrame(metric_rows), pd.DataFrame(feature_rows)


def metric_summary(values: pd.DataFrame, validation: str) -> pd.DataFrame:
    rows = []
    for metric in METRICS:
        data = values[metric].dropna().astype(float)
```

```
    n = len(data)
    mean, sd = data.mean(), data.std(ddof=1)
    sem = sd / np.sqrt(n) if n > 1 else np.nan
    from scipy.stats import t
    critical = t.ppf(0.975, n - 1) if n > 1 else np.nan
    rows.append({
        "validation": validation,
        "metric": metric,
        "n_estimates": n,
        "mean": mean,
        "sd": sd,
        "ci95_mean_lower": mean - critical * sem if n > 1 else np.nan,
        "ci95_mean_upper": mean + critical * sem if n > 1 else np.nan,
        "distribution_p2.5": data.quantile(0.025),
        "median": data.median(),
        "distribution_p97.5": data.quantile(0.975),
    })
return pd.DataFrame(rows)
```

```
def complete_feature_stability(feature_rows: pd.DataFrame, total_splits: int,
                               all_features: Iterable[str]) -> pd.DataFrame:
    aggregated = feature_rows.groupby("feature", as_index=False).agg(
        selected_count=("selected", "sum"),
        tree_use_count=("used_by_tree", "sum"),
    ) if not feature_rows.empty else pd.DataFrame(columns=["feature", "selected_count", "tree_use_count"])
    universe = pd.DataFrame({"feature": [clean_feature_name(x) for x in all_features]})
    result = universe.merge(aggregated, on="feature", how="left").fillna(0)
    result["selected_count"] = result["selected_count"].astype(int)
    result["tree_use_count"] = result["tree_use_count"].astype(int)
    result["selected_frequency"] = result["selected_count"] / total_splits
    result["tree_use_frequency"] = result["tree_use_count"] / total_splits
    result["reviewer_feature_group"] = result["feature"].map(reviewer_group)
    return result.sort_values(["tree_use_frequency", "selected_frequency"], ascending=False)


def reviewer_stability_table(stability: pd.DataFrame, total_splits: int) -> pd.DataFrame:
    rows = []
```

```
    for group in REVIEWER_FEATURES:
        subset = stability[stability["reviewer_feature_group"] == group]
        rows.append({
            "feature_or_group": group,
            "matching_columns": "; ".join(subset["feature"].astype(str)),
            "n_matching_columns": len(subset),
            "maximum_column_selection_frequency": subset["selected_frequency"].max() if len(subset) else 0.0,
            "maximum_column_tree_use_frequency": subset["tree_use_frequency"].max() if len(subset) else 0.0,
            "total_resampling_fits": total_splits,
        })
    return pd.DataFrame(rows)


def aggregate_group_stability(feature_rows: pd.DataFrame, total_splits: int,
                              available_features: Iterable[str]) -> pd.DataFrame:
    available = pd.DataFrame({"feature": [clean_feature_name(x) for x in available_features]})
    available["reviewer_feature_group"] = available["feature"].map(reviewer_group)
    rows = []
    for group in REVIEWER_FEATURES:
```

```
    part = feature_rows[feature_rows["reviewer_feature_group"] == group]
    available_group = available[available["reviewer_feature_group"] == group]
    n_available = available_group["feature"].nunique()
    selected_splits = part.loc[part["selected"] == 1, "split"].nunique()
    used_splits = part.loc[part["used_by_tree"] == 1, "split"].nunique()
    rows.append({
        "feature_or_group": group,
        "n_input_features": n_available,
        "input_features": "; ".join(sorted(available_group["feature"].unique())),
        "selected_in_fits": selected_splits,
        "selected_frequency": selected_splits / total_splits if n_available else np.nan,
        "used_by_tree_in_fits": used_splits,
        "tree_use_frequency": used_splits / total_splits if n_available else np.nan,
        "total_resampling_fits": total_splits,
        "status": "evaluated" if n_available else "not present in input matrix",
    })
return pd.DataFrame(rows)
```

```
def bootstrap_oob(pipeline: ImbPipeline, X: pd.DataFrame, y: pd.Series,
                  args: argparse.Namespace) -> pd.DataFrame:
    rng = np.random.RandomState(args.seed)
    X, y = X.reset_index(drop=True), y.reset_index(drop=True)
    rows = []
    for iteration in range(1, args.bootstrap + 1):
        fit_idx = rng.choice(len(X), size=len(X), replace=True)
        oob_idx = np.setdiff1d(np.arange(len(X)), np.unique(fit_idx))
        if len(oob_idx) == 0 or y.iloc[oob_idx].nunique() < 2 or y.iloc[fit_idx].nunique() < 2:
            continue
        model = clone(pipeline)
        try:
            model.fit(X.iloc[fit_idx], y.iloc[fit_idx])
            prediction = model.predict(X.iloc[oob_idx])
            score = positive_scores(model, X.iloc[oob_idx], args.positive_class)
            rows.append({
                "bootstrap_iteration": iteration,
                "n_bootstrap_fit": len(fit_idx),
                "n_oob": len(oob_idx),
```

```
                **calculate_metrics(y.iloc[oob_idx], prediction, score, args.positive_class),
            })
        except Exception as exc:
            print(f"Bootstrap iteration {iteration} skipped: {exc}")
    return pd.DataFrame(rows)


def save_latex(frame: pd.DataFrame, path: Path, caption: str, label: str,
               float_format: str = "%.4f") -> None:
    def escape(value: object) -> str:
        text = str(value)
        for old, new in (("\\", r"\textbackslash{}"), ("&", r"\&"),
                         ("%", r"\%"), ("$", r"\$"), ("#", r"\#"),
                         ("_", r"\_"), ("{", r"\{"), ("}", r"\}")):
            text = text.replace(old, new)
        return text

    def render(value: object) -> str:
        if pd.isna(value):
```

```
        return "--"
    if isinstance(value, (float, np.floating)):
        return float_format % float(value)
    return escape(value)

columns = list(frame.columns)
lines = [
    r"\begin{table}[htbp]", r"\centering", f"\\caption{{{escape(caption)}}}",
    f"\\label{{{label}}}", r"\small", r"\begin{tabular}{" + "l" + "c" * (len(columns) - 1) + "}",
    r"\toprule", " & ".join(escape(c) for c in columns) + r" \\", r"\midrule",
]
lines.extend(" & ".join(render(v) for v in row) + r" \\" for row in frame.itertuples(index=False, name=None))
lines.extend([r"\bottomrule", r"\end{tabular}", r"\end{table}"])
path.write_text("\n".join(lines) + "\n", encoding="utf-8")


def plot_metric_distributions(cv_results: pd.DataFrame, output: Path) -> None:
    fig, ax = plt.subplots(figsize=(9, 5.4))
    data = [cv_results[m].dropna().values for m in METRICS]
```

```
    parts = ax.violinplot(data, showmeans=False, showmedians=False, showextrema=False)
    for body in parts["bodies"]:
        body.set_facecolor("#4C9BC3")
        body.set_edgecolor("#17324D")
        body.set_alpha(0.65)
    ax.boxplot(data, widths=0.16, showfliers=False, patch_artist=True,
               boxprops={"facecolor": "white", "edgecolor": "#17324D"},
               medianprops={"color": "#D07628", "linewidth": 2})
    ax.set_xticks(range(1, len(METRICS) + 1), [m.replace("_", " ").upper() for m in METRICS])
    ax.set_ylabel("Validation score")
    ax.set_ylim(0, 1.03)
    ax.grid(axis="y", alpha=0.25)
    fig.tight_layout()
    fig.savefig(output / "repeated_cv_metric_distributions.png", dpi=300, bbox_inches="tight")
    fig.savefig(output / "repeated_cv_metric_distributions.pdf", bbox_inches="tight")
    plt.close(fig)


def plot_feature_stability(group_stability: pd.DataFrame, output: Path) -> None:
```

```python
positions = np.arange(len(group_stability))
width = 0.36
fig, ax = plt.subplots(figsize=(9, 5.5))
ax.barh(positions - width / 2, group_stability["selected_frequency"] * 100,
        height=width, label="Selected", color="#65A66C")
ax.barh(positions + width / 2, group_stability["tree_use_frequency"] * 100,
        height=width, label="Used by decision tree", color="#8067B3")
ax.set_yticks(positions, group_stability["feature_or_group"])
ax.invert_yaxis()
ax.set_xlim(0, 100)
ax.set_xlabel("Frequency across resampling fits (%)")
ax.legend(frameon=False, loc="lower right")
ax.grid(axis="x", alpha=0.25)
fig.tight_layout()
fig.savefig(output / "reviewer_feature_stability.png", dpi=300, bbox_inches="tight")
fig.savefig(output / "reviewer_feature_stability.pdf", bbox_inches="tight")
plt.close(fig)
```

```
def plot_test_outputs(y_test: pd.Series, prediction: np.ndarray, score: np.ndarray,
                      labels: list[str], positive_class: str, output: Path) -> float:
    matrix = confusion_matrix(y_test, prediction, labels=labels)
    fig, ax = plt.subplots(figsize=(5.8, 5.3))
    ConfusionMatrixDisplay(matrix, display_labels=labels).plot(ax=ax, cmap="Blues", values_format="d")
    fig.tight_layout()
    fig.savefig(output / "confusion_matrix_test.png", dpi=300, bbox_inches="tight")
    fig.savefig(output / "confusion_matrix_test.pdf", bbox_inches="tight")
    plt.close(fig)

    binary = (np.asarray(y_test) == positive_class).astype(int)
    auc_value = roc_auc_score(binary, score)
    fpr, tpr, _ = roc_curve(binary, score)
    fig, ax = plt.subplots(figsize=(5.8, 5.3))
    ax.plot(fpr, tpr, lw=2.2, color="#4C9BC3", label=f"AUC = {auc_value:.3f}")
    ax.plot([0, 1], [0, 1], "--", color="#647B8C")
    ax.set(xlabel="False-positive rate", ylabel="True-positive rate", xlim=(0, 1), ylim=(0, 1.02))
    ax.legend(loc="lower right", frameon=False)
    fig.tight_layout()
```

```
    fig.savefig(output / "roc_curve_test.png", dpi=300, bbox_inches="tight")
    fig.savefig(output / "roc_curve_test.pdf", bbox_inches="tight")
    plt.close(fig)
    return auc_value


def export_final_tree(model: ImbPipeline, output: Path) -> None:
    selected, _ = fitted_feature_information(model)
    tree = model.named_steps["classifier"]
    (output / "selected_features_final_model.txt").write_text("\n".join(selected) + "\n", encoding="utf-8")
    rules = export_text(tree, feature_names=selected)
    (output / "decision_tree_rules.txt").write_text(rules, encoding="utf-8")

    tree_structure = tree.tree_
    node_rows = []
    for node_id in range(tree_structure.node_count):
        left = int(tree_structure.children_left[node_id])
        right = int(tree_structure.children_right[node_id])
        is_leaf = left == right
```

```
feature_index = int(tree_structure.feature[node_id])
counts = np.asarray(tree_structure.value[node_id]).reshape(-1)
predicted_index = int(np.argmax(counts))
row = {
    "node_id": node_id,
    "is_leaf": is_leaf,
    "left_child": "" if is_leaf else left,
    "right_child": "" if is_leaf else right,
    "split_feature": "" if is_leaf else selected[feature_index],
    "threshold": np.nan if is_leaf else float(tree_structure.threshold[node_id]),
    "left_rule": "" if is_leaf else f"{selected[feature_index]} <= {tree_structure.threshold[node_id]:.6g}",
    "right_rule": "" if is_leaf else f"{selected[feature_index]} > {tree_structure.threshold[node_id]:.6g}",
    "samples": int(tree_structure.n_node_samples[node_id]),
    "weighted_samples": float(tree_structure.weighted_n_node_samples[node_id]),
    "predicted_class": str(tree.classes_[predicted_index]),
}
for class_index, class_name in enumerate(tree.classes_):
    row[f"class_count_{class_name}"] = float(counts[class_index])
node_rows.append(row)
```

```
pd.DataFrame(node_rows).to_csv(output / "decision_tree_nodes.csv", index=False)

figure_width = max(12.0, 3.5 * (2 ** min(tree.get_depth(), 4)))
fig, ax = plt.subplots(figsize=(figure_width, max(7.0, 2.4 * (tree.get_depth() + 1))))
plot_tree(
    tree,
    feature_names=selected,
    class_names=[str(c) for c in tree.classes_],
    filled=False,
    rounded=False,
    impurity=True,
    proportion=False,
    precision=4,
    ax=ax,
)
fig.tight_layout()
fig.savefig(output / "Figure1_decision_tree.svg", bbox_inches="tight")
fig.savefig(output / "Figure1_decision_tree.pdf", bbox_inches="tight")
fig.savefig(output / "Figure1_decision_tree.png", dpi=300, bbox_inches="tight")
```

```
    plt.close(fig)

    dot_path = output / "decision_tree.dot"
    export_graphviz(tree, out_file=str(dot_path), feature_names=selected,
                    class_names=[str(c) for c in tree.classes_], filled=True,
                    rounded=True, special_characters=True)
    try:
        import graphviz
        source = graphviz.Source(dot_path.read_text(encoding="utf-8"))
        source.render(filename=str(output / "decision_tree"), format="png", cleanup=True)
        source.render(filename=str(output / "decision_tree"), format="pdf", cleanup=True)
    except Exception as exc:
        print(f"Tree DOT saved; automatic PNG/PDF export unavailable: {exc}")


def main() -> None:
    args = parse_args()
    output = Path(args.output_dir)
    output.mkdir(parents=True, exist_ok=True)
```

```
(output / "analysis_configuration.json").write_text(
    json.dumps(vars(args), indent=2), encoding="utf-8"
)

df = load_csv_robustly(args.input)
df.columns = df.columns.str.strip()
if args.target not in df:
    raise KeyError(f"Target {args.target!r} not found. Columns: {df.columns.tolist()}")
df = df.loc[df[args.target].notna()].copy()
X, y = df.drop(columns=[args.target]), df[args.target].astype(str)
if args.positive_class not in set(y):
    raise ValueError(f"Positive class {args.positive_class!r} not in {sorted(set(y))}")

X_train, X_test, y_train, y_test = train_test_split(
    X, y, test_size=args.test_size, random_state=args.seed, stratify=y
)
split_summary = pd.DataFrame([
    {"partition": "full", "n": len(y), **y.value_counts().to_dict()},
    {"partition": "development", "n": len(y_train), **y_train.value_counts().to_dict()},
```

```
    {"partition": "independent_test", "n": len(y_test), **y_test.value_counts().to_dict()},
]).fillna(0)
split_summary.to_csv(output / "dataset_partitions.csv", index=False)
print("\nDataset partitions:\n", split_summary.to_string(index=False))

pipeline = build_pipeline(X_train, args)
cv_results, fold_features = repeated_cross_validation(pipeline, X_train, y_train, args)
cv_results.to_csv(output / "repeated_cv_fold_metrics.csv", index=False)
fold_features.to_csv(output / "repeated_cv_fold_features_long.csv", index=False)
cv_summary = metric_summary(cv_results, "Repeated stratified CV")
cv_summary.to_csv(output / "repeated_cv_summary.csv", index=False)
save_latex(
    cv_summary[["metric", "mean", "sd", "ci95_mean_lower", "ci95_mean_upper", "n_estimates"]],
    output / "table_repeated_cv_performance.tex",
    "Performance across repeated stratified cross-validation folds on the development set.",
    "tab:repeated_cv_performance",
)

# Obtain the full preprocessed feature universe without using test outcomes.
```

```
preprocessing_only = clone(pipeline.named_steps["preprocess"]).fit(X_train, y_train)
feature_universe = preprocessing_only.get_feature_names_out()
total_splits = args.folds * args.repeats
stability = complete_feature_stability(fold_features, total_splits, feature_universe)
stability.to_csv(output / "feature_stability_all_features.csv", index=False)
group_stability = aggregate_group_stability(
    fold_features, total_splits, stability["feature"].tolist()
)
group_stability.to_csv(output / "reviewer_feature_stability.csv", index=False)
save_latex(
    group_stability,
    output / "table_reviewer_feature_stability.tex",
    "Selection and decision-tree-use stability across repeated cross-validation fits.",
    "tab:feature_stability",
    float_format="%.3f",
)

plot_metric_distributions(cv_results, output)
plot_feature_stability(group_stability, output)
```

```python
bootstrap = bootstrap_oob(pipeline, X_train, y_train, args)
bootstrap.to_csv(output / "bootstrap_oob_metrics.csv", index=False)
if len(bootstrap):
    bootstrap_summary = metric_summary(bootstrap, "Bootstrap OOB")
    bootstrap_summary.to_csv(output / "bootstrap_oob_summary.csv", index=False)


final_model = clone(pipeline).fit(X_train, y_train)
test_prediction = final_model.predict(X_test)
test_score = positive_scores(final_model, X_test, args.positive_class)
test_metrics = calculate_metrics(y_test, test_prediction, test_score, args.positive_class)
pd.DataFrame([test_metrics]).to_csv(output / "independent_test_metrics.csv", index=False)
labels = [str(c) for c in final_model.classes_]
auc_value = plot_test_outputs(y_test, test_prediction, test_score, labels,
                              args.positive_class, output)
report = classification_report(y_test, test_prediction, labels=labels, zero_division=0)
(output / "independent_test_classification_report.txt").write_text(report, encoding="utf-8")
export_final_tree(final_model, output)
```

```
    print("\nRepeated stratified CV summary:\n", cv_summary.to_string(index=False))
    print("\nReviewer-feature stability:\n", group_stability.to_string(index=False))
    print("\nIndependent test metrics:")
    for name, value in test_metrics.items():
        print(f"  {name}: {value:.4f}")
    print(f"  ROC-AUC confirmed by plotted scores: {auc_value:.4f}")
    print(f"\nAll outputs saved to: {output.resolve()}")


if __name__ == "__main__":
    main()
```

Code 2. The Python code implemented to realize the pharmacogenomics investigation methodology.

QQ-Plot

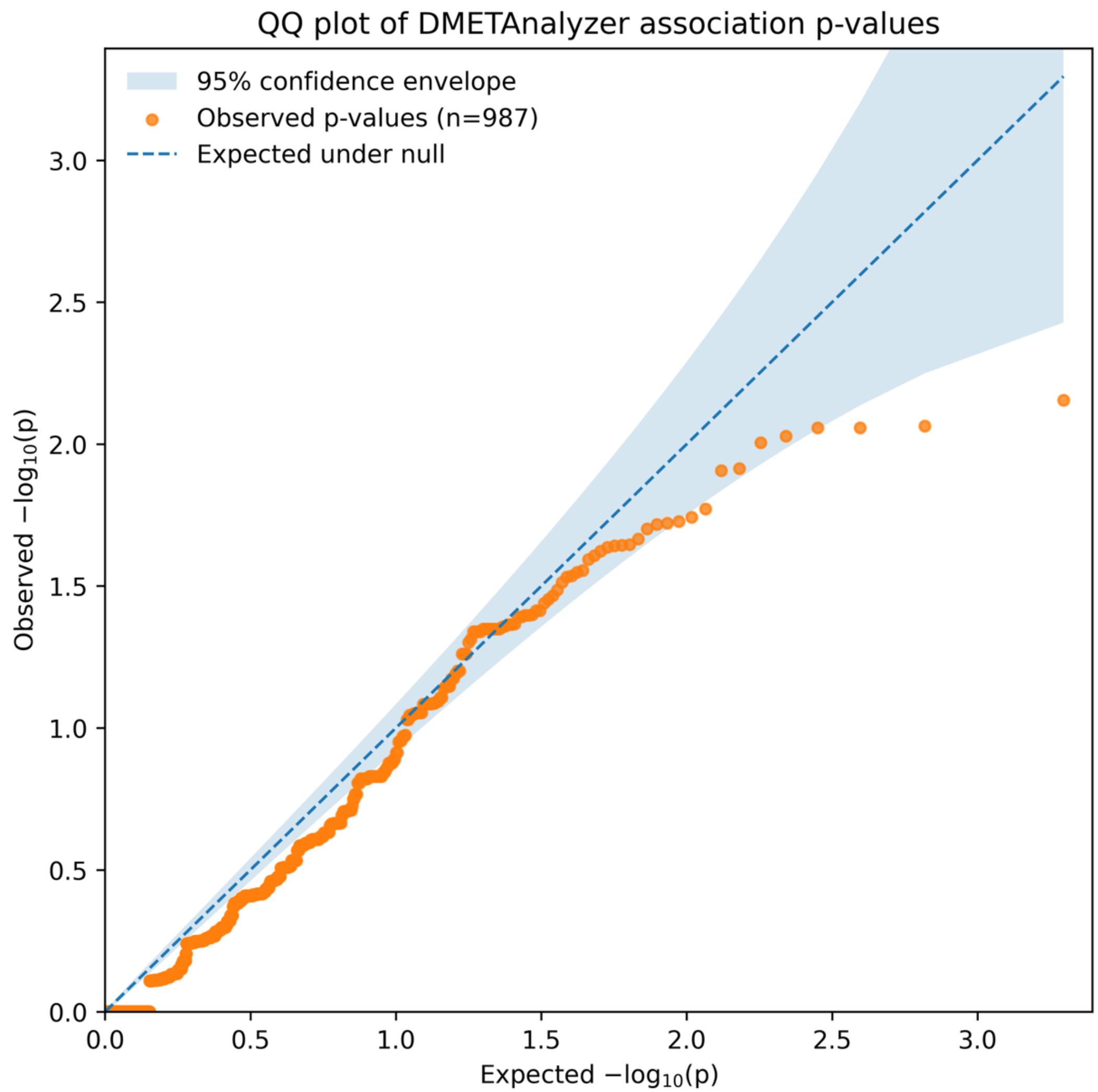

Figure S1. Quantile–quantile (QQ) plot of the uncorrected genotype-association p-values generated by DMETAnalyzer. Observed –log10(p) values from 987 Fisher's exact tests across 348 probe sets were compared with the corresponding

values expected under the null hypothesis. The shaded area represents the 95% pointwise confidence envelope. The overall distribution showed no evidence of systematic inflation of association p-values. Deviations below the expected line are consistent with the conservative and discrete nature of Fisher's exact tests and with the dependence among multiple genotype comparisons performed for some probe sets.
